# 3-Pyridinylidene Derivatives of Chemically Modified Lupane and Ursane Triterpenes as Promising Anticancer Agents by Targeting Apoptosis

**DOI:** 10.3390/ijms221910695

**Published:** 2021-10-02

**Authors:** Oxana Kazakova, Codruța Șoica, Marat Babaev, Anastasiya Petrova, Elmira Khusnutdinova, Alexander Poptsov, Ioana Macașoi, George Drăghici, Ștefana Avram, Lavinia Vlaia, Alexandra Mioc, Marius Mioc, Cristina Dehelean, Adrian Voicu

**Affiliations:** 1 Ufa Institute of Chemistry UFRC, Russian Academy of Science RAS, pr. Oktyabrya 71, 450054 Ufa, Russia; b.marat.c@mail.ru (M.B.); ana.orgchem@gmail.com (A.P.); ElmaH@inbox.ru (E.K.); susbo77@yandex.ru (A.P.); 2 Formulation and Technology of Drugs Research Center, Faculty of Pharmacy, “Victor Babeș” University of Medicine and Pharmacy, Eftimie Murgu Sq. No. 2, 300041 Timisoara, Romania; codrutasoica@umft.ro (C.Ș.); macasoi.ioana@umft.ro (I.M.); draghici.george-andrei@umft.ro (G.D.); stefana.avram@umft.ro (Ș.A.); vlaia.lavinia@umft.ro (L.V.); alexandra.petrus@umft.ro (A.M.); cadehelean@umft.ro (C.D.); 3 Research Centre Pharmacotoxicol Evaluat, Faculty of Pharmacy, “Victor Babeș” University of Medicine and Pharmacy Timisoara, 2nd Eftimie Murgu Sq., 300041 Timisoara, Romania; 4 Formulation and Technology of Drugs Research Center, “Victor Babeș” University of Medicine and Pharmacy, Faculty of Pharmacy, Eftimie Murgu Sq. No. 2, 300041 Timișoara, Romania; 5 Faculty of Medicine, “Victor Babeș” University of Medicine and Pharmacy, 2nd Eftimie Murgu Sq., 300041 Timisoara, Romania; adrian.voicu@umft.ro

**Keywords:** pentacyclic triterpenes, Claisen–Schmidt reaction, anticancer activity, apoptosis, antiangiogenic, molecular docking

## Abstract

Cancer persists as a global challenge due to the extent to which conventional anticancer therapies pose high risks counterbalanced with their therapeutic benefit. Naturally occurring substances stand as an important safer alternative source for anticancer drug development. In the current study, a series of modified lupane and ursane derivatives was subjected to in vitro screening on the NCI-60 cancer cell line panel. Compounds **6** and **7** have been identified as highly active with GI_50_ values ranging from 0.03 µM to 5.9 µM (compound **6**) and 0.18–1.53 µM (compound **7**). Thus, these two compounds were further assessed in detail in order to identify a possible antiproliferative mechanism of action. DAPI (4′,6-diamidino-2-phenylindole) staining revealed that both compounds induced nuclei condensation and overall cell morphological changes consistent with apoptotic cell death. rtPCR analysis showed that both compounds induced upregulation of proapoptotic Bak and Bad genes while downregulating Bcl-XL and Bcl-2 antiapoptotic genes. Molecular docking analysis revealed that both compounds exhibited high scores for Bcl-XL inhibition, while compound **7** showed higher in silico Bcl-XL inhibition potential as compared to the native inhibitor ATB-737, suggesting that compounds may induce apoptotic cell death through targeted antiapoptotic protein inhibition, as well.

## 1. Introduction

Cancer persists as a major challenge to public health worldwide being the main focus of various research and public entities. In Europe, a main priority within the health area is the Europe’s Beating Cancer Plan which establishes new approaches to cancer prevention, treatment, and care [1] in light of the fact that in 2020, there were 2.7 million new cancer cases in the European Union and another 1.3 million cancer-related deaths. In US, The American Cancer Society estimated almost 2,000,000 new cancer diagnosis in 2021 and approximately 600,000 cancer deaths [2]. The International Agency for Research on Cancer, the specialized cancer agency of the WHO, estimates an increase in cancer incidence from 19.3 million in 2020 to 30.2 million in 2040 while cancer-related deaths will rise from 9.96 million cases in 2020 to 16.3 million in 2040 [3]. Overall, for certain types of cancer there has been a decline in both incidence and death, but in 2020 the collected data may be flawed by the coronavirus (COVID-19) pandemic which reduced access to health care; as a result, a false drop in cancer incidence may have been reported, followed in future years by an increase of advanced stage disease and mortality [2].

Cancer treatment is one of the key pillars in cancer research. Despite the tremendous therapeutic successes there are still numerous failures due to drug resistance and adverse effects. Natural products exhibit a wide variety of chemical structures making them a rich pool of potential pharmacologically active compounds; in fact, in the last 40 years, about 25% of the newly introduced anticancer chemotherapeutics were related to natural sources [4], while 60% of the currently used anticancer drugs have natural origins [5].

Triterpenoids are a group of structurally diverse natural compounds that display a large number of distinct chemical scaffolds. Triterpenic acids with lupane, oleanane, and ursane structural cores such as betulinic, oleanolic, and ursolic acids, respectively, exhibit significant anticancer, anti-inflammatory, hepatoprotective, and other activities [6,7,8]. 

Over the past few decades, these compounds have been used as effective molecular templates for the synthesis of compounds with diverse pharmacological activities [9,10]. The functionalizations of betulinic acid and its natural analogs—betulin, betulonic, and 23-hydroxybetulinic acids—through simple chemical transformations or polymer-conjugation were reviewed; most compounds exhibited antiproliferative activity against a large variety of cancer cell lines [11]. The most investigated derivative of oleanolic acid, bardoxolone methyl (CDDO-Me), has reached phase 1 clinical trials as treatment against solid tumors and lymphoma and phase 3 clinical trials for pulmonary arterial hypertension associated to connective tissue disease [8]. A recent study investigated a series of ursolic acid derivatives with significantly improved anticancer activities compared to the native compound; the main sites for chemical modulation were the C28-carboxylic moiety and the C3-β-hydroxylic moiety but miscellaneous groups were introduced as well on the main scaffold [6].

**Figure 1:**
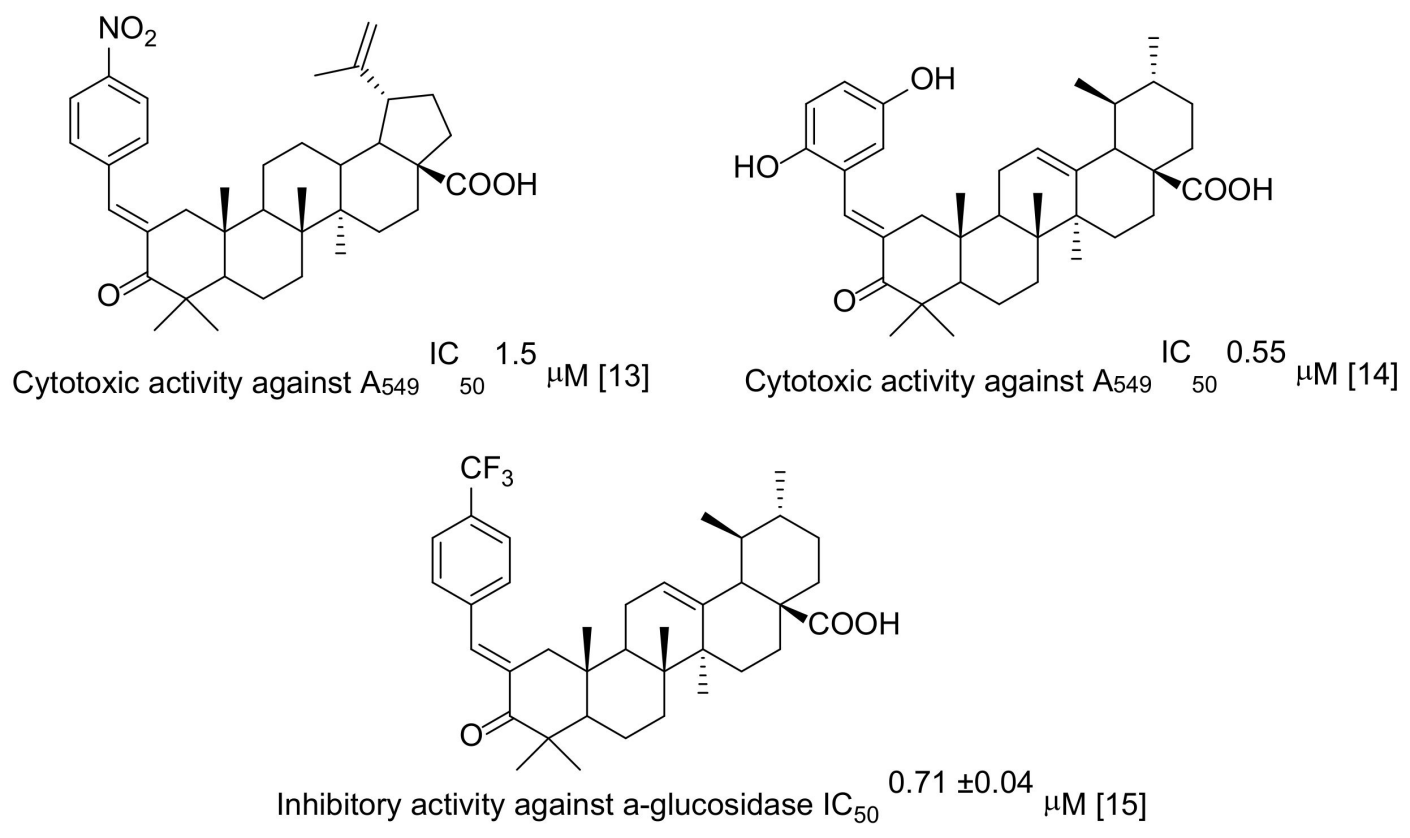
Biologically active triterpene C2-benzylidine derivatives.

Chalcones are distributed widely in fruits, vegetables, spices, tea, and soya-based foodstuffs and exhibit remarkable biological activities [12]. Chemically, they are open-chain precursors of flavonoids and isoflavonoids, in which two aromatic rings are linked by a three-carbon α,β-unsaturated carbonyl system. Numerous studies were devoted to the synthesis of triterpene-benzylidene derivatives, basically consisting in the chemical modification of the C2 position, which led to anticancer [13,14] and antidiabetic activities [15] (Figure 1). 

**Figure 2:**
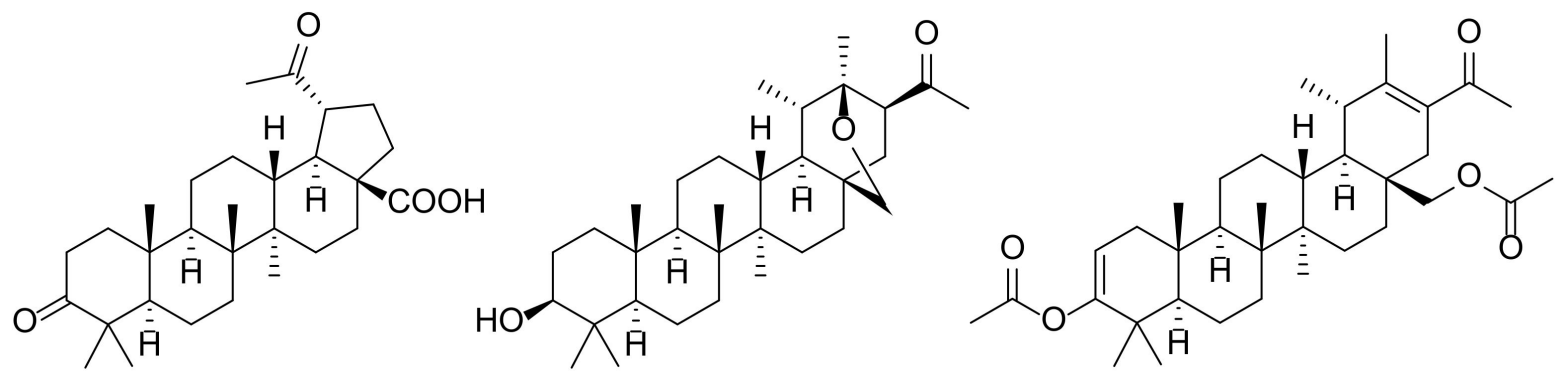
Platanic acid and 18α,19βH-ursane type derivatives as new scaffolds for further modifications toward anticancer agents.

20-Oxo-lupane type triterpenoids, obtained by ozonolysis of the C19-isopropenyl group, are the new scaffolds for synthesis of chalcone derivatives. In a recent study, C30-derivatives of platanic acid and messagenin were obtained, among them 2,30-bis-(3-pyridinylidene)-platanic acid and 3-pyridinylidene-messagenin exhibited a broad spectrum of anticancer activity against NCI-60 with GI_50_ range 1–2 μM [16]. By the rearrangement of allobetulone E-ring we have synthesized a unique ursane type triterpenoids with an acetyl fragment at C21 position [17,18,19,20] (Figure 2).

In this study, a series of new triterpenoids were synthesized from methyl 3-oxo-platanoate and 3-oxo-21β-acetyl-20β,28-epoxy-18α,19βH-ursane using Claisen–Schmidt condensation with 3-pyridine carboxyaldehyde and furfural at the positions C30 and C31, respectively. The synthesized compounds were later evaluated for their antiproliferative activity, and the molecules with the highest effect were subsequently investigated to determine a possible mechanism of action.

## 2. Results and Discussion

### 2.1. Chemistry

**Scheme 1:**
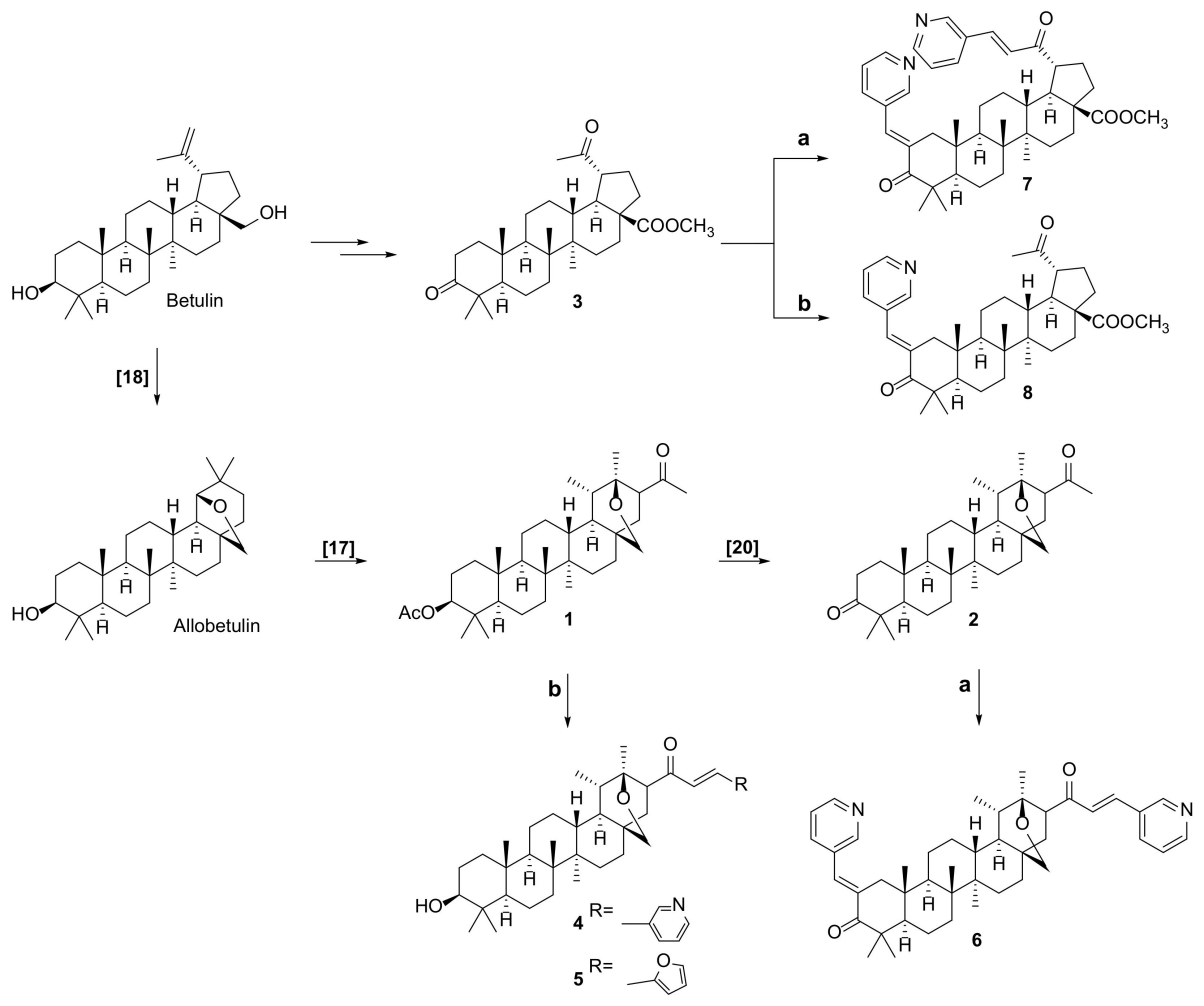
Reagents and conditions: **a**. 2 eq. of 3-pyridine carboxaldehyde, 20% KOH in EtOH, EtOH, 12 h, 25 °C; **b**. 1 eq. of 3-pyridine carboxaldehyde or furfural, 20% KOH in EtOH, EtOH, 24 h, 25 °C.

The synthetic route to a series of new triterpene derivatives **4**–**8** from betulin as starting platform is outlined in Scheme 1. Compound **1** was obtained by allobetulone rearrangement with HClO_4_ in Ac_2_O as previously described [17]. Compound **3** was synthesized by subsequent reactions of Jones oxidation, C28-methylation, and ozonolysis of C20(29) double bond. Compounds **4**–**8** were obtained by Claisen–Schmidt condensation of compound **1**, **2**, or **3**, respectively, with 3-pyridine carboxaldehyde or furfural in EtOH in yields of 80–85%. The reaction of methyl 3,20-dioxo-lup-28-oate (derivative of platanic acid) with 2 eq. of 3-pyridine carboxaldehyde led to the formation of 2,30-bispyridinylidene derivative **7** (yield 82%), while the interaction with 1 eq. of aldehyde led to the C2-monosubstituted derivative **8** with yield of 80%. The synthesis of these compounds demonstrates the occurrence of a regioselective process presumably due to the steric hindrances at C30 position. In the case of compound **2**, condensation with 3-pyridine carboxaldehyde (1–2 equations) was not selective and led to the 2,21-bispyridinylidene derivative **6** (yield 80%). 21-Monosubstituted derivatives **4** and **5** were synthesized by a reaction of the ursane triterpenoid **1** with 3-pyridine carboxaldehyde or furfural, accompanied by C3-deacetylation with yields of 84 and 85%, respectively.

**Figure 3:**
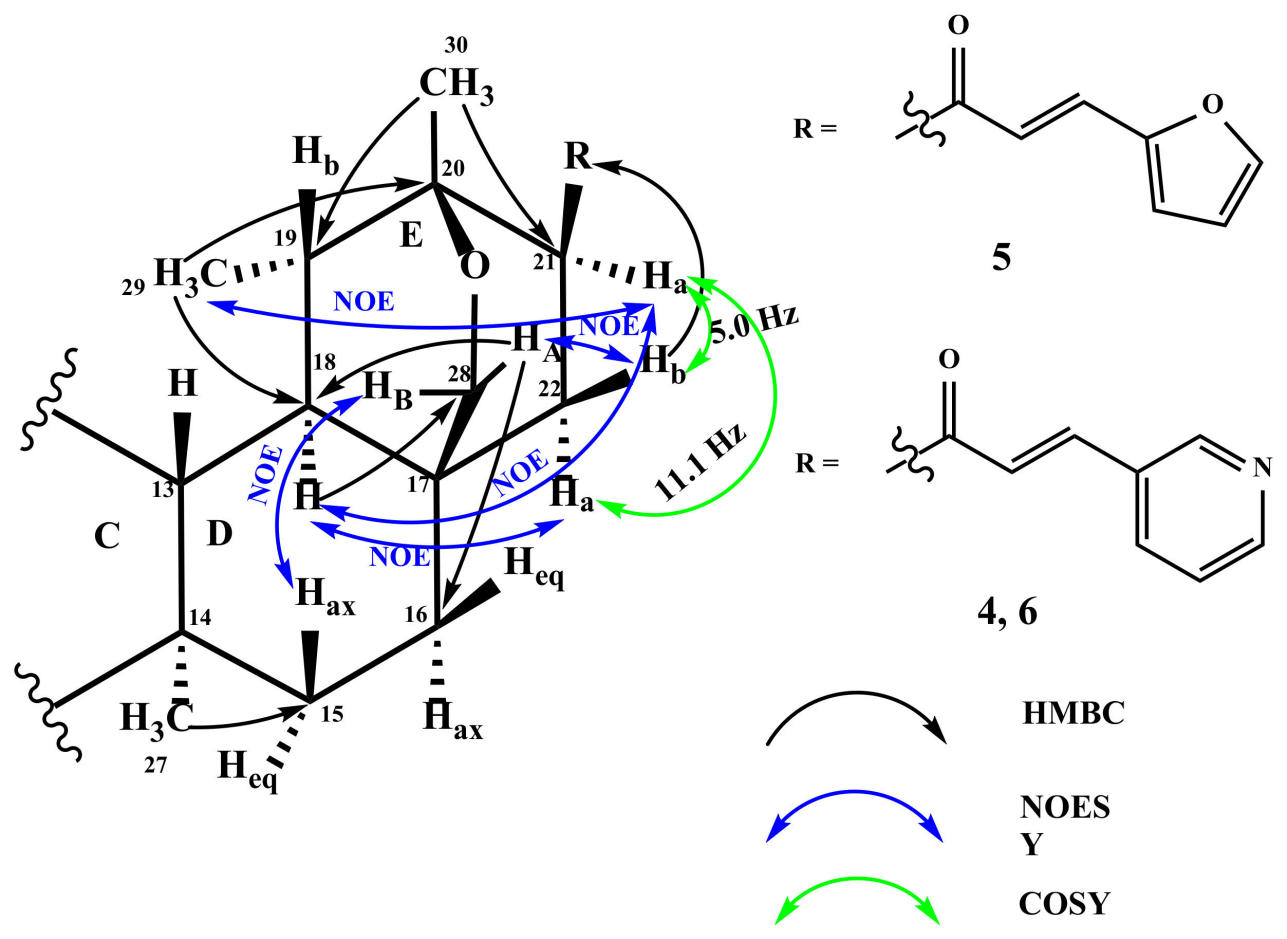
The key HMBC, COSY and NOESY correlations of compounds **4–6**.

The structure of compounds **4**–**8** was confirmed by ^1^H and ^13^C NMR spectroscopy data (for NMR spectra see Supplementary Material Figures S1–S5). The stereochemistry of the 3-pyridinylidene and furfurylidene fragments at C21 for compounds **4**–**6** was confirmed according to doublet-doublet signal ^3^J constant of H21 (11.1 and 5.0 Hz). According to the work in [21], the value of 10–11 Hz of constants is characteristic of the syn-periplanar position of endo-oriented protons in the 2-oxobicycle[2.2.2]octane fragment, that indicated the β-orientation of substituents at C21. In the NOESY spectra, the cross peaks between the H21, H_α_-22, and H18 protons with C29 methyl group confirmed its α-orientation. For protons in the head of the H_A_-28 and H_B_-28 bridge, long-range W binding to the protons H18 and H_α_-22 are observed, respectively. The value ^4^J_28A-18_ = 1.2 Hz and ^4^J_28B-22α_ = 3.0 Hz indicated the anti (for H_A_-28) and syn (for H_B_-28) proton orientation at A-D rings of the triterpene core, that confirmed according to NOE correlation between H_A_-28/H_eq_-16, H_A_-28/H_β_-22, and H_B_-28/H_ax_-15, H_B_-28/H-19 (Figure 3).

**Figure 4:**
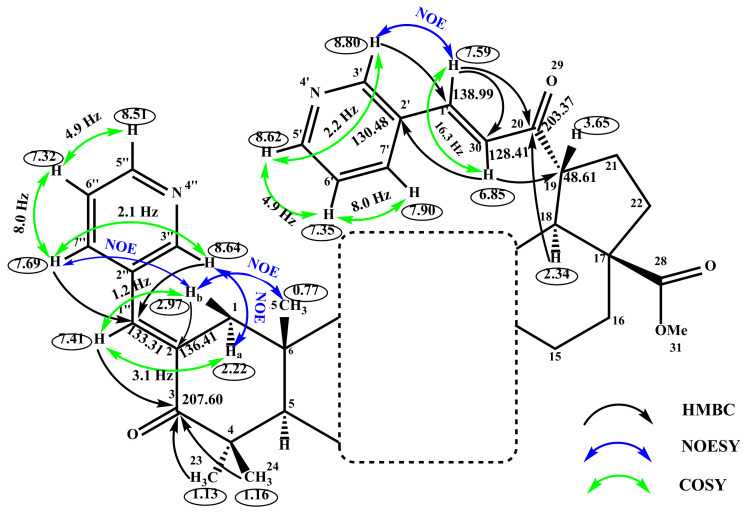
HMBC, COSY, and NOESY correlations for compound **7**.

The E-configuration of double bond in 3-pyridinylidene and furfurylidene fragment at C21 was established from the value of ^3^J_32-1′_ = 15.9 Hz, that indicated trans arrangement of protons at double bond. A similar situation is observed for compound **7**. The E-configuration of double bond in 3-pyridinylidene fragment at C19 was established from the value of ^3^J_30-1′_ = 16.0 Hz (Figure 4).

**Figure 5:**
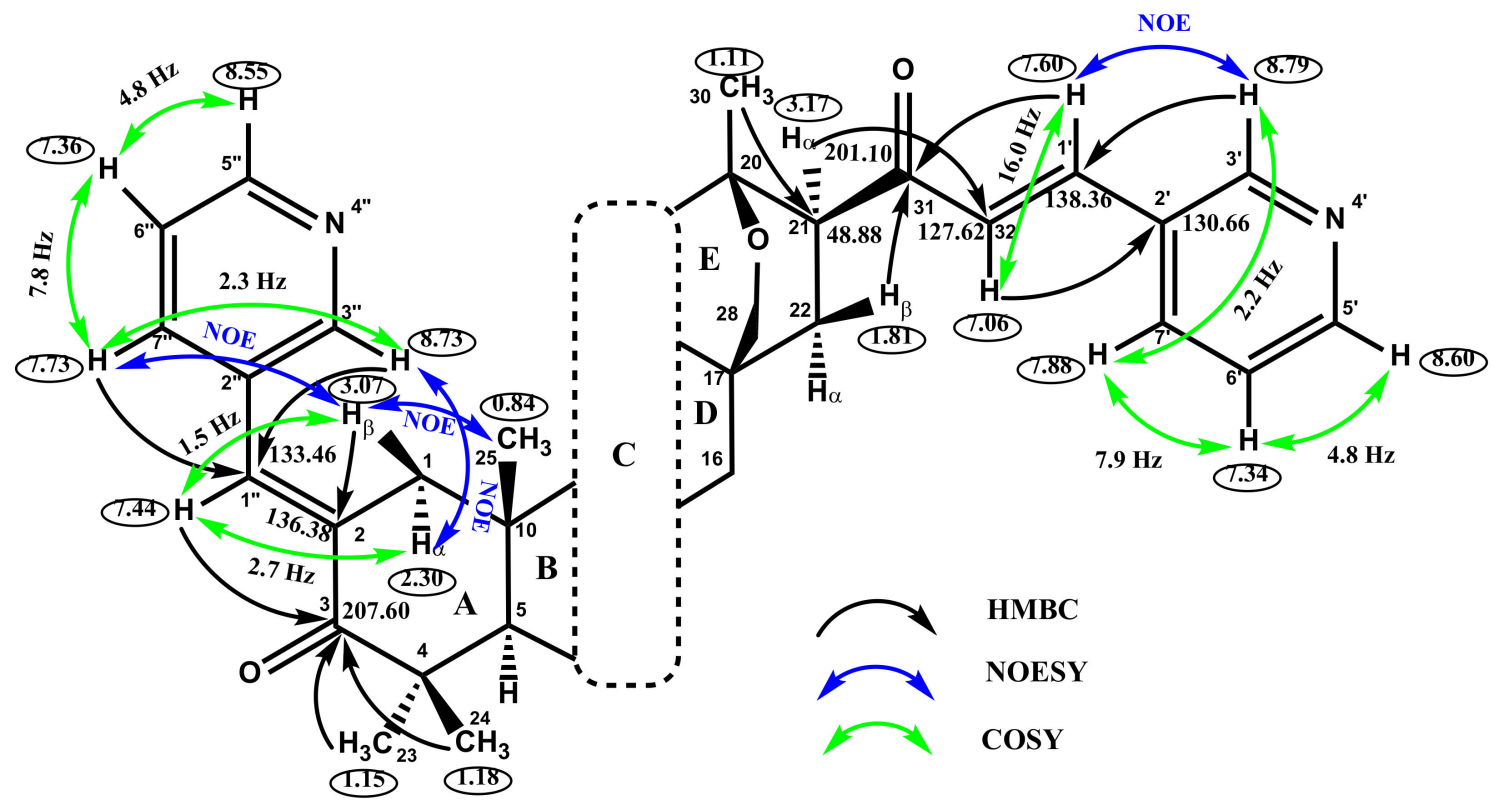
HMBC, COSY, and NOESY correlations for compound **6**.

The formation of C2(1″)-double bond for compounds **6** and **7** and a C2(1′)-double bond for compound **8**, respectively, was confirmed by the HMBC cross-peaks of protons methylene group C1 of A-ring at H_a_-1 and H_b_-2 with a signal of C2-carbon (δ_C_ 136.41 ppm), as well as the HMBC interaction between protons H1″(1′) (δ_H_ 7.41 ppm) with a signal of carbonyl carbon C3 (δ_C_ 207.60 ppm). The E-configuration of C2-C1″(C1′)-unsaturated bond for compounds **6**–**8** was confirmed based on NOESY cross-peaks of doublet signal H3″(3′) at δ_H_ 8.65 ppm of 3-pyridinyl fragment with protons of methylene group C1 of A-ring at δ_H_ 2.24 and 2.98 ppm (Figure 5). 

### 2.2. Biological Evaluation

#### 2.2.1. In Vitro Screening against NCI-60 Cell Line Panel 

The new 20-oxo-lupane and ursane derivatives 1, 2, 4-8 were selected by the National Cancer Institute (NCI) [22,23] for the in vitro cell line screening. The National Cancer Institute established in the early 1990s a high-throughput systematic screening program on a panel of 60 cancer cell lines (NCI-60) emerging from multiple types of tumors; the screening was used over the last decades on more than 100,000 compounds and 50,000 natural extracts in order to assess their tumor growth inhibition [24,25].

At first, compounds were tested in a single high dose concentration regimen (10 µM) against a panel of sixty various cancer cell lines; the results of the screening are outlined in Table 1 in the form of percent growth (GP %) of treated cells versus untreated cells used as control, where negative numbers indicate cell kill. The experimental results of growth inhibition may range from 0 to 100 and indicate a cytostatic effect while negative values indicate cell death and thus a cytotoxic activity (for NCI data see Supplementary Material Figures S6–S12).

One can notice that the introduction of 3-pyridinylidene substituent on the pentacyclic triterpene scaffold resulted in a slight improvement in antiproliferative properties compared to the starting compound **1**, which completely lacks activity. The presence of the furfurylidene fragment in compound **5** led to selective anticancer activity against colon cancer (COLO 205 −2.30%) and melanoma (MALME-3M −11.31%; UACC-257 −8.91%). Compound **8** bearing one 3-pyridinylidene fragment demonstrated moderate cytotoxicity against leukemia and colon cancer cell lines. 

Compounds **6** and **7**, which showed the highest cytotoxic activity, were further investigated in a five-dose testing regimen and exhibited significant antiproliferative activity with GI_50_ ranging from 0.03 µM on leukemia cell lines to 5.9 µM on ovarian cancer cell lines, but with similar GI_50_ values against all panels of NCI-60 thus exhibiting a wide anticancer spectrum (Table 2, for NCI data see Supplementary Material Figures S13 and S14). Compound **7** exhibited almost the same remarkable antitumor activity against all types of cancer cell lines, with GI_50_ values varying between 0.18 µM against renal cancer and 1.53 µM against prostate cancer; however, all GI_50_ values recorded on all tested cancer cell lines are comparable to each other, also indicating a wide spectrum of anticancer activity. Furthermore, an activity comparison of compounds **6** and **7** with doxorubicin, used by NCI as standard drug control, reflects that the GI_50_ values were comparable with the standard drug and higher against 4 types of cancer cell lines (leukemia, colon cancer, prostate cancer, and ovarian cancer). A comparison of the cytotoxic activity of the synthesized derivatives shows precedence over the starting compounds **1**–**3**. An advantage of 18α,19βH-ursane type triterpenoids **4**–**6** follows from the data of Table 1, while a low level of cytotoxic activity of platanic acid scaffold (a precursor of compound **3**) (GI_50_ > 50 μM) is known from the literature data [26,27,28]. Moreover, methyl 2,30-bis-(3-pyridinylidene)-3,20-dioxo-lup-28-oate 7 (Table 2) demonstrates lower GI_50_ values than its C17-acid-analog [16] toward NCI-60 cancer cell panel in a five-dose testing. 

The cytotoxicity of compounds **6** and **7** was also evaluated on healthy cell lines (HaCaT—human keratinocytes and 1BR3 human fibroblasts). The obtained results show GI_50_ values >15 μM indicating a relatively low cytotoxicity towards non-cancer cells as compared to the effect exerted on all other cancer cells tested.

The selectivity index (SI) was calculated by dividing the full panel MG_MID60 (μM) of tested compounds by their individual subpanel MG_MID of the cell line (μM); it correlates directly to the selectivity of their anticancer activity (Table 3). SI values ranging from 3 to 6 indicate moderate selectivity while values higher than 6 are characteristic for highly selective compounds against the respective cell line; all compounds that do not meet either of these criteria are categorized as nonselective [29]. One can notice that compound **6** exhibits moderate to high selectivity on leukemia cell lines, with SI = 5.30. By contrast, despite its strong antiproliferative effect, compound **7** acts with low selectivity against the various human tumor cell lines, the highest SI value being 1.73.

#### 2.2.2. Compounds **6** and **7** Induce Nucleus Condensation of A375, RPMI, and SK-MEL-28 Melanoma Cell Lines 

Due to the high antiproliferative activities of compounds **6** and **7**, and considering that compound **7** (most active compound) exhibited the highest overall antitumor activity on the melanoma cell lines, the two candidates were selected for the investigation of their more intimate cytotoxic mechanisms by using the DAPI staining procedure, on three different melanoma cell lines. For this purpose, the melanoma cell lines selected were A375, RPMI, and SK-MEL-28, given that these three lines have very good correlation coefficient scores with tumor cells originating from a wide range of malignant tumors, based on transcriptional similarity. This feature can also lead to increased clinical applicability potential for the tested substances [30]. The two compounds were tested against three human melanoma cell lines: A375, RPMI, and SK-MEL-28 melanoma cell lines by applying 0.1, 1, and 5 μM solutions, respectively. 4′,6-Diamidino-2-phenylindole (DAPI) is a DNA-specific marker which allows the direct fluorescent blue staining of nuclei against a dark background, widely used in life sciences research [31]. In effect, the DAPI stain binds to chromatin within the nuclei and provides their clear outline under the blue light (405 nm) while the cytoplasmic medium remains non-fluorescent [32]. Moreover, due to its lack of interference with DNA–linker interactions, DAPI stain can provide fine details of nuclei’ shapes instead of merely highlighting their centers which contain the highest concentration of nucleic acids [33].

**Figure 6:**
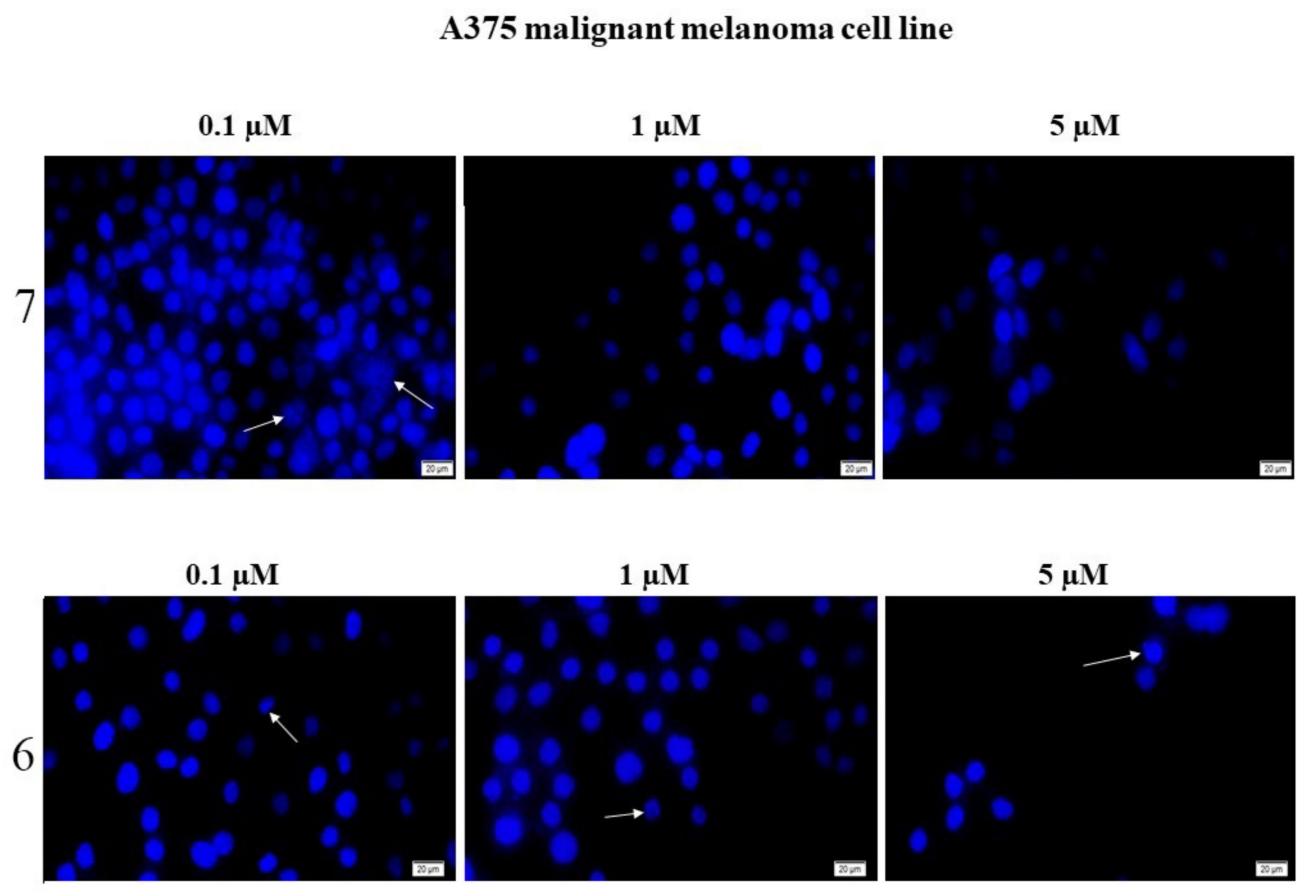
Effect of compounds **6** and **7** (0.1, 1 and 5 uM) and DMSO (0.1, 1 and 5 uM) after 24 h of stimulation on the A375—DAPI staining. The white arrows indicate the apoptotic cells with nuclear fragmentation; figures are scaled at 20 μm.

**Figure 7:**
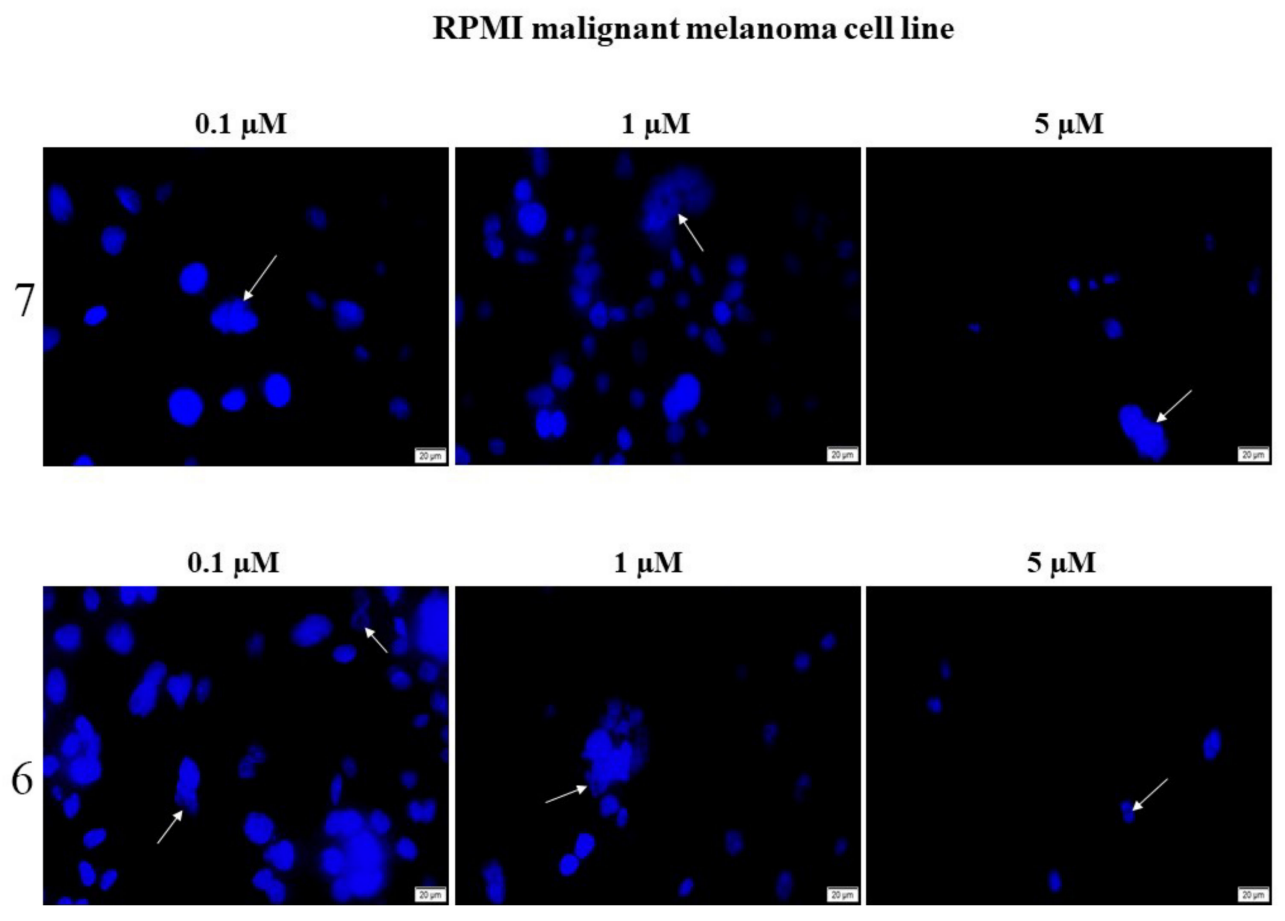
Effect of compounds **6** and **7** (0.1, 1, and 5 uM) and DMSO (0.1, 1, and 5 uM) after 24 h of stimulation on the RPMI—DAPI staining. The white arrows indicate the apoptotic cells with nuclear fragmentation; figures are scaled at 20 μm.

**Figure 8:**
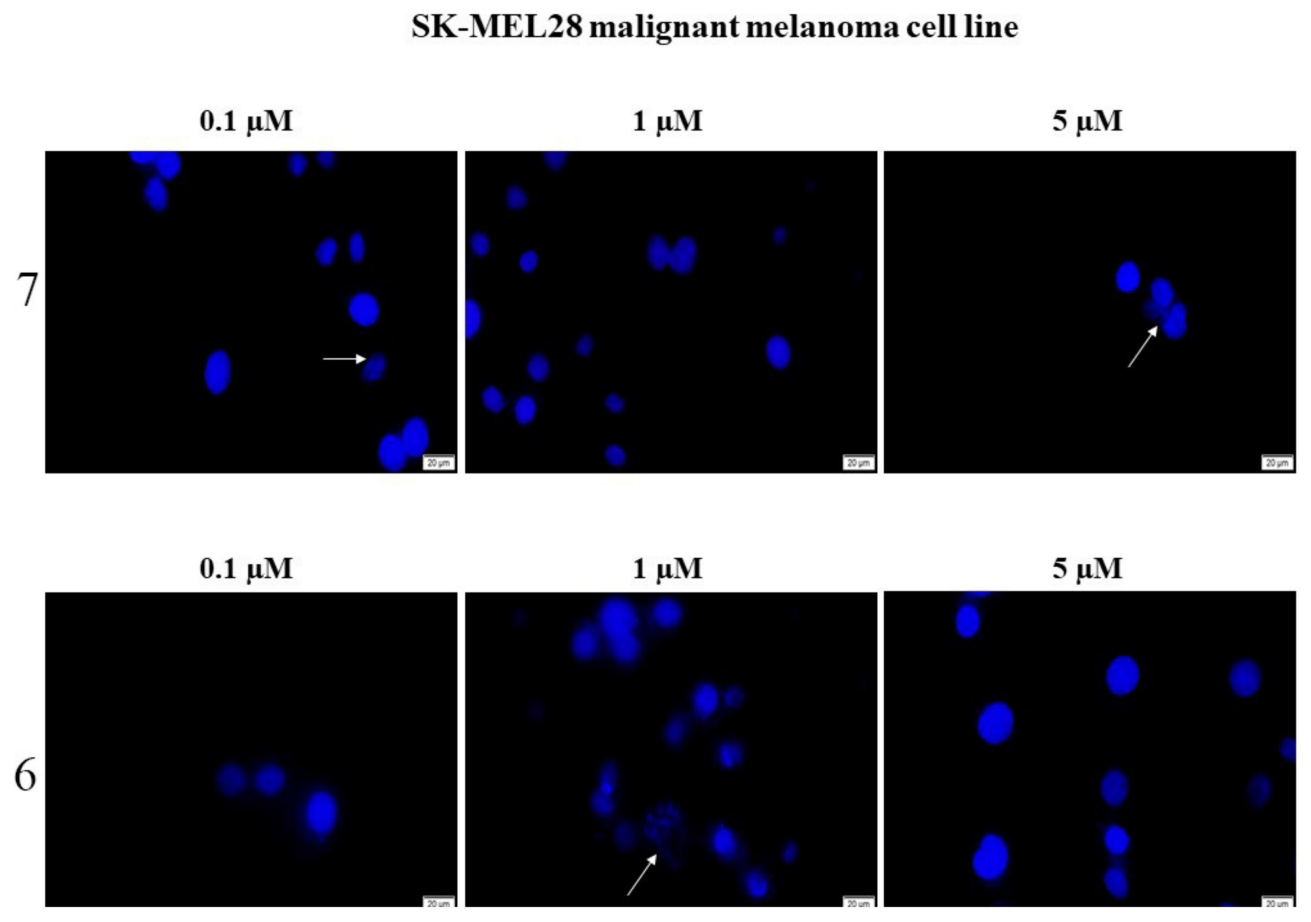
Effect of compounds **6** and **7** (0.1, 1, and 5 uM) and DMSO (0.1, 1, and 5 uM) after 24 h of stimulation on the SK-Mel-28—DAPI staining. The white arrows indicate the apoptotic cells with nuclear fragmentation; figures are scaled at 20 μm.

The DAPI staining procedure revealed the nuclei condensation for both compounds in A375, RPMI, and SK-Mel-28 melanoma cells, respectively, in a dose-dependent manner; thus, the highest concentration, 5 μM, induced the strongest condensation process. When tested on the human melanoma cell line—A375, the lowest concentration (0.1 μM) of compound **7** induced nucleus fragmentation (indicated with the white arrow in Figure 6); at higher concentrations, the nucleus changes its shape, becoming round and a condensation of chromatin can be noticed. Compound **6** caused nuclear fragmentation when applied as 0.1 and 1 μM samples, while at 5 μM concentration the chromatin becomes condensed, thus indicating cellular apoptosis. In terms of their effects against the RPMI (Figure 7) and SK-Mel-28 (Figure 8) human melanoma cell lines, the two compounds also caused morphological changes in the nucleus, characteristic for cellular apoptosis. Compound **7** (0.1 μM) induced a slight nucleus fragmentation (illustrated by the white arrows), while 1 μM and 5 μM concentrations, respectively, triggered visible changes in the nucleus such as nuclear fragmentation and chromatin condensation. The application of the lowest tested concentration of compound **6** led to a massive nucleus condensation and fragmentation while the 5 μM sample induced the occurrence of nuclear residues. 

#### 2.2.3. Compounds **6** and **7** Induce Changes in Cell Morphology

**Figure 9:**
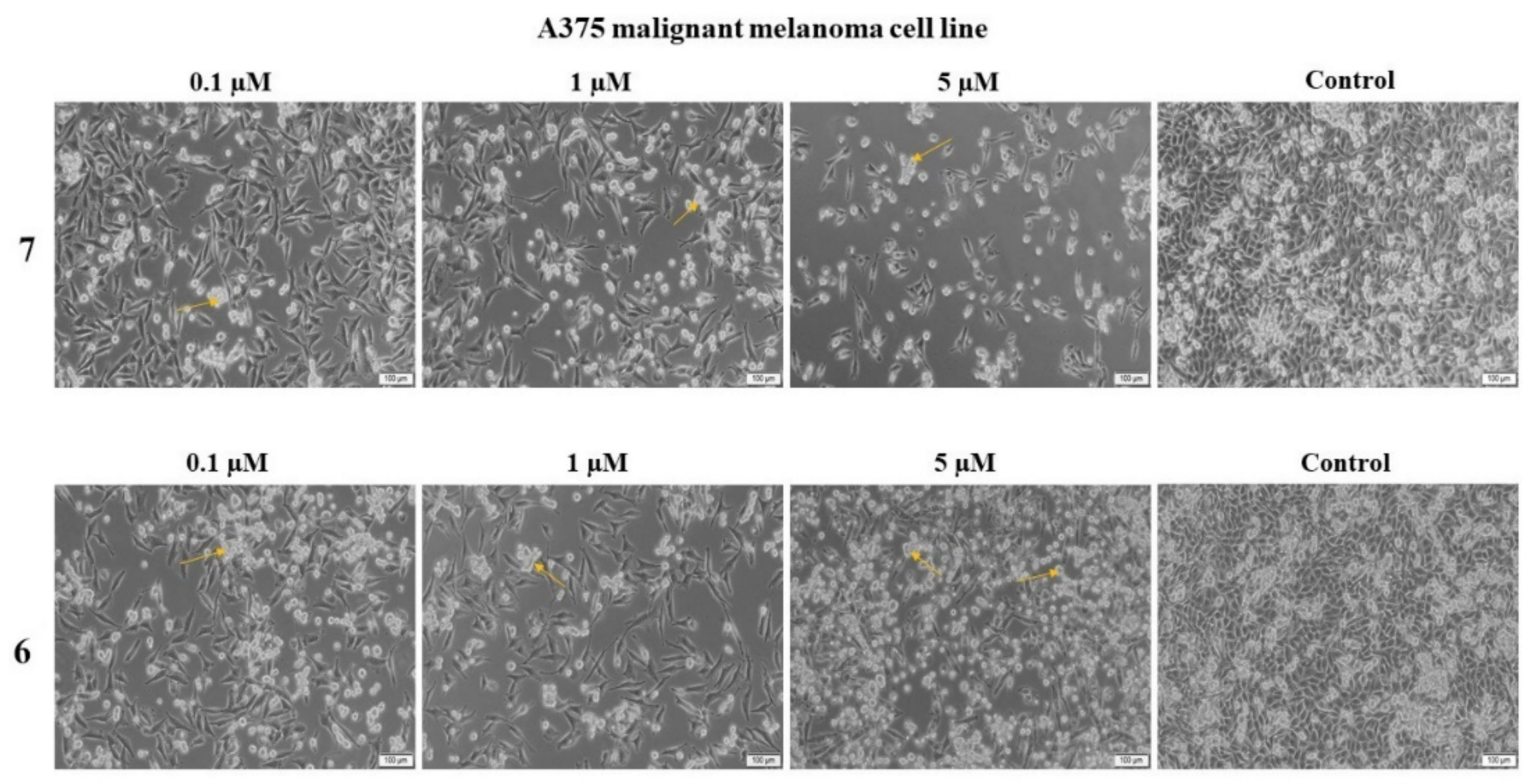
Morphological aspect of the A375 cells after the treatment with compounds **6** and **7** (0.1, 1, and 5μM) for 24 h. The arrows indicate the round and detached cells noticed following the treatment with compounds **6** and **7**. The scale bars represent 100 μm.

In order to further investigate the biological effects of these compounds against three human melanoma cell lines, their effect on cell morphology was assessed. The solvent (DMSO) was used as negative control and did not cause any nucleus alterations even when the highest concentration was used. Both compounds induced morphological changes consistent with apoptotic cell death when applied on the A375 melanoma cell line (Figure 9); one can notice that even in low concentration (0.1 μM), the two compounds induced the occurrence of floating round cells, detached from the plate, thus indicating the involvement of apoptotic mechanisms of cell death [34]. Following the application of the highest 5 μM concentration, the number of round and floating cells is visibly higher thus indicating that the apoptotic effect increases in a concentration-dependent manner.

**Figure 10:**
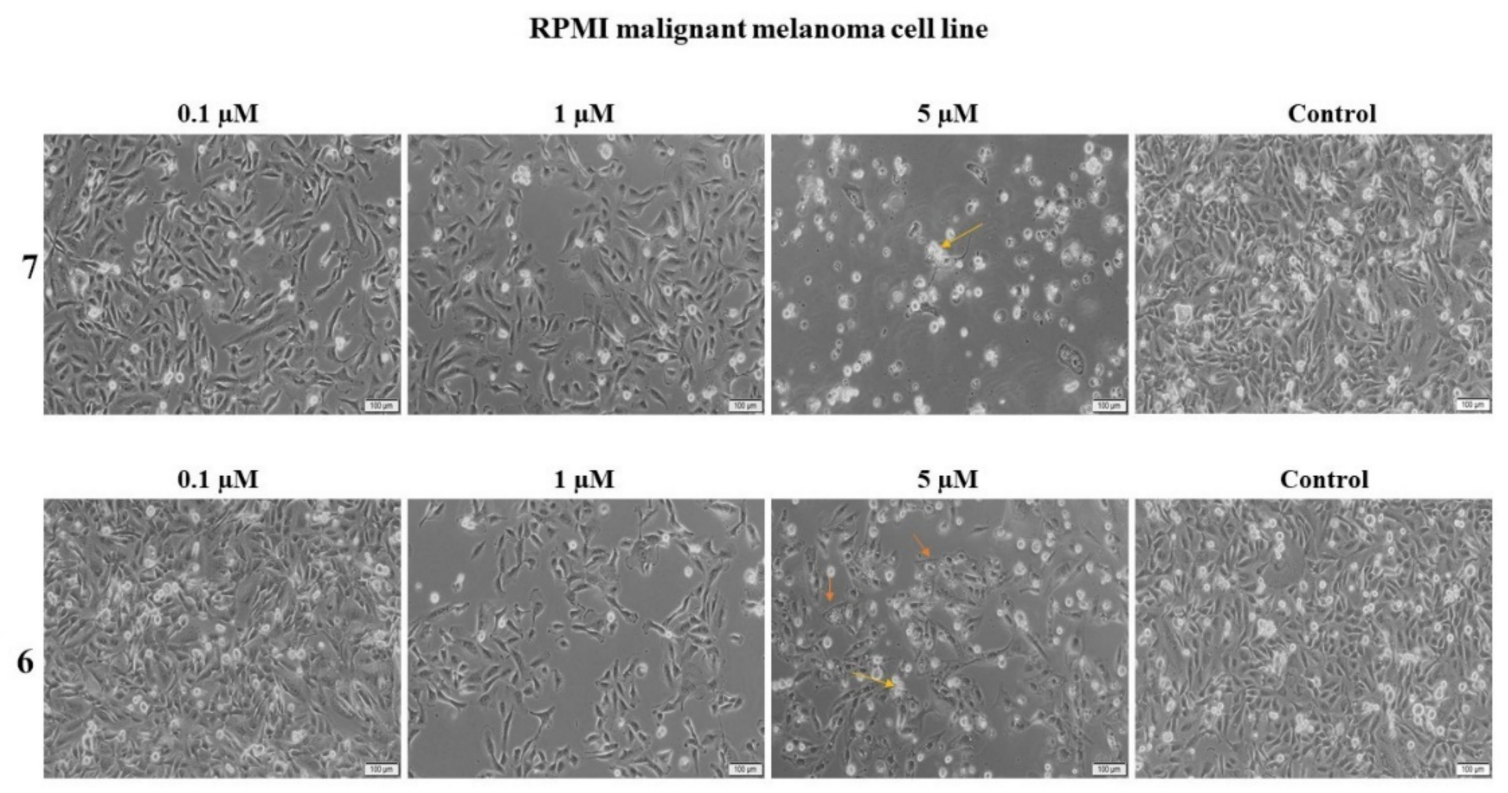
Morphological aspect of the RPMI cells after the treatment with compounds **6** and **7** (0.1, 1, and 5 μM) for 24 h. The yellow arrows indicate the round and detached cells, and the orange arrows indicate the vacuoles noticed following the treatment with compounds **6** and **7**. The scale bars represent 100 μm.

In terms of the RPMI melanoma cell line (Figure 10), a decrease in the number of cells can be observed in direct proportion to the tested concentration; for both compounds, a proapoptotic change occurred in the cellular morphology. Thus, the cells exhibited a rounder shape and became detached from the plate; in addition, for compound **6** used as 5 μM sample, one can notice the formation of vacuoles within the cell cytoplasm (indicated by orange arrows). This effect reveals the pronounced toxic effect of compound **6** against the RPMI human melanoma cell line.

**Figure 11:**
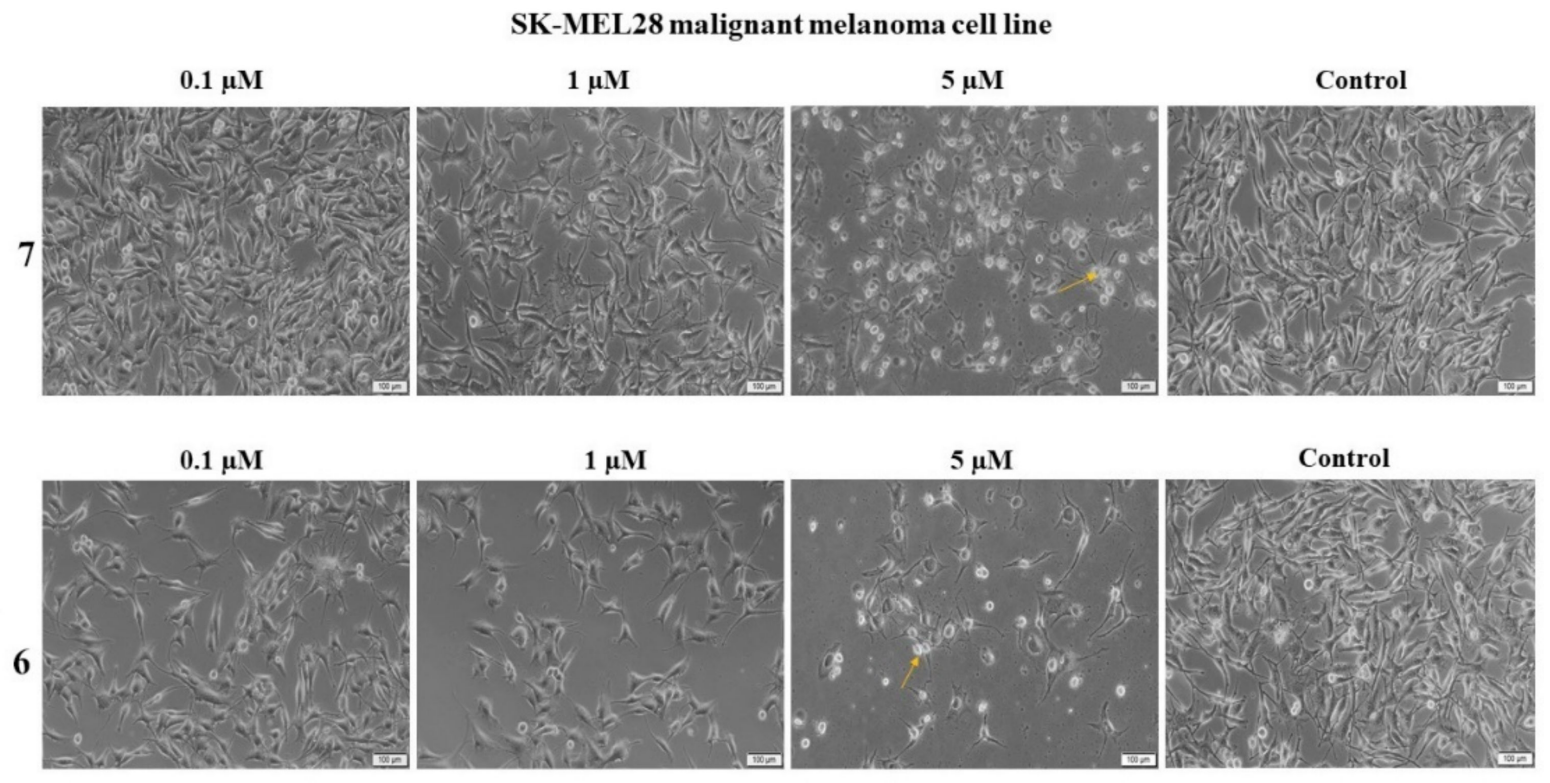
Morphological aspect of the SK-Mel-28 cells after the treatment with compounds **6** and **7** (0.1, 1, and 5 μM) for 24 h. The arrows indicate the round and detached cells noticed following the treatment with compounds **6** and **7**. The scale bars represent 100 μm.

When the two compounds were applied on the SK-Mel-28 human melanoma cell line (Figure 11), the two lower concentrations, 0.1 μM and 1 μM, did not induce visible effects on cell morphology; however, the highest 5 μM concentration caused visible changes at cellular level, the cells becoming round and detached from the plate. Furthermore, a significant decrease of cell number was recorded compared to control.

Cell death is one of the fundamental life processes contributing to the maintenance of homeostasis and eliminating damaged cells [35]. Among various mechanisms of cell death, apoptosis is a programmed process consisting of a succession of controlled events which lead to cell shrinkage and membrane blebbing as well as internal alterations such as DNA condensation. Evasion of apoptosis is considered one of the hallmarks of cancer [36]. Numerous cancer therapies aim to trigger apoptosis, but the cell type and specific molecular aberration strongly influence cell death mechanisms [37]. Therefore, it is important to correctly differentiate apoptosis from necrosis, which is a non-programmed cell death that might occur during cytotoxic treatments, due to the fact that, unlike apoptosis, necrosis causes cell lysis and generates inflammatory responses [38]. Our DAPI protocol highlighted the presence of normal, large nuclei in untreated cells used as control while the treated cells exhibited shrunken and marginated nuclei, indicators of an apoptotic process. 

Our results are in agreement with previously published studies for platanic and betulinic acid derivatives that induced various mechanisms of cell death, mainly apoptosis in melanoma, breast, lung, and ovarian cancer cells [27]; thus, platanic acid was proposed as a new scaffold for the synthesis of new anticancer compounds with wide spectrum [39]. The cytotoxic activity of platanic acid amides was revealed to evolve through apoptotic mechanisms against a panel of human tumor cell lines [28]. A recent review described all main semisynthetic derivatives of ursolic acid, reported in the last 5 years, which interfere with numerous cell signaling pathways and inhibit cancer cells by inducing apoptosis [6]. Briefly, the ursane derivatives revealed superior pharmacokinetic profiles with optimized bioavailability accompanied by low toxicity; in addition, the main identified molecular mechanism in terms of antiproliferative activity was the mitochondria-mediated apoptosis.

#### 2.2.4. Compounds **6** and **7** Induce Downregulation and Upregulation of Genes Depending on the Type of Cell Line Assessed

The Bcl-2 family genes are localized in intracellular membranes such as mitochondria or translocate cytoplasm to mitochondria as a result of triggering cell death; they encode prosurvival, proapoptotic, and divergent proteins [40]. The Bcl-2 proteins share homologous BH domains that facilitate inter-member interactions which regulate apoptosis through the continuous competition between the pro- and antiapoptotic members [41]. Alterations in the Bcl-2 proteins family, either over or under expression, were identified as hallmarks of cancer while agents triggering Bcl-2 family members are currently investigated as anticancer therapy [42]. 

We applied rtPCR techniques in order to investigate the effect of our two compounds on the anti-apoptotic Bcl-XL and Bcl-2 genes as well as the proapoptotic Bak and Bad genes (Table 4). The results are rather controversial; on A375 cells, compound **7** induces the upregulation of both pro- and antiapoptotic genes, while compound **6** downregulated the expression of Bak and Bad proapoptotic genes. Therefore, we may presume that the apoptotic mechanism of both compounds against the A375 cells is not mediated by the tested genes or that alterations in their ratio are the underlying mechanism. In RPMI cells, both compounds downregulated the antiapoptotic Bcl-XL and Bcl2 genes thus facilitating mitochondrial permeability and cytochrome C release and revealing the ability to overcome antiapoptotic drug resistance. The most significant results occurred in SK-Mel-28 melanoma cells, where both compounds induced the down-regulation of the antiapoptotic Bcl2 and Bcl-XL genes and the upregulation of the proapoptotic Bak and Bad genes; thus, the ratio between the proapoptotic versus the antiapoptotic genes changes, favoring the prevalence of the apoptotic process and cell death. 

The literature reports that one ursane derivative, isopropyl 3β-hydroxyurs-12-en-28-oate, induces the downregulation of Bcl-2 protein and causes mitochondrial membrane potential loss and subsequent apoptosis through the production of intracellular ROS [43]. Ursolic acid and some of its analogues such as asiatic or pomolic acids were reported to overcome apoptosis resistance due to the modulation of the Bcl-2 expression; asiatic acid induced apoptosis in SK-MEL-2 cells through the alteration of the Bax/Bcl-2 ratio while pomolic acid induced decreased concentrations of the antiapoptotic proteins Bcl-2 and Bcl-XL [44]. Controversially, betulinic acid induced increased levels of Bad but did not influence the level of Bcl-XL; however, the ratio Bcl-xL/Bad decreased therefore leading to increased apoptosis [45].

Collectively, the revealed apoptotic mechanism of these compounds may occur through the regulation of the Bcl2 family of proteins, in a similar manner with other previously published data; however, other mechanisms may be involved as well therefore requiring further studies.

Apoptosis is a vital component of various biological processes that include normal cell turnover, immune system development and function, hormone-induced atrophy, embryonic development, and chemical-induced cell death. Deregulated apoptosis is often corelated with many disorders including various types of cancer [46]. Given the weight of this cellular process and the fact that this function is altered in tumor cells, apoptosis can be exploited as a multi-level “target” for the development of new antiproliferative therapies. Proapoptotic substances act in several ways that can trigger programmed cell death. One of the main mechanistic pathways corelates to the effect of active compounds on genes that regulate the biosynthesis of pro and antiapoptotic proteins. On a different note, these substances can also interfere with cellular processes that can activate the intrinsic or extrinsic pathway. Last, compounds with proapoptotic effect can act by inhibiting anti-apoptotic proteins (BCL-2 proteins) or other enzymatic pathways that are closely related to apoptotic transmission [47].

#### 2.2.5. Molecular Docking

Molecular docking is a useful computational technique for drug research that can be employed to elucidate the protein targeted action mechanism of a pharmacologically active substances [48,49,50]. Following the obtained RT-PCR analysis results, we looked at whether compounds **6** and **7** exhibit not only gene regulation based proapoptotic effect, but also by direct targeting of proteins involved in apoptosis, cell survivability and proliferation. Using molecular docking, we analyzed the possibility of compounds **6** and **7** to act as a potential inhibitors of protein targets such as: apoptosis regulator Bcl-X (Bcl-XL), apoptosis regulator Bcl-2 (Bcl-2), induced myeloid leukemia cell differentiation protein (Mcl-1), phosphatidylinositol 4,5-bisphosphate 3-kinase catalytic subunit gamma isoform (PI3Kγ), dual specificity mitogen-activated protein kinase kinase 1 (MEK1), mammalian target of rapamycin (mTOR). Docking scores of compounds **6** and **7** along with docking scores of each targets native ligand (controls) are listed in Table 5.

**Figure 12:**
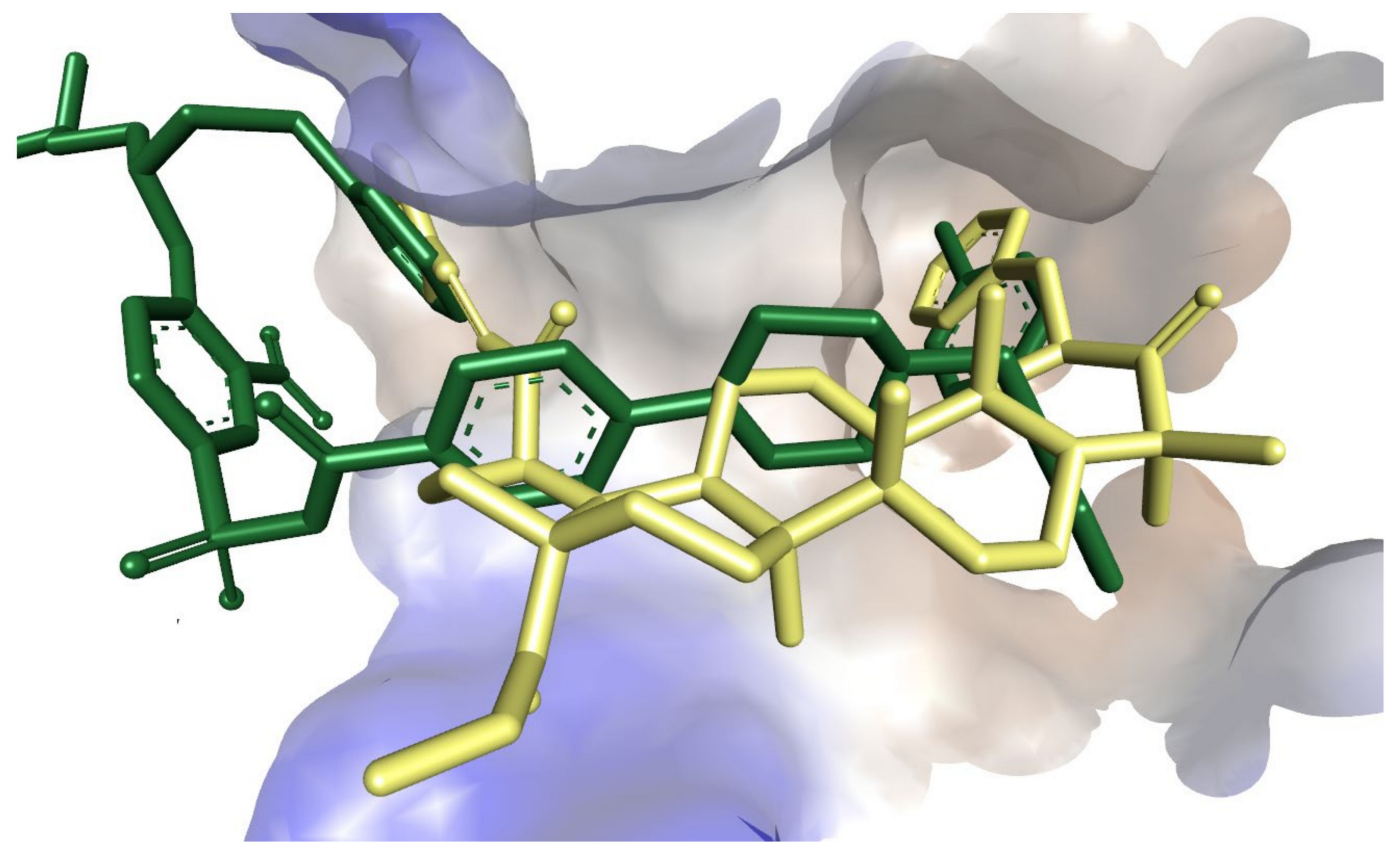
ABT-737 structure (green) overlayed on the docked conformation of compound **7** (yellow), within the Bcl-XL binding domain, revealing that both compounds occupy the same two hydrophobic pockets.

**Figure 13:**
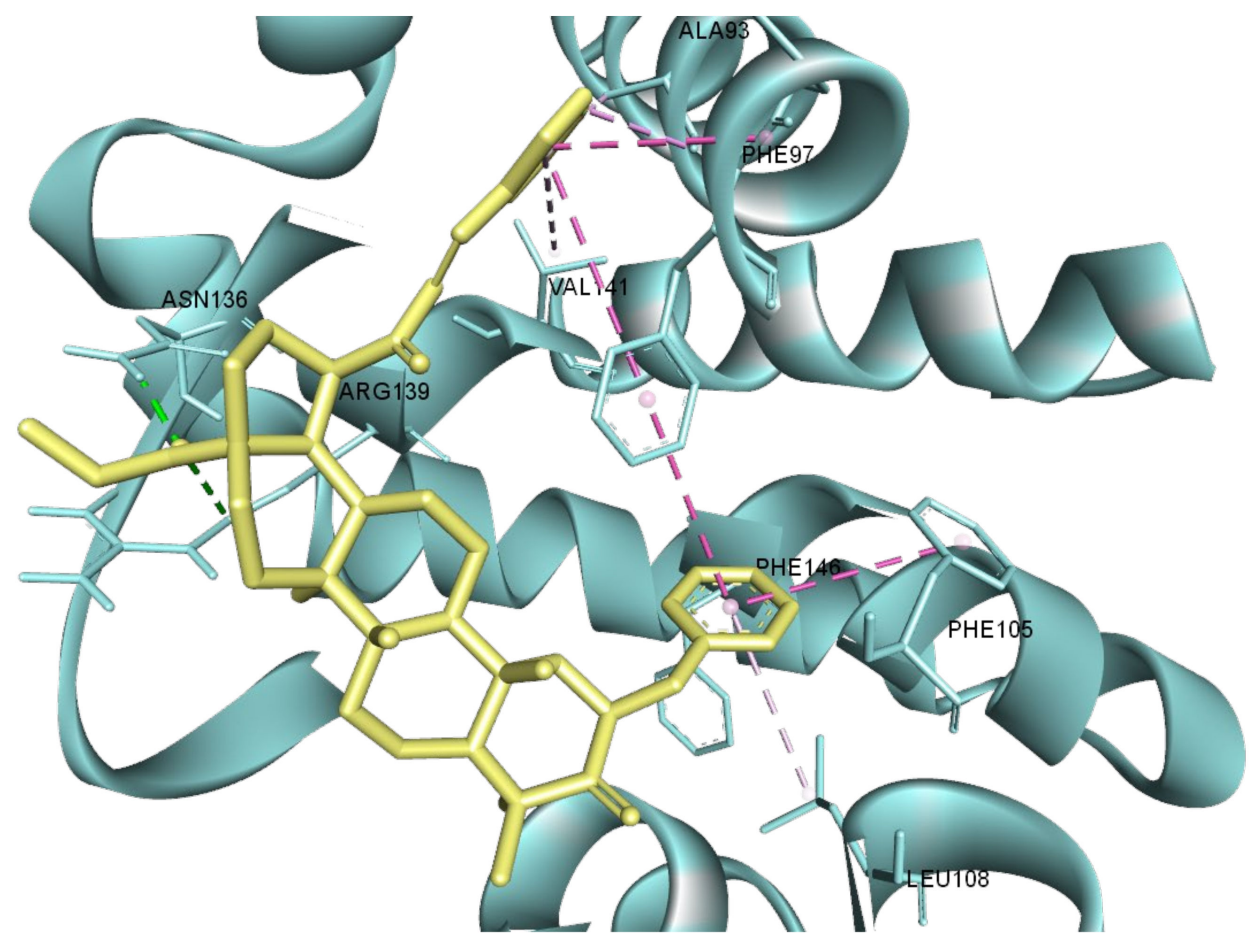
Structure of Bcl-XL (2YXJ) in complex with compound **7** (yellow); HB interactions are depicted as green dotted lines and hydrophobic interactions as purple dotted lines; interacting amino acids are shown as light blue sticks.

**Figure 14:**
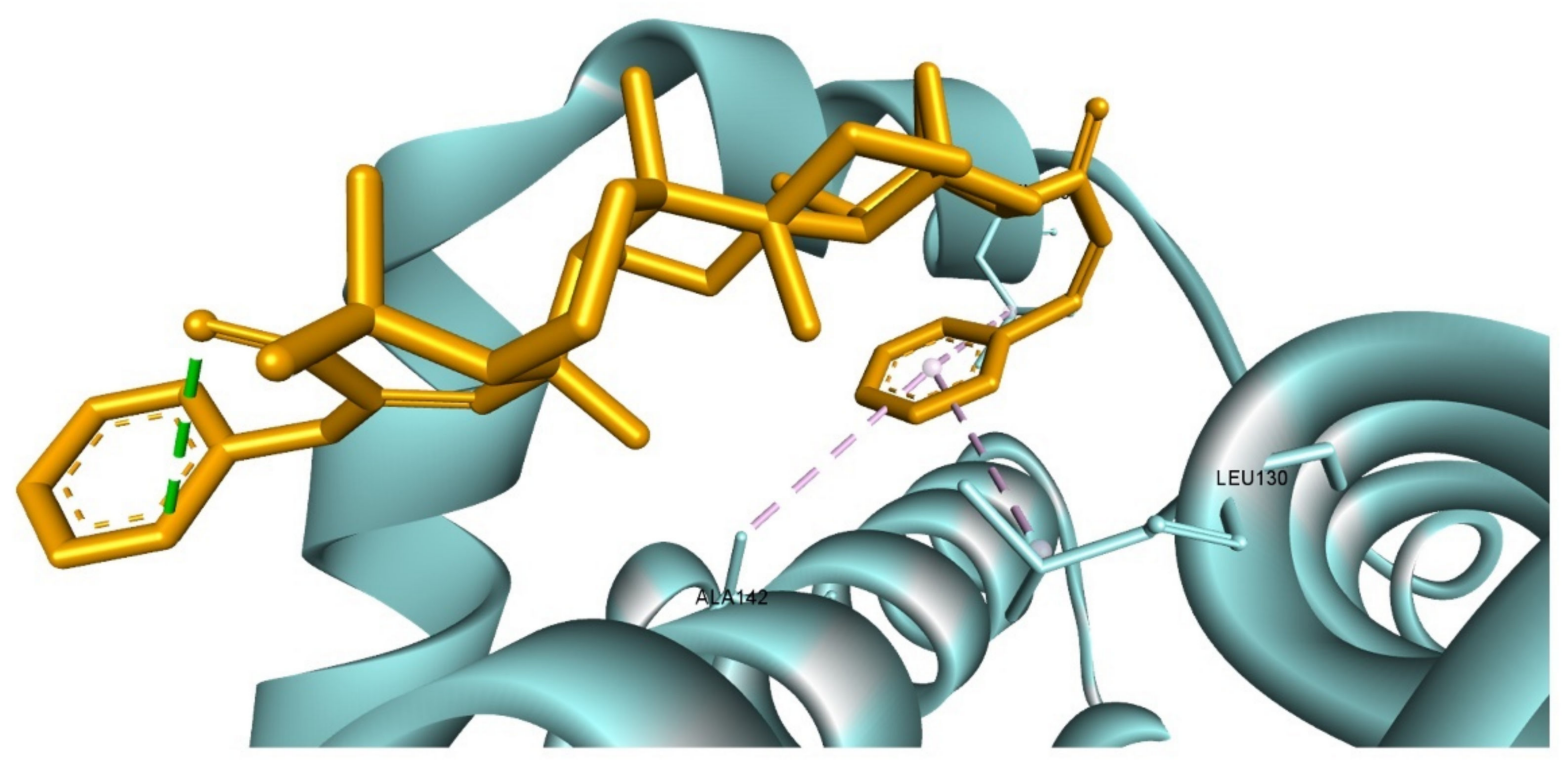
Structure of Bcl-XL (2YXJ) in complex with compound **6** (orange); HB interactions are depicted as green dotted lines and hydrophobic interactions as purple dotted lines; interacting amino acids are shown as light blue sticks.

Our results indicate that compounds **6** and **7** had excellent docking scores compared to those of the native ligand, in the case of the Bcl-XL protein (PDB ID: 2YXJ). Compound **6** had a close ∆G value to that of the control (−10.4 kcal/mol vs. −10.7 kcal/mol for the native ligand), while **7** scored higher compared to the co-crystallized ligand (-11.1 kcal/mol). Receptor-ligand interactions show that **7** has particular orientation within Bcl-XL binding site, similar to that of the native ligand (ABT-737) (Figure 12). Compound **7** interacts with two (p2 and p4) of the four Bcl-XL hydrophobic pockets [51]. The structure forms hydrophobic interactions through both pyridine rings with amino acids Ala93, Glu96, Phe97, and Val141 (1st pyridine ring); Phe105, Leu108, and Phe146 (2nd pyridine ring); and two supplementary hydrogen bonds (HBs) with Asn136 and Arg139 (Figure 13). On the other hand, compound **6** interacts with only one of the two hydrophobic pockets through a pyridine ring. The other pyridine moiety is “pulled away” from the hydrophobic pocket by an intermolecular HB, directing the ring towards the adjacent oxo group (Figure 14). Given the fact that **6** and **7** share a high structural similarity, this interaction pattern is the reason why 6 scored lower (higher ∆G value) compared to compound **7**.

According to our obtained results, compound **7** is a strong inhibitor candidate for Bcl-XL, revealing a higher docking score compared to that of ABT-737 (Bcl-XL inhibitor). ABT-737 was designed to interact with two (p2, p4) of the four hydrophobic pockets with which BH3-only proteins interact in the active site of Bcl-XL [51]. The docking score obtained by compound **7** is due to its structural similarity with ABT-737, having two aromatic rings at both extremities and a hydrophobic center and thus is able to interact in a highly similar fashion with the two hydrophobic pockets p2 and p4. Previous studies evaluating the in silico inhibitory effect of a set natural compounds against the antiapoptotic proteins Bcl-XL, Bcl-2, and Mcl-1 reported the high potential of triterpenes such as ursolic acid, oleanolic acid, or cycloartenol to act as inhibitors of these targets [52]. Furthermore, ursolic acid and oleanolic acid obtained higher docking scores against Bcl-XL compared to the Bcl-XL inhibitor ATB-737. Our results are in line with this reported data and suggest that compounds **6** and **7** may exhibit their proapoptotic-based anti-cancerous activity through both apoptotic gene regulation and antiapoptotic protein inhibition.

#### 2.2.6. Effect of Compounds **6** and **7** on the Normal and Tumor Angiogenesis Process by CAM Assay and Irritation Potential Determination Using the HET-CAM Assay

Angiogenesis is a physiological highly regulated process that consists in the formation of new blood and lymphatic vessels derived from the existing vasculature; in malignant tissues, angiogenesis delivers nutrients and oxygen to the tumor site, removes metabolic waste, and enables the metastatic process [53]. Malignant tissues display high angiogenic abilities since neovascularization is crucial for tumor growth beyond 1–2 mm; in the absence of angiogenesis the tumor shrinks and even dies out [54]. The antiangiogenic therapy in cancer stands therefore as a promising alternative to current options. However, despite the discovery and approval of some antiangiogenic agents, there are still limitations in clinical efficacy due to drug resistance, toxicity, the interventions of proangiogenic signaling pathways, and so on [55]. We investigated the antiangiogenic potential of the two compounds by means of CAM assay which was introduced in the early 20th century as an alternative to in vivo experiments due to ethical considerations [56]. The CAM assay reacts through an inflammatory response to external stimuli in a similar manner with mammalian models thus providing the possibility to extrapolate the results in terms of biocompatibility; in addition, due to native immunodeficiency, the CAM allows the growth of malignant xenografts [57], which makes it a valuable model for tumor development and testing of anticancer drugs. The procedure is simple, fast, relatively not expensive [58], and may provide the identification of early biological responses that might me overlooked in animal experiments [57]; in addition, the method allows treatment testing on patient-derived tumor models within the development of precision medicine [59].

**Figure 15:**
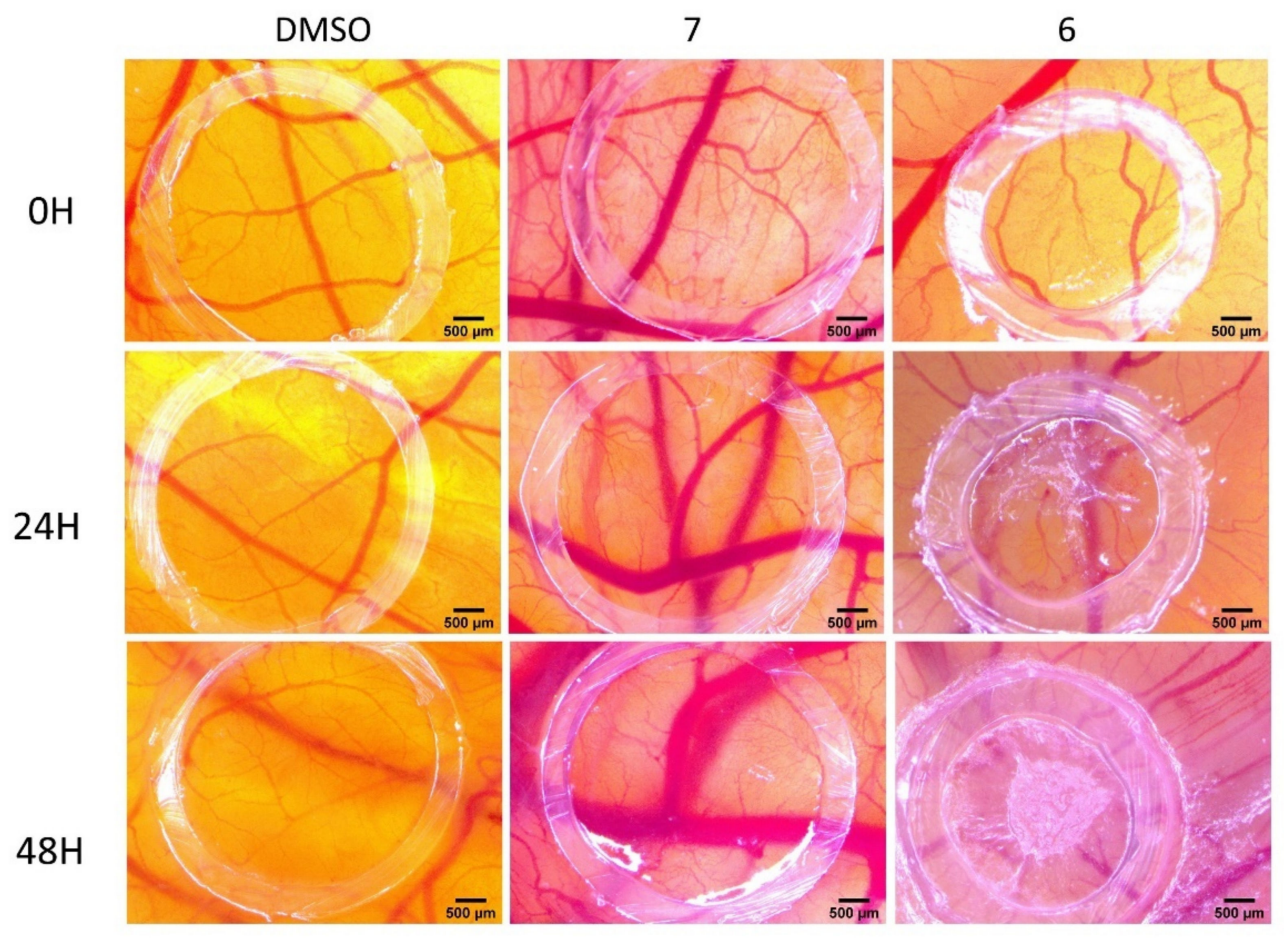
The angiogenesis assessment of compounds **6** and **7** using the CAM assay. Stereomicroscope images demonstrate daily modifications upon the treated vascular plexus; scale bars represent 500 µm.

Solutions (1 µM) of compounds **6** and **7** prepared in 0.5% aqueous DMSO, were applied on CAM in order to assess their antiangiogenic potential; the 0.5% DMSO in distilled water was used as control which did not induce the alteration of the normal angiogenic process (Figure 15). Compound **6** only slightly affected angiogenesis but within 48 h a vascular reaction could be observed, with numerous capillaries converging toward the ring; compound **7** did not interfere with the normal angiogenic process.

Our research group previously reported the antiangiogenic activity of the lupane scaffold betulinic acid [60]; contrary to these findings, the platanoate derivative **7**, although closely related to betulinic acid in terms of the main scaffold, does not influence the angiogenic process.

While ursolic acid, its structural relatives boswellic and asiatic acids, as well as some of their derivatives were revealed as effective antiangiogenic agents [61] by acting on various steps of the angiogenesis process, there are some authors that issue a warning in this regard. As an example, previous reports suggest that ursolic acid can stimulate certain key steps of angiogenesis and, although it is able to inhibit angiogenesis in CAM assay, the overall in vivo result might be controversial [62]. Such opposing effects were also reported in a later study, suggesting that the pro- or antiangiogenic activity of ursolic acid depends on its concentration and culture environment as well [63]. Ursane-type triterpenes are reportedly wound healing agents through stimulation of angiogenesis among other effects [64]; considering tumors as “wounds that never heal”, the final result of angiogenesis is determined by a competitive interaction between angiogenesis regulators with various affinities for tissue binding sites [65]. It is therefore presumable that ursane derivatives act by modulating the activity of such regulators and thus influence the angiogenic process in various ways generating conflicting results. Further studies should take the testing of ursane derivatives into consideration by using a wider range of concentrations. 

**Figure 16:**
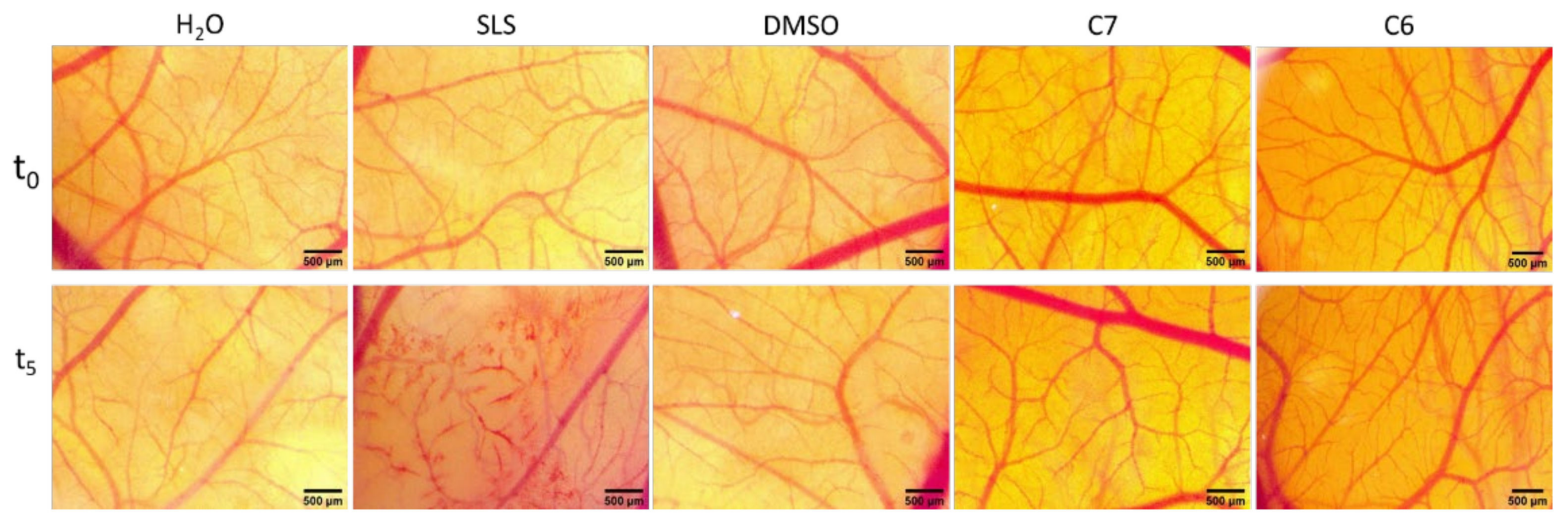
The irritation potential assessment using the HET-CAM method. Stereomicroscope images show the in face chorioallantoic membrane before (t0) and 300 s after application (t5) of 300 µL of the test sample in concentration of 1 µM and control samples (distilled water H_2_O as negative control, SLS 0.5% as positive control and DMSO 0.5% as solvent control); scale bars represent 500 µm.

HET-CAM assay was used in order to assess the toxicity of compounds **6** and **7** (Figure 16); the procedure is a low-cost, unique model in biomedical research [66] that allows the testing of the irritation potency of chemical compounds while simultaneously avoiding in vivo experiments [66]. Within this procedure, compounds are placed in direct contact with the CAM where they trigger similar responds as inflammatory reactions induced in conjunctive blood vessels: hemorrhage, lysis, and coagulation; therefore, the assay is able to predict the irritating potential of a compound [67]. In our study, the irritation potential was quantified on a scale from 0 to 21, with 0 indicating non-irritant compounds while 21 was the strongest irritant; sodium laurylsulphate (SLS) 0.5% and distilled water were used as strong irritant and non-irritant references, respectively. The Luepke scale was used to establish the irritation score: 0–0.9 indicates non-irritant, 1–4.9 indicates weakly irritant, 5–8.9 indicates moderately irritant, and 9–21 means strongly irritant [68,69]. During the investigation, none of the irritation indicators such as haemorrhage, lysis and coagulability were reported thus leading to the assignment of score 0 to the tested compounds. The positive control sodium laurylsulphate generated a score of 14.05 which qualifies it as a strong irritant while the solvent exhibited a mild irritation potential as shown by the score of 4.53. The non-irritant character of the two compounds revealed by the HET-CAM assay indicated their biocompatibility with mucosal tissues thus enabling their biomedical use (Table 6).

## 3. Materials and Methods

### 3.1. Experimental Part

#### 3.1.1. General 

The spectra were recorded at the Center for the Collective Use ‘Chemistry’ of the Ufa Institute of Chemistry of the Russian Academy of Sciences. ^1^H and ^13^C NMR spectra were recorded on a “Bruker AM-500” (Bruker, Billerica, MA, USA, 500 and 125.5 MHz respectively, δ, ppm, Hz) in CDCl_3_, internal standard—tetramethylsilane. Mass spectra were obtained on a liquid chromatograph–mass spectrometer LCMS-2010 EV (Shimadzu, Kyoto, Japan). Melting points were detected on a microtable «Rapido PHMK05» (Nagema, Dresden, Germany). Optical rotations were measured on a polarimeter Perkin-Elmer 241 MC (PerkinElmer, Waltham, MA, USA) in a tube length of 1 dm. Elemental analysis was performed on a Euro EA-3000 CHNS analyzer (Eurovector, Milan, Italy), the main standard is acetanilide. Thin-layer chromatography analyzes were performed on Sorbfil plates (Sorbpolimer, Krasnodar, Russia), using the solvent system chloroform–ethyl acetate, 40:1. Substances were detected by a 10% solution of sulfuric acid solution with subsequent heating at 100–120 °C for 2–3 min. All chemicals were of reagent grade (Sigma-Aldrich). Compounds 1 [17], 2 [20] and 3 [16] were obtained as previously reported.

#### 3.1.2. Synthesis of Compounds **4**–**8**

To a solution of compound **1**, **2**, or **3** (1 mmol) in ethanol (5 mL) under stirring, furfural (0.13 g, 1.3 mmol) or 3-pyridine carboxaldehyde (0.14 g, 1.3 mmol) (corresponding to 1 eqv. or 2 eqv.) and 40% KOH in ethanol (2.5 mL) were added. The mixture was stirred for 24 h at room temperature, pH was adjusted to neutral by adding an aqueous solution of 5% HCl, and the mixture was poured into cold water (50 mL). The residue was filtered off, washed with water, and dried, then purified by column chromatography on Al_2_O_3_ using petroleum ether—CHCl_3_ (2:1 to 1:3) as eluent.

##### 3β-Hydroxy-21-[3-(2E-pyridinyl)-prop-2-en-1-one]-20β,28-epoxy-18α,19βH-ursane **4**

Beige solid; yield (84%); m.p.: 135 °C; [α]^20^_D_ +23 (c 0.10, CHCl_3_). ^13^C NMR (δ, ppm, CDCl_3_, 125.5 MHz): 201.13 (C31); 150.61 (C5′); 149.65 (C3′); 138.18 (C1′); 134.80 (C7′); 130.78 (C2′); 127.69 (C32); 123.79 (C6′); 78.85 (C3); 73.39 (C20); 68.19 (C28); 55.43 (C5); 50.69 (C9); 48.95 (C21); 46.31 (C18); 44.38 (C19); 41.27 (C14); 40.71 (C8); 39.88 (C13); 38.88 (C1); 38.88 (C4); 37.18 (C10); 37.18 (C22); 33.94 (C7); 31.15 (C17); 29.78 (C16); 28.00 (C23); 27.40 (C2); 26.43 (C15); 25.83 (C12); 23.45 (C30); 21.24 (C11); 19.85 (C29); 18.24 (C6); 16.38 (C25); 15.79 (C26); 15.41 (C24); 14.24 (C27). ^1^H NMR (δ, ppm, CDCl_3_, 500 MHz): 8.79 (d, 1H, ^4^J_3′-7′_ = 2.1, H-3′); 8.60 (dd, 1H, ^3^J_5′-6′_ = 4.8, ^4^J_5′-7′_ = 1.8, H-5′); 7.89 (dd, 1H, ^3^J_7′-6′_ = 7.8, ^4^J_7′-3′_ = 2.1, ^4^J_7′-5′_ = 1.8, H-7′); 7.59 (d, 1H, ^3^J_1′-32_ = 15.9, H-1′); 7.35 (dd, 1H, ^3^J_6′-7′_ = 7.8, ^3^J_6′-5′_ = 4.8, H-6′); 7.06 (d, 1H, ^3^J_32-1′_ = 15.9, H-32); 4.22 (dd, 1H, ^2^J = 8.5, ^4^J_28b-22α_ = 2.9, Hb-28); 3.55 (dd, 1H, ^2^J = 8.5, ^4^J_28a-18_ = 1.2, Ha-28); 3.21 (dd, 1H, ^3^J_3-2ax_ = 11.2, ^3^J_3-2eq_ = 4.7, H-3); 3.14 (dd, 1H, ^3^J_21-22α_ = 11.3, ^3^J_21-22β_ = 5.0, H-21); 1.79 (dd, 1H, ^2^J = 13.4, ^3^J_22β-21_ = 5.0, Hβ-22); 1.73 (m, 1H, Heq-12); 1.73 (m, 1H, Heq-1); 1.72 (m, 1H, H-13); 1.64 (m, 1H, Heq-2); 1.62 (m, 1H, Hax-2); 1.57 (m, 1H, Heq-11); 1.54 (m, 1H, Heq-6); 1.50 (td, 1H, ^2^J = 13.9, ^3^J_15ax-16ax_ = 13.9, ^3^J_15ax-16eq_ = 3.7, Hax-15); 1.47 (m, 1H, Hα-22); 1.45 (m, 1H, H-19); 1.40 (m, 1H, Hax-6); 1.39 (m, 1H, Hax-7); 1.39 (m, 1H, Heq-7); 1.38 (m, 1H, Hax-16); 1.35 (m, 1H, H-9); 1.31 (qd, 1H, ^2^J = 12.8, ^3^J_11ax-12ax_ = 12.8, ^3^J_11ax-9_ = 12.8, ^3^J_11ax-12eq_ = 4.0, Hax-11); 1.21 (ddd, 1H, ^2^J = 13.9, ^3^J_16eq-15ax_ = 3.7, ^3^J_16eq-15eq_ = 3.0, Heq-16); 1.10 (s, 3H, H_3_-30); 1.10 (m, 1H, Hax-12); 1.05 (ddd, 1H, ^2^J = 13.9, ^3^J_15eq-16ax_ = 3.6, ^3^J_15eq-16eq_ = 3.0, Heq-15); 1.00 (s, 3H, H_3_-26); 1.00 (d, 3H, ^3^J_29-19_ = 6.7, H_3_-29); 0.98 (s, 3H, H_3_-23); 0.96 (td, 1H, ^2^J = 13.0, ^3^J_1ax-2ax_ = 13.0, ^3^J_1ax-2eq_ = 4.7, Hax-1); 0.93 (s, 3H, H_3_-27); 0.93 (m, 1H, H-18); 0.86 (s, 3H, H_3_-25); 0.77 (s, 3H, H_3_-24); 0.70 (dd, 1H, ^3^J_5-6ax_ = 10.0, ^3^J_5-6eq_ = 3.2, H-5). Anal. calcd for C_38_H_55_NO_3_(573.85): C, 79.53; H, 9.66; N, 2.44. Found: C, 79.50; H, 9.64; N, 2.45. MS(APCI) *m*/*z* 574.84 [M+ H]^+^.

##### 3β-Hydroxy-21-[3-(2E-furyl)-prop-2-en-1-one]-20β,28-epoxy-18α,19βH-ursane **5**

Beige solid; yield (85%); m.p.: 152 °C; [α]^20^_D_ +70 (c 0.10, CHCl_3_).^13^C NMR (δ, ppm, CDCl_3_, 125.5 MHz): 201.47 (C31); 151.49 (C2′); 144.65 (C4′); 128.22 (C1′); 123.47 (C32); 115.83 (C6′); 112.52 (C5′); 78.93 (C3); 73.46 (C20); 69.00 (C28); 55.41 (C5); 50.69 (C9); 48.86 (C21); 46.44 (C18); 44.38 (C19); 41.27 (C14); 40.70 (C8); 39.79 (C13); 38.87 (C1); 38.85 (C4); 37.21 (C10); 37.18 (C22); 33.93 (C7); 31.13 (C17); 29.74 (C16); 27.98 (C23); 27.37 (C2); 26.43 (C15); 25.79 (C12); 23.48 (C30); 21.24 (C11); 19.91 (C29); 18.23 (C6); 16.38 (C25); 15.78 (C26); 15.39 (C24); 14.26 (C27). ^1^H NMR (δ, ppm, CDCl_3_, 500 MHz): 7.47 (d, 1H, ^3^J_4′-5′_ = 1.5, H-4′); 7.35 (d, 1H, ^3^J_1′-32_ = 15.5, H-1′); 6.82 (d, 1H, ^3^J_32-1′_ = 15.5, H-32); 6.64 (d, 1H, ^3^J_6′-5′_ = 3.6, H-2′); 6.47 (dd, 1H, ^3^J_5′-6′_ = 3.6, ^3^J_5′-4′_ = 1.5, H-5′); 4.17 (dd, 1H, ^2^J = 8.6, ^4^J_28b-22α_ = 3.2, Hb-28); 3.55 (d, 1H, ^2^J = 8.6, Ha-28); 3.19 (dd, 1H, ^3^J_3-2ax_ = 11.3, ^3^J_3-2eq_ = 5.3, H-3); 3.09 (dd, 1H, ^3^J_21-22α_ = 11.1, ^3^J_21-22β_ = 4.8, H-21); 1.80 (dd, 1H, ^2^J = 13.4, ^3^J_22β-21_ = 4.8, Hβ-22); 1.71 (m, 1H, Heq-12); 1.71 (m, 1H, Heq-1); 1.70 (m, 1H, H-13); 1.62 (m, 1H, Heq-2); 1.60 (m, 1H, Hax-2); 1.55 (dq, 1H, ^2^J = 12.9, ^3^J_11eq-12ax_ = 3.5, ^3^J_11eq-12eq_ = 3.5, ^3^J_11eq-9_ = 3.5, Heq-11); 1.52 (m, 1H, Heq-6); 1.48 (m, 1H, Hax-15); 1.47 (m, 1H, Hα-22); 1.43 (m, 1H, H-19); 1.38 (m, 1H, Hax-6); 1.37 (m, 1H, Hax-7); 1.37 (m, 1H, Heq-7); 1.36 (m, 1H, Hax-16); 1.33 (m, 1H, H-9); 1.29 (qd, 1H, ^2^J = 12.9, ^3^J_11ax-12ax_ = 12.9, ^3^J_11ax-9_ = 12.9, ^3^J_11ax-12eq_ = 4.1, Hax-11); 1.19 (ddd, 1H, ^2^J = 13.9, ^3^J_16eq-15ax_ = 4.2, ^3^J_16eq-15eq_ = 2.8, Heq-16); 1.08 (s, 3H, H_3_-30); 1.08 (m, 1H, Hax-12); 1.03 (m, 1H, Heq-15); 0.98 (s, 3H, H_3_-26); 0.97 (d, 3H, ^3^J_29-19_ = 6.8, H_3_-29); 0.96 (s, 3H, H_3_-23); 0.94 (m, 1H, Hax-1); 0.91 (s, 3H, H_3_-27); 0.91 (m, 1H, H-18); 0.84 (s, 3H, H_3_-25); 0.76 (s, 3H, H_3_-24); 0.68 (dd, 1H, ^3^J_5-6ax_ = 10.8, ^3^J_5-6eq_ = 3.6, H-5). Anal. calcd for C_37_H_54_O_4_(562.82): C, 78.96; H, 9.67. Found: C, 78.95; H, 9.70. MS(APCI) *m*/*z* 563.81 [M+ H]^+^.

##### 3-Oxo-2-(3-pyridinylidene)-21-[3-(2E-pyridinyl)-prop-2-en-1-one]-20β,28-epoxy-18α,19βH-ursane **6**

Brown solid; yield (80%); m.p.: 95 °C; [α]^20^_D_ = +60 (c 0.10, CHCl_3_).^13^C NMR (δ, ppm, CDCl_3_, 125.5 MHz): 207.60 (C3); 201.10 (C31); 150.91 (C5′); 150.82 (C3″); 149.91 (C3′); 148.99 (C5″); 138.36 (C1′); 137.23 (C7″); 136.38 (C2); 134.60 (C7′); 133.46 (C1″); 131.90 (C2″); 130.66 (C2′); 127.62 (C32); 123.72 (C6′); 123.40 (C6″); 73.41 (C20); 68.15 (C28); 52.93 (C5); 48.88 (C21); 48.61 (C9); 46.28 (C18); 45.28 (C4); 44.67 (C1); 44.35 (C19); 41.38 (C14); 40.55 (C8); 39.93 (C13); 37.18 (C22); 36.53 (C10); 32.70 (C7); 31.16 (C17); 29.70 (C16); 29.41 (C23); 26.40 (C15); 25.94 (C12); 23.46 (C30); 22.34 (C24); 22.05 (C11); 20.26 (C6); 19.82 (C29); 16.05 (C25); 15.33 (C26);14.16 (C27). ^1^H NMR (δ, ppm, CDCl_3_, 500 MHz): 8.79 (d, 1H, ^4^J_3′-7′_ = 2.2, H-3′); 8.73 (d, 1H, ^4^J_3″-7″_ = 2.3, H-3″); 8.60 (dd, 1H, ^3^J_5′-6′_ = 4.8, ^4^J_5′-7′_ = 1.7, H-5′); 8.55 (dd, 1H, ^3^J_5″-6″_ = 4.8, ^4^J_5″-7″_ = 1.8, H-5″); 7.88 (dd, 1H, ^3^J_7′-6′_ = 7.9, ^4^J_7′-3′_ = 2.2, ^4^J_7′-5′_ = 1.7, H-7′); 7.73 (dd, 1H, ^3^J_7″-6″_ = 7.8, ^4^J_7″-3″_ = 2.3, ^4^J_7″-5″_ = 1.8, H-7″); 7.60 (d, 1H, ^3^J_1′-32_ = 16.0, H-1′); 7.44 (m, 1H, H-1″); 7.36 (dd, 1H, ^3^J_6″-7″_ = 7.8, ^3^J_6″-5″_ = 4.8, H-6″); 7.34 (dd, 1H, ^3^J_6′-7′_ = 7.9, ^3^J_6′-5′_ = 4.8, H-6′); 7.06 (d, 1H, ^3^J_32-1′_ = 16.0, H-32); 4.22 (dd, 1H, ^2^J = 8.8, ^4^J_28b-22α_ = 2.9, Hb-28); 3.57 (dd, 1H, ^2^J = 8.8, ^4^J_28a-18_ = 1.2, Ha-28); 3.17 (dd, 1H, ^3^J_21-22α_ = 11.1, ^3^J_21-22β_ = 5.2, H-21); 3.07 (dd, 1H, ^2^J = 16.5, ^4^J_1b-1″_ = 1.5, Hb-1); 2.30 (dd, 1H, ^2^J = 16.5, ^4^J_1a-1″_ = 2.7, Ha-1); 1.81 (dd, 1H, ^2^J = 13.5, ^3^J_22β-21_ = 5.2, Hβ-22); 1.78 (m, 1H, Heq-12); 1.76 (ddd, 1H, ^3^J_13-12ax_ = 12.6, ^3^J_13-18_ = 10.8, ^3^J_13-12eq_ = 3.4, H-13); 1.63 (ddt, 1H, ^2^J = 13.0, ^3^J_11eq-12ax_ = 4.4, ^3^J_11eq-12eq_ = 2.8, ^3^J_11eq-9_ = 2.8, Heq-11); 1.57 (dd, 1H, ^3^J_9-11ax_ = 12.1, ^3^J_9-11eq_ = 2.7, H-9); 1.51 (m, 1H, H-5); 1.51 (m, 1H, Hax-6); 1.51 (m, 1H, Heq-6); 1.50 (m, 1H, Hax-15); 1.48 (m, 1H, Hα-22); 1.47 (m, 1H, H-19); 1.46 (m, 1H, Hax-7); 1.46 (m, 1H, Heq-7); 1.40 (td, 1H, ^2^J = 13.7, ^3^J_16ax-15ax_ = 13.7, ^3^J_16ax-15eq_ = 4.4, Hax-16); 1.33 (m, 1H, Hax-11); 1.25 (m, 1H, Heq-16); 1.18 (s, 3H, H_3_-24); 1.17 (m, 1H, Hax-12); 1.15 (s, 3H, H_3_-23); 1.12 (m, 1H, Heq-15); 1.11 (s, 3H, H_3_-30); 1.05 (s, 3H, H_3_-26); 1.02 (d, 3H, ^3^J_29-19_ = 7.1, H_3_-29); 0.98 (m, 1H, H-18); 0.97 (s, 3H, H_3_-27); 0.84 (s, 3H, H_3_-25). Anal. calcd for C_44_H_58_N_2_O_3_ (662.94): C, 79.72; H, 8.82; N, 4.23. Found: C, 79.70; H, 8.85; N, 4.20. MS(APCI) *m*/*z* 663.94 [M+ H]^+^.

##### Methyl 3,20-dioxo-2-(3-pyridinylidene)-29-nor-lup-28-oate **7**

Beige solid; yield (82%);m.p.: 135 °C; [α]^20^_D_ +27 (c 0.10, CHCl_3_). ^13^C NMR (δ, ppm, CDCl_3_, 125.5 MHz): (δ, ppm, CDCl_3_, 125.5 MHz): 207.60 (C3); 203.37 (C20); 176.58 (C28); 151.28 (C3″); 151.03 (C5′); 150.03 (C3′); 148.99 (C5″); 138.99 (C1′); 136.73 (C7″); 136.41 (C2); 134.45 (C7′); 133.31 (C1″); 131.78 (C2″); 130.48 (C2′); 128.41 (C30); 123.78 (C6′); 123.32 (C6″); 56.42 (C17); 52.79 (C5); 51.56 (C31); 50.12 (C18); 48.61 (C19); 48.22 (C9); 45.23 (C4); 44.59 (C1); 42.41 (C14); 40.44 (C8); 37.59 (C13); 36.88 (C22); 36.48 (C10); 33.01 (C7); 31.42 (C16); 29.71 (C15); 29.42 (C23); 29.08 (C21); 27.86 (C12); 22.33 (C24); 21.78 (C11); 20.31 (C6); 15.79 (C25); 15.33 (C26); 14.69 (C27). ^1^H NMR (δ, ppm, CDCl_3_, 500 MHz): 8.80 (d, 1H, ^4^J_3′-7′_ = 2.2, H-3′); 8.64 (d, 1H, ^4^J_3″-7″_ = 2.1, H-3″); 8.62 (dd, 1H, ^3^J_5′-6′_ = 4.9, ^4^J_5′-7′_ = 1.9, H-5′); 8.51 (dd, 1H, ^3^J_5″-6″_ = 4.9, ^4^J_5″-7″_ = 1.8, H-5″); 7.90 (dd, 1H, ^3^J_7′-6′_ = 8.0, ^4^J_7′-3′_ = 2.2, ^4^J_7′-5′_ = 1.9, H-7′); 7.69 (dd, 1H, ^3^J_7″-6″_ = 8.0, ^4^J_7″-3″_ = 2.1, ^4^J_7″-5″_ = 1.8, H-7″); 7.59 (d, 1H, ^3^J_1′-30_ = 16.3, H-1′); 7.41 (m, 1H, H-1′); 7.35 (dd, 1H, ^3^J_6′-7′_ = 8.0, ^3^J_6′-5′_ = 4.9, H-6′); 7.32 (dd, 1H, ^3^J_6″-7″_ = 8.0, ^3^J_6″-5″_ = 4.9, H-6″); 6.85 (d, 1H, ^3^J_30-1′_ = 16.3, H-30); 3.72 (s, 3H, H_3_-31); 3.65 (td, 1H, ^3^J_19-18_ = 11.3, ^3^J_19-20ax_ = 11.3, ^3^J_19-20eq_ = 4.8, H-19); 2.97 (dd, 1H, ^2^J = 16.5, ^4^J_1b-1″_ = 1.2, Hb-1); 2.34 (t, 1H, ^3^J_18-13_ = 11.3, ^3^J_18-19_ = 11.3, H-18); 2.32 (m, 1H, Heq-16); 2.22 (dd, 1H, ^2^J = 16.5, ^4^J_1a-1″_ = 3.1, Ha-1); 2.13 (ddd, 1H, ^3^J_13-12ax_ = 12.6, ^3^J_13-18_ = 11.3, ^3^J_13-12eq_ = 3.4, H-13); 2.10 (dddd, 1H, ^2^J = 13.3, ^3^J_21ax-19_ = 11.3, ^3^J_21ax-22ax_ = 9.8, ^3^J_21ax-22eq_ = 7.7, Hax-21); 1.97 (ddd, 1H, ^2^J = 12.4, ^3^J_22eq-21ax_ = 9.8, ^3^J_22eq-21eq_ = 1.9, Heq-22); 1.65 (ddd, 1H, ^2^J = 12.4, ^3^J_22ax-21ax_ = 9.8, ^3^J_22ax-21eq_ = 7.7, Hax-22); 1.60 (dddd, 1H, ^2^J = 13.3, ^3^J_21eq-22ax_ = 9.8, ^3^J_21eq-19_ = 4.8, ^3^J_21eq-22eq_ = 1.9, Heq-21); 1.52 (td, 1H, ^2^J = 13.2, ^3^J_16ax-15ax_ = 13.2, ^3^J_16ax-15eq_ = 3.6, Hax-16); 1.51 (m, 1H, H-9); 1.49 (m, 1H, Hax-6); 1.49 (m, 1H, Heq-6); 1.48 (m, 1H, H-5); 1.48 (m, 1H, Heq-11); 1.46 (m, 1H, Hax-7); 1.46 (m, 1H, Heq-7); 1.39 (td, 1H, ^2^J = 13.2, ^3^J_15ax-16ax_ = 13.2, ^3^J_15ax-16eq_ = 3.8, Hax-15); 1.27 (m, 1H, Heq-15); 1.27 (m, 1H, Hax-11); 1.16 (s, 3H, H_3_-24); 1.13 (s, 3H, H_3_-23); 1.13 (m, 1H, Hax-12); 1.13 (m, 1H, Heq-12); 1.07 (s, 3H, H_3_-27); 0.96 (s, 3H, H_3_-26); 0.77 (s, 3H, H_3_-25). Anal. calcd for C_42_H_52_N_4_O_4_ (648.89): C, 77.74; H, 8.08; N, 4.32. Found: C, 77.87; H, 8.15; N, 4.25. MS(APCI) *m*/*z* 649.40 [M+ H]^+^.

##### Methyl 3,20-dioxo-2,30-di-(3-pyridinylidene)-29-nor-lup-28-oate **8**

Beige solid; yield (80%); m.p.: 101 °C; [α]^20^_D_ +211 (c 0.10, CHCl_3_). ^13^C NMR (δ, ppm, CDCl_3_, 125.5 MHz): 212.31 (C20); 207.63 (C3); 176.46 (C28); 151.55 (C3′); 149.09 (C5′); 136.55 (C7′); 136.41 (C2); 133.33 (C1′); 131.76 (C2′); 123.30 (C6′); 56.33 (C17); 52.76 (C5); 51.49 (C31); 51.12 (C19); 49.16 (C18); 48.38 (C9); 45.22 (C4); 44.61 (C1); 42.30 (C14); 40.41 (C8); 37.41 (C13); 36.58 (C10); 36.45 (C22); 32.99 (C7); 31.37 (C16); 30.23 (C30); 29.65 (C15); 29.43 (C23); 28.21 (C21); 27.47 (C12); 22.33 (C24); 21.73 (C11); 20.30 (C6); 15.80 (C25); 15.32 (C26); 14.66 (C27). ^1^H NMR (δ, ppm, CDCl_3_, 500 MHz): 8.65 (d, 1H, ^4^J_3′-7′_ = 2.2, H-3′); 8.53 (dd, 1H, ^3^J_5′-6′_ = 4.8, ^4^J_5′-7′_ = 1.8, H-5′); 7.70 (dd, 1H, ^3^J_7′-6′_ = 7.9, ^4^J_7′-3′_ = 2.2, ^4^J_7′-5′_ = 1.8, H-7′); 7.41 (dd, 1H, ^4^J_1′-1a_ = 2.8, ^4^J_1′-1b_ = 1.5, H-1′); 7.34 (dd, 1H, ^3^J_6′-7′_ = 7.9, ^3^J_6′-5′_ = 4.8, H-6′); 3.66 (s, 3H, H_3_-31); 3.26 (td, 1H, ^3^J_19-18_ = 11.3, ^3^J_19-20ax_ = 11.3, ^3^J_19-20eq_ = 4.7, H-19); 2.98 (dd, 1H, ^2^J = 16.3, ^4^J_1b-1′_ = 1.5, Hb-1); 2.26 (dd, 1H, ^2^J = 16.3, ^4^J_1a-1′_ = 2.8, Ha-1); 2.26 (ddd, 1H, ^2^J =13.2, ^3^J_16eq-15ax_ = 4.2, ^3^J_16eq-15eq_ = 3.2, Heq-16); 2.18 (s, 1H, H_3_-30); 2.15 (t, 1H, ^3^J_18-13_ = 11.3, ^3^J_18-19_ = 11.3, H-18); 2.04 (ddd, 1H, ^3^J_13-12ax_ = 13.4, ^3^J_13-18_ = 11.3, ^3^J_13-12eq_ = 5.0, H-13); 2.04 (ddt, 1H, ^2^J = 13.2, ^3^J_20ax-19_ = 11.3, ^3^J_20ax-21ax_ = 7.1, ^3^J_20ax-21eq_ =7.1, Hax-21); 1.90 (ddd, 1H, ^2^J = 12.6, ^3^J_21eq-20ax_ = 7.1, ^3^J_21eq-20eq_ = 3.2, Heq-22); 1.54 (m, 1H, Hax-22); 1.52 (m, 1H, H-9); 1.51 (m, 1H, Heq-21); 1.50 (m, 1H, Hax-6); 1.50 (m, 1H, Heq-6); 1.49 (m, 1H, H-5); 1.49 (m, 1H, Heq-11); 1.48 (m, 1H, Hax-16); 1.46 (m, 1H, Hax-7); 1.46 (m, 1H, Heq-7); 1.45 (td, 1H, ^2^J = 12.6, ^3^J_15ax-16ax_ = 12.6, ^3^J_15ax-16eq_ = 4.2, Hax-15); 1.31 (qd, 1H, ^2^J = 12.0, ^3^J_11ax-12ax_ = 12.0, ^3^J_11ax-9_ = 12.0, ^3^J_11ax-12eq_ = 4.3, Hax-11); 1.22 (m, 1H, Heq-15); 1.15 (m, 1H, Hax-12); 1.14 (s, 3H, H_3_-24); 1.11 (s, 3H, H_3_-23); 1.06 (m, 1H, Heq-12); 1.03 (s, 3H, H_3_-27); 0.92 (s, 3H, H_3_-26); 0.77 (s, 3H, H_3_-25). Anal. calcd for C_36_H_49_NO_4_ (559.79): C, 77.24; H, 8.82; N, 2.50. Found: C, 77.35; H, 8.74; N, 2.44. MS(APCI) *m*/*z* 560.37 [M+ H]^+^.

### 3.2. NCI-60 Screening

Compounds **1**, **2**, and **4**–**8** were tested at one dose assay (10^−5^ M) toward a panel of approximately sixty cancer cell lines representing different cancer types: leukemia, melanoma, lung, colon, CNS, ovarian, renal, prostate, and breast cancers. Primary anticancer assays were performed according to the NCI protocol as described elsewhere (see, e.g., http://dtp.nci.nih.gov accessed on 16 October 2019) [70,71,72,73]. The compounds were added at a single concentration and the cell cultures were incubated for 48 h. The end point determinations were made with a protein binding dye, sulforhodamine B (SRB). The results for each compound are reported as the percent growth (GP %) of treated cells compared to untreated control cells (negative numbers indicate cell kill). Compounds with considerable activity against all tested human tumor cell lines (**6** and **7**) were selected for the advanced assay against a panel of approximately sixty tumor cell lines at 10-fold dilutions of five concentrations (100 µM, 10 µM, 1 µM, 0.1 µM, and 0.01 µM) [70,71,72,73]. The percentage of growth was evaluated spectrophotometrically versus controls not treated with test agents after 48-h exposure and using SRB protein assay to estimate cell viability or growth. Three antitumor activity dose–response parameters were calculated for each cell line: GI_50—_molar concentration of the compound that inhibits 50% net cell growth; TGI—molar concentration of the compound leading to the total inhibition; and LC_50_—molar concentration of the compound leading to 50% net cell death (presented in negative logarithm). Furthermore, mean graph midpoints (MG_MID) were calculated for each of the parameters, giving an average activity parameter over all cell lines for the tested compound. For the MG_MID calculation, insensitive cell lines were included with the highest concentration tested.

### 3.3. Cell Culture

Human melanoma cell lines A375, RPMI, and SK-MEL-28; human keratinocytes; and HaCaT were purchased from the American Type Culture Collection (ATCC) and human skin fibroblast, 1BR3 was obtained from the European Collection of Authenticated Cell Cultures (ECACC). A375 and HaCaT were cultured in Dulbecco’s Modified Eagle’s Medium (DMEM, Sigma-Aldrich) high-glucose medium supplemented with 10% fetal bovine serum (FBS, Gibco, ThermoFisher Scientific Waltham, MA, USA) and 1% penicillin/Strep, 10,000 IU/mL, Sigma-Aldrich, St. Louis, MO, USA) and RPMI, SK-MEL-28 and 1BR3 were cultured in Eagle’s Minimum Essential Medium (EMEM, Sigma-Aldrich, St. Louis, MO, USA) high-glucose medium supplemented with 10% fetal bovine serum (FBS, Gibco, ThermoFisher Scientific, Waltham, MA, USA) and 1% penicillin/Strep, 10,000 IU/mL, Sigma-Aldrich, St. Louis, MO, USA). The cells were incubated under standard temperature conditions of 37 °C and humidity containing 5% CO_2_.

### 3.4. Cytotoxicity Assay for Healthy Human Cells

The in vitro cytotoxicity was determined by means of (4,5-dimethylthiazol-2-yl)-2,5-diphenyltetrazolium bromide (MTT) method. In brief, HaCaT, 1BR3 cells were seeded in 96-well plates at an initial density of 1104 cells/well and allowed to attach. Next, the old medium was removed and a fresh one was added containing five concentrations (100 µM, 10 µM, 1 µM, 0.1 µM, and 0.01 µM) of the tested compounds **6** and **7** after which cells were incubated for 48 h. The control cells were treated with the same amount of DMSO, the highest concentration of DMSO present in the medium being 0.5%. A volume of 10 mL MTT reagent (5 mg/mL) was added in each well. During a 4 h contact period, the intact mitochondrial reductase converted and precipitated MTT as blue crystals. The precipitated crystals were dissolved in 100 mL of lysis solution provided by the manufacturer (Sigma-Aldrich). Finally, the reduced MTT was spectrophotometrically analyzed at 570 nm, using a microplate reader (xMark Microplate Spectrophotometer, Bio-Rad, Hercules, CA, USA). GI_50_ was calculated for each healthy cell line.

### 3.5. DAPI Assay

The DAPI assay was performed on three human melanoma cell lines: A375, RPMI, and SK-MEL-28. The cells were cultured in 24-well plates at 2x10^5^ cells/well; compounds **6** and **7** in three different concentrations (0.1, 1, and 5 μM); and three DMSO concentrations (0.1, 1, and 5 μM) were used to stimulate cells. The immunofluorescent staining technique was performed according to a previously described protocol [74] and adapted to our laboratory conditions. Thus, after 24 h of stimulation, the cells were fixed with 4% paraformaldehyde and incubated at room temperature for 1 h. Cell permeability was 30 min later performed with 2% Triton X solution in phosphate-buffered saline (PBS). The 300 nM 4’,6-diamidino-2-phenylindole (DAPI) staining was used to visualize cell nuclei under the fluorescence microscope at 40x magnification using CellSens V1.15 software (Olympus, Tokyo, Japan) and Image J software. 

### 3.6. The Chorioallantoic Membrane Assay

The potential effects of compounds **6** and **7** on the angiogenesis process were investigated using the chorioallantoic membrane (CAM) assay which uses fertilized chicken eggs, incubated at 37 °C, under controlled humidity. On the third day of incubation (embryonic day 3 of development, EDD 3), a small opening was made at one end of the eggs and 5–6 mL of albumen was removed, thus facilitating the separation of the chorioallantoic membrane. On EDD 4, a window was cut on the upper side of the egg and subsequently resealed, returning the specimens to the incubator. On EDD 7 (0 h), 10 µL of each test solution was administered inside a plastic ring previously applied on the intensely vascularized surface of the CAM [75,76]. Compounds were tested in concentration of 1 µM in DMSO 0.5%, which also represented the control sample. All samples were applied daily in the same volume for 72 h. For each analyzed sample, 5 eggs were used, and all samples were applied in triplicate.

Macroscopic evaluation was daily performed in ovo by means of stereomicroscopy (ZEISS SteREO Discovery.V8, Göttingen, Germany) and all images were registered and processed by Axiocam 105 color, AxioVision SE64. Rel. 4.9.1 Software, (ZEISS Göttingen, Germany), ImageJ (ImageJ Version 1.50e, https://imagej.nih.gov/ij/index.html, accessed on 12 May 2021) and GIMP software (GIMP v 2.8, https://www.gimp.org/, accessed on 12 May 2021). 

#### HET-CAM Assay 

Compounds **6** and **7** were also evaluated in terms of biocompatibility on mucosal tissues by using the in vivo Hen’s Egg Chorioallantoic Membrane Test (HET-CAM assay). The assay assesses a potential irritant effect on the vascular plexus of the chorioallantoic membrane [74]. The HET-CAM method was carried out following ICCVAM recommendations published in November 2016 in Appendix G and adapted to our laboratory conditions [68,77,78]. Thus, 300 µL of control or test solution, respectively, was applied on CAM and the alterations induced at the CAM level were monitored by means of stereomicroscopy (Discovery 8 Stereomicroscope, Zeiss), registering significant images (Axio CAM 105 color, Zeiss), before the application and after 5 min contact with the samples. All images were processed using Zeiss ZEN software, Gimp 2.8 and ImageJ software. Negative control was represented by distilled water and solvent control DMSO 0.5%, while the positive control by the sodium lauryl sulphate (SLS) 0.5% in distillate water. The test substances were tested in concentrations of 1 µM. The observation time of the produced reactions was 5 min (300 s) and the time, at which the occurrence of a particular reaction took place was recorded in seconds. Finally, the following reactions were monitored: hemorrhage—H (blood vessel bleeding), vascular lysis —L (disintegration of blood vessels), and coagulation —C (intra or extra- vascular protein denaturizing). A variety of analysis methods may be used to assess irritancy potential of test substances. One analysis method that has been used extensively used is an irritation score (IS) calculated according to the following formula:

$$\mathrm{IS}=5\times \frac{301-\mathrm{SecH}}{300}+7\times \frac{300-\mathrm{SecL}}{300}+9\times \frac{301-\mathrm{SecC}}{300}$$

where H = hemorrhage; L = vessel lysis; C = coagulation; Hemorrhage time (Sec H) = onset of hemorrhage reactions on CAM (in sec); Lysis time (Sec L) = onset of vessel lysis on CAM (in sec); Coagulation time (Sec C) = onset of coagulation formation on CAM (in sec). The formula comprises a factor indicating the impact of the observed effect on vascular damage, e.g., coagulation has the highest impact expressed by the multiplication factor 9. The IS values range on a scale between 0 and 21.

### 3.7. Molecular Docking

The molecular docking protocol employed in this study was previously reported [79,80]. Briefly, protein targets structures were available from the RCSB Protein Data Bank [81] (Table 7). Protein structure optimization was achieved, using Autodock Tools v1.5.6 (The Scripps Research Institute, La Jolla, CA, USA). Water molecules, the co-crystallized ligand and unnecessary protein chains were removed from the protein structure file. Subsequently, Gesteiger charges were added to the protein. The target files were saved as the required pdbqt file format. The structures of compounds **6** and **7** were sketched using Biovia Draw (Dassault Systems Biovia, San Diego, CA, USA) and converted into 3D structure files (uff force field) using PyRx’s embedded Open Babel function. Molecular docking was performed with PyRx v0.8 (The Scripps Research Institute, La Jolla, CA, USA) using Autodock Vina’s embedded scoring function [82]. The docking method was validated by re-docking the co-crystallized ligands into their original binding sites. The calculated docking pose was compared with the experimental (co-crystallized) binding pose. Docking studies were performed for each instance, only if the root mean square deviation (RMSD) values between the co-crystallized ligand’s experimental and docked pose did not exceed a 2 Å threshold. The search space grid box was defined in terms of coordinates and size (Table 7) to best fit the active binding site of the native ligand. Docking scores were recorded as ΔG binding energy values (kcal/mol). Ligand–protein binding interactions were analyzed using Accelerys Discovery Studio 4.1 (Dassault Systems Biovia, San Diego, CA, USA).

## 4. Conclusions

The current study reported the synthesis and anticancer biological evaluation of a series methyl 3-oxo-platanoate and 3-oxo-21β-acetyl-20β,28-epoxy-18α,19βH-ursane triterpene derivatives. Compounds **6** and **7** emerged as the highest active tested agents with GI_50_ values ranging from 0.03 µM to 5.9 µM for compound **6** and 0.18–1.53 µM for compound 7 in the NCI-60 cancer cell line panel screening. These compounds were further evaluated in order to identify a possible antiproliferative mechanism of action. DAPI staining revealed that both compounds induced nuclei condensation and overall cell morphological changes consistent with apoptotic cell death. rtPCR analysis showed that both compounds induced upregulation of proapoptotic Bak and Bad genes while downregulating Bcl-XL and Bcl-2 antiapoptotic genes in the Sk-Mel-28 cell line. Molecular docking analysis revealed that both compounds exhibited high scores for Bcl-XL inhibition, while compound **7** showed higher in silico Bcl-XL inhibition potential as compared to the native inhibitor ATB-737. These results suggest that compounds may induce apoptotic cell death through targeted antiapoptotic protein inhibition, as well. Ex vivo assessment showed that while both compounds do not exhibit high irritation potential, they also do not induce angiogenesis impairment in the tested conditions. These results altogether reveal that such triterpenoid derivatives are potent antiproliferative agents and exert their activity by mainly regulating different aspects involved with apoptotic cancer cell death. Considering the obtained results, the compounds can be tested on enzymatic targets involved in apoptosis or in other signaling pathways overexpressed in cancer, after which in vivo studies can be performed on experimental animal models. The results to be obtained can gradually lead to the refinement of a triterpene derivative into a potential lead-like/drug-like candidate.

## Figures and Tables

**Table 1:** In vitro antitumor activity of compounds **1, 2, 4**–**8** against human cancer cells of 60 lines at a concentration of 10 μM.

Cell Line	Growth Percent						
	1	2	4	5	6	7	8
Leukemia							
CCRF-CEM	64.97	104.98	30.84	100.63	17.92	0.95	7.57
HL-60(TB)	39.32	133.26	47.50	-	14.49	−30.17	−2.67
K-562	39.40	103.93	17.96	-	6.51	−21.61	18.29
MOLT-4	42.52	100.88	25.21	-	16.72	−31.34	5.47
RPMI-8226	47.53	102.14	9.55	105.93	5.06	−28.73	2.88
SR	62.46	92.32	-	-	-	−14.07	12.35
Non-Small Cell Lung Cancer							
A549/ATCC	88.63	93.03	57.76	103.89	−24.64	−9.34	51.40
EKVX	91.35	71.73	101.00	104.96	−1.58	−76.32	85.77
HOP-62	100.79	80.72	81.73	104.25	−13.55	−85.57	102.96
HOP-92	70.21	95.88	79.10	65.36	−19.97	−79.86	66.73
NCI-H226	91.65	95.16	76.56	99.66	−13.51	−44.44	48.05
NCI-H23	84.77	90.60	59.51	98.55	−48.71	−48.31	53.20
NCI-H322M	95.70	106.71	88.41	88.29	46.39	−80.71	93.90
NCI-H460	84.90	94.87	40.71	109.09	−45.33	−78.43	70.90
NCI-H522	75.59	-	37.96	87.83	−34.35	−59.72	47.50
Colon Cancer							
COLO 205	71.47	111.81	56.27	−2.30	−56.84	−45.07	82.53
HCC-2998	92.22	109.84	69.92	104.00	−67.18	−55.45	75.76
HCT-116	41.92	100.51	14.40	100.13	−45.63	−87.45	3.92
HCT-15	38.30	111.38	38.53	105.61	−15.30	−46.22	23.22
HT29	80.13	108.96	99.49	98.06	26.36	−45.47	16.53
KM-12	78.19	102.54	54.39	99.96	−55.61	−74.98	60.52
SW-620	68.02	95.81	32.25	97.06	−26.39	−67.32	25.33
CNS Cancer							
SF-268	97.23	90.95	81.93	96.31	7.10	−80.80	68.63
SF-295	96.73	99.35	79.88	109.70	6.07	−98.52	75.30
SF-539	82.82	95.15	77.76	99.66	−32.36	−96.48	81.03
SNB-19	88.73	98.03	71.61	75.34	2.48	−87.62	67.72
SNB-75	106.15	72.80	72.76	78.39	32.69	−98.68	110.35
U251	77.27	107.36	58.63	69.38	−68.02	−87.90	56.61
Melanoma							
LOX IMVI	58.17	94.48	51.47	89.02	−85.19	-	-
MALME-3M	81.27	109.60	54.41	−11.31	−33.54	−95.19	29.95
M14	74.76	107.08	60.79	34.35	−20.11	−80.31	56.69
MDA-MB-435	83.22	100.67	34.50	17.83	−7.30	−69.71	72.63
SK-MEL-2	92.21	105.22	87.32	9.51	−25.53	-61.15	68.06
SK-MEL-28	80.73	109.33	66.23	36.66	−17.77	−96.28	31.54
SK-MEL-5	47.27	100.12	37.02	8.55	−82.25	−98.85	39.76
UACC-257	84.73	103.91	49.29	−8.91	13.16	−58.90	58.14
UACC-62	49.11	85.81	41.49	7.16	−25.57	−67.85	34.54
Ovarian Cancer							
IGROV1	51.77	88.93	80.59	107.89	1.25	−87.88	85.80
OVCAR-3	84.02	119.38	46.73	102.55	−43.66	−93.24	49.04
OVCAR-4	75.77	85.13	49.03	111.19	18.62	−39.20	26.68
OVCAR-5	97.46	96.55	120.62	102.21	−4.00	−62.74	114.28
OVCAR-8	92.52	104.68	35.05	99.33	−13.55	−34.25	38.71
NCI/ADR-RES	96.25	102.89	78.87	95.25	2.97	−39.38	52.54
SK-OV-3	98.97	118.18	77.76	102.58	58.93	−23.10	93.73
Renal Cancer							
786-0	97.17	89.64	63.59	103.94	−38.97	−92.26	84.87
A498	-	67.96	72.89	96.19	−59.07	−91.17	80.56
ACHN	86.11	99.80	41.58	44.33	−22.16	−100.00	32.49
CAKI-1	74.02	66.38	55.59	55.81	9.32	−78.75	51.03
RXF 393	89.49	76.40	75.20	109.55	−91.75	−93.62	30.89
SN12C	88.79	87.69	62.68	100.53	−17.67	−77.80	57.75
TK-10	109.75	76.40	31.89	97.40	−14.49	−60.49	132.71
UO-31	95.19	66.99	58.69	77.02	−97.34	−100.00	56.64
Prostate Cancer							
PC-3	48.79	87.23	21.26	92.69	−3.56	−14.12	24.42
DU-145	99.41	98.96	73.94	102.22	−28.80	−67.84	68.80
Breast Cancer							
MCF7	56.74	90.80	23.65	101.23	9.88	−54.19	19.18
MDA-MB-231/ATCC	88.35	85.22	82.10	82.90	−30.71	−81.32	76.56
HS 578T	98.58	103.91	81.98	95.83	11.24	−49.05	74.77
BT-549	96.17	-	57.24	104.50	−20.09	−92.12	46.03
T-47D	74.66	106.29	30.20	84.80	24.61	−43.57	24.42
MDA-MB-468	57.26	108.53	20.53	19.35	−5.83	−76.12	55.75

**Table 2:** In vitro anticancer activity of compounds **6** and **7** against 60 human cancer cell lines, in second stage five dose regimen assessment (0.01–100 μM) and 2 healthy cell lines.

Subpanel/Cell Lines (μM)	GI_50_ Compound 6	GI_50_ Compound 7	GI_50_ Doxorubicin	Subpanel/Cell Lines (μM)	GI_50_ Compound 6	GI_50_ Compound 7	GI_50_ Doxorubicin
Leukemia				Melanoma			
CCRF-CEM	0.17	0.31	0.08	LOX IMVI	0.89	0.21	0.07
HL-60(TB)	0.56	0.25	0.12	MALME-3M	0.80	0.21	0.12
K-562	0.03	0.33	0.19	M14	1.74	0.31	0.18
MOLT-4	0.17	0.30	0.03	MDA-B-435	1.09	0.20	0.25
RPMI-8226	0.16	0.24	0.08	SK-MEL-2	1.30	0.47	0.17
SR	0.16	0.27	0.03	SK-MEL-28	0.94	0.24	0.21
Non-Small Cell Lung Cancer				SK-MEL-5	0.82	0.46	0.08
A549/ATCC	1.27	0.39	0.06	UACC-257	1.67	0.27	0.14
EKVX	1.72	0.41	0.41	UACC-62	0.60	0.40	0.12
HOP-62	1.56	0.69	0.07	Ovarian Cancer			
HOP-92	0.82	0.53	0.10	IGROV1	0.96	0.27	0.17
NCI-H226	1.02	1.48	0.05	OVCAR-3	0.88	0.27	0.39
NCI-H23	1.04	0.35	0.15	OVCAR-4	1.49	0.34	0.37
NCI-H322M	1.82	1.48	-	OVCAR-5	1.46	0.41	0.41
NCI-H460	0.89	0.83	0.02	OVCAR-8	1.60	0.34	0.10
NCI-H522	1.36	0.66	0.03	NCI/ADR-RES	1.70	0.43	7.16
Colon cancer				SK-OV-3	5.90	1.43	0.22
COLO 205	1.43	0.36	0.18	Renal Cancer			
HCC-2998	0.89	0.67	0.26	786-0	0.92	0.31	0.13
HCT-116	0.23	0.20	0.08	A498	0.78	0.18	0.10
HCT-15	1.11	0.24	6.46	ACHN	1.41	0.19	0.08
HT29	1.50	0.32	0.12	CAKI-1	1.42	1.09	0.95
KM-12	0.91	1.08	0.27	RXF 393	0.83	0.27	0.10
SW-620	0.98	0.30	0.09	SN12C	0.94	0.39	0.07
CNS Cancer				TK-10	1.83	0.65	-
SF-268	1.31	1.01	0.10	UO-31	0.64	0.17	0.49
SF-295	1.69	0.44	0.10	Breast Cancer			
SF-539	1.02	0.24	0.12	MCF7	0.91	0.38	0.03
SNB-19	0.85	0.69	0.04	MDA-MB-231/ATCC	1.03	0.47	0.51
SNB-75	1.02	1.35	0.07	HS 578T	1.44	0.46	0.33
U251	1.04	0.36	0.04	BT-549	0.73	0.22	0.23
Prostate Cancer				T -47D	1.98	0.43	0.06
PC-3	0.26	0.42	0.32	MDA-MB-468	0.86	0.27	0.05
DU-145	0.93	1.53	0.11				
Healthy cells							
HaCaT	18.56	15.42	-	1BR3	23.05	20.87	-

**Table 3:** Selectivity of compounds **6** and **7**.

Panel	Leukemia	Non-Small Cell Lung Cancer	Colon Cancer	CNS CANCER	Melanoma	Ovarian Cancer	Renal Cancer	Prostate Cancer	Breast Cancer
**SI for 6**	5.30	0.87	1.10	0.96	1.01	0.57	1.01	1.86	0.96
**SI for 7**	1.73	0.65	1.07	0.72	1.59	0.98	1.23	0.50	1.34

**Table 4:** Expression of the BAK, Bad, BCL-2, and BCL-XL genes for different treatments. Marked boxes (*) indicate significant differences as compared to the reference group (Test *t* test, ***- *p* < 0.001, **- *p* < 0.01, *- *p* < 0.05).

Sample	Bcl-XL		Bcl-2		Bak		Bad	
	Mean	Std. Dev	Mean	Std. Dev	Mean	Std. Dev	Mean	Std. Dev
**A375**								
DMSO 1	1.733	(1.030)	0.843	(0.185)	1.980	(0.030)	4.840	(2.456)
compound **7**	28.893 **	(6.866)	81.120 ***	(15.501)	17.743 ***	(0.979)	155.433 *	(67.665)
DMSO 1	4.550	(2.694)	2.216	(0.485)	5.200	(0.085)	4.840	(2.456)
compound **6**	0.286 *	(0.136)	2.570	(2.670)	0.546 ***	(0.107)	0.150 *	(0.036)
**RPMI**								
DMSO 1	5.853	(2.274)	1.383	(0.828)	3.533	(0.604)		
compound **7**	1.360 *	(1.232)	0.313	(0.172)	0.990 **	(0.476)		
DMSO 1	4.836	(2.533)	1.600	(0.374)	1.766	(0.302)		
compound **6**	0.100 **	(0.117)	0.246 **	(0.040)	0.066 ***	(0.023)		
**Sk-Mel-28**								
DMSO 1	2.423	(1.890)	2.573	(1.015)	1.383	(0.249)	3.460	(1.935)
compound **7**	3.826	(2.557)	0.210 *	(0.100)	59.543 **	(16.781)	65.946 ***	(10.324)
DMSO 1	1.786	(1.058)	2.573	(1.015)	1.383	(0.249)	3.460	(1.935)
compound **6**	1.606	(1.430)	0.200 *	(0.135)	22.826 ***	(3.009)	15.716 **	(2.176)

**Table 5:** Recorded docking scores for compounds **6** and **7** (binding energy, ∆G kcal/mol).

Target PDB ID	Binding Free Energy ∆G (kcal/mol)		
	Co-Crystallized Ligand	Compound 6	Compound 7
2YXJ	−10.7	−10.4	−11.1
3EQG	−8.8	2.2	−7.9
2W3L	−10.3	−5.4	−7.3
4OQ5	−12.2	6.5	−8.3
4JT5	−8.5	4.6	−5.1
4FA6	−9.3	60.4	21.1

**Table 6:** The irritant potential of compounds.

Test Compound and Controls	Irritation Score	Type of Effect
Distillate water	0	Non irritant
SLS 0.5%	17.03	Strong irritant
DMSO 0.5%	0.68	Non irritant
C7	0	Non irritant
C6	0	Non irritant

**Table 7:** Molecular docking parameters and protein targets.

Protein	PDB ID	Grid Box Center Coordinates	Grid Box Size	Conformers Generated Per Ligand
Apoptosis regulator Bcl-X (Bcl-XL)	2YXJ	center_x = −10.2573467499 center_y = −18.1808412775 center_z = 9.46376920765	size_x = 18.8213065002 size_y = 30.6345174449 size_z = 10.2997599705	8
Dual specificity mitogen-activated protein kinase kinase 1 (MEK1)	3EQG	center_x = −4.16588471722 center_y = 59.3845284049 center_z = 34.6388482191	size_x = 14.6058170561 size_y = 16.2060321551 size_z = 8.70490192182	8
Apoptosis regulator Bcl-2 (Bcl-2)	2W3L	center_x = 37.1927426166 center_y = 26.8056607992 center_z = −12.8141402336	size_x = 15.4649240353 size_y = 13.2408048336 size_z = 13.3102000528	8
Induced myeloid leukemia cell differentiation protein (Mcl-1)	4OQ5	center_x = 11.6262580805 center_y = 3.65908070556 center_z = 7.90001930158	size_x = 15.5072934727 size_y = 13.9772309154 size_z = 10.4111215841	8
Mamalyan terget of rapamycin- target of rapamycin complex subunit LST8 (mTOR-LST8)	4JT5	center_x = 51.5677297505 center_y = −1.9363339261 center_z = −48.4627944976	size_x = 16.0737519631 size_y = 11.2048088799 size_z = 10.5072207821	8
Phosphatidylinositol 4,5-bisphosphate 3-kinase catalytic subunit gamma isoform (PI3Kγ)	4FA6	center_x = 44.8020362414 center_y = 13.746321532 center_z = 30.3837942752	size_x = 14.3022779071 size_y = 10.3234871012 size_z = 8.72203337452	8

## Data Availability

The data presented in this study are available on request from the corresponding authors.

## Supplementary Materials

The following are available online at https://www.mdpi.com/article/10.3390/ijms221910695/s1.
