# Supplementary material for: 3-Pyridinylidene Derivatives of Chemically Modified Lupane and Ursane Triterpenes as Promising Anticancer Agents by Targeting Apoptosis
Source: Int J Mol Sci. 2021 Oct 2;22(19):10695. doi: 10.3390/ijms221910695 (PMC8509773; doi:10.3390/ijms221910695)
Supplement: Supplementary file 1 [file ijms-22-10695-s001.zip › ijms-1352935-supplementary.pdf]

3-Pyridinylidene derivatives of chemically modified lupane and ursane triterpenes as promising anticancer agents by targeting apoptosis

Oxana Kazakova[1#*], Codruța Șoica[2,3#], Marat Babaev[1], Anastasiya Petrova[1], El'mira Khusnutdinova[1], Alexander Poptsov[1], Ioana Macașoi[2,3], George Drăghici[2,3], Ştefana Avram[2,3], Lavinia Vlaia[2,4], Alexandra Mioc[2,3], Marius Mioc[2,3*], Cristina Dehelean[2,3], Adrian Voicu[5]

[1]Ufa Institute of Chemistry UFRC RAS, pr. Oktyabrya 71, 450054 Ufa, Russian Federation

[2]Faculty of Pharmacy, "Victor Babes" University of Medicine and Pharmacy, 2nd Eftimie Murgu Square, Timisoara, 300041, Romania

[3] Res. Ctr. Pharmacotoxicol Evaluat, Facculty of Pharmacy, "Victor Babes" University of Medicine and Pharmacy Timisoara, 2nd Eftimie Murgu Sq., Timisoara 300041, Romania;

[4]Res. Ctr. Drug Formulation and Technology, Facculty of Pharmacy, "Victor Babes" University of Medicine and Pharmacy Timisoara, 2nd Eftimie Murgu Sq., Timisoara 300041, Romania;

[5]Faculty of Medicine, "Victor Babes" University of Medicine and Pharmacy, 2nd Eftimie Murgu Sq., Timisoara 300041, Romania

[#] Authors with equal contribution jointly sharing the position of first author

[*] Corresponding authors: obf@anrb.ru, marius.mioc@umft.ro

Abstract

Cancer persists as a global challenge due to the extent to which conventional anticancer therapies pose high risks counterbalanced with their therapeutic benefit. Natural occurring substances stand as an important safer alternative source for anticancer drug development. In the current study a series of modified lupane and ursane derivatives was subjected to *in vitro* screening on the NCI-60 cancer cell line panel. Compounds **6** and **7** emerged as highly active with GI_{50} values ranging from 0.03 μM to 5.9 μM for compound **6**, while compound **7** recorded GI_{50} values between 0.18 – 1.53 μM. Thus, these two compounds were further assessed in detail in order to identify a possible antiproliferative mechanism of action. DAPI staining revealed that both compounds induced nuclei condensation and overall cell morphological changes consistent with apoptotic cell death. rtPCR analysis showed that both compounds induced up-regulation of pro-apoptotic Bak and Bad genes while down-regulating Bcl-XL and Bcl-2 anti-apoptotic genes. Molecular docking analysis revealed that both compounds exhibited high scores for Bcl-XL inhibition, while compound **7** showed higher *in silico* Bcl-XL inhibition potential as compare to the native inhibitor ATB-737; suggesting that compounds may induce apoptotic cell death through targeted anti-apoptotic protein inhibition, as well.

Keywords: pentacyclic triterpenes, platanoate, ursane, Claisen–Schmidt reaction, NCI-60, anticancer activity, apoptosis, antiangiogenic, molecular docking

Table of content

**Figure S1.** NMR spectra of compound **4**	3
**Figure S2.** NMR spectra of compound **5**	6
**Figure S3.** NMR spectra of compound **6**	7
**Figure S4.** NMR spectra of compound **7**	10
**Figure S5.** NMR spectra of compound **8**	13
**Figure S6.** *In vitro* antitumor activity of compound **1** against human cancer cells of 60 lines at a concentration of 100 µM	14
**Figure S7.** *In vitro* antitumor activity of compound **2** against human cancer cells of 60 lines at a concentration of 100 µM	15
**Figure S8.** *In vitro* antitumor activity of compound **4** against human cancer cells of 60 lines at a concentration of 100 µM	16
**Figure S9.** *In vitro* antitumor activity of compound **5** against human cancer cells of 60 lines at a concentration of 100 µM	17
**Figure S10.** *In vitro* antitumor activity of compound **6** against human cancer cells of 60 lines at a concentration of 100 µM	18
**Figure S11.** *In vitro* antitumor activity of compound **7** against human cancer cells of 60 lines at a concentration of 100 µM	19
**Figure S12.** *In vitro* antitumor activity of compound **8** against human cancer cells of 60 lines at a concentration of 100 µM	20
**Figure S13.** *In vitro* anticancer activity of compound **6** against 60 human cancer cell lines in second stage in single concentration 0.01 – 100 µM	21
**Figure S14.** *In vitro* anticancer activity of compound **7** against 60 human cancer cell lines in second stage in single concentration 0.01 – 100 µM	24

Figure S1. NMR spectra of compound **4**

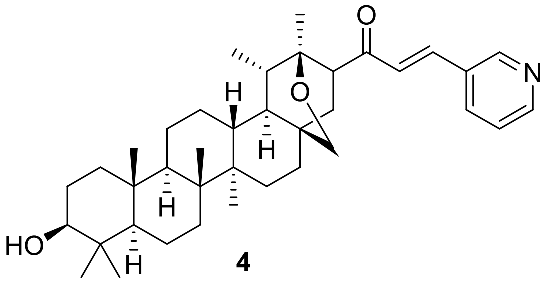

4

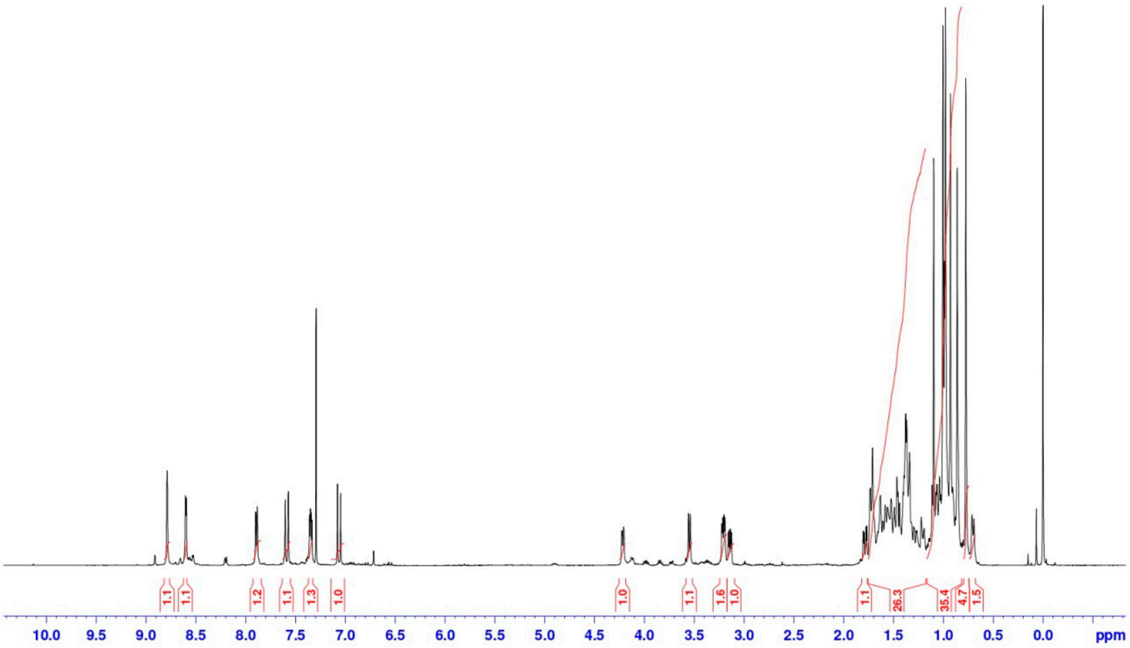

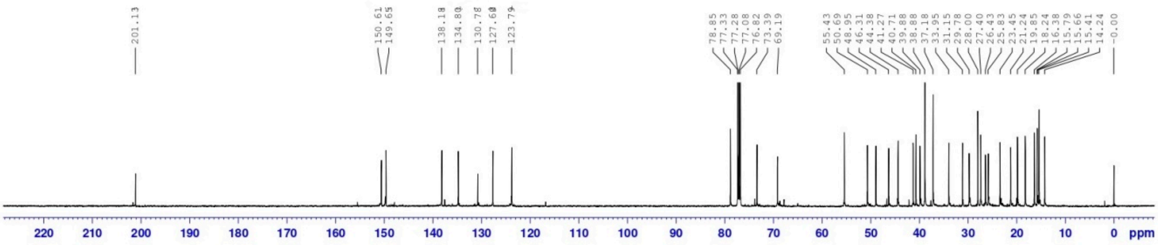

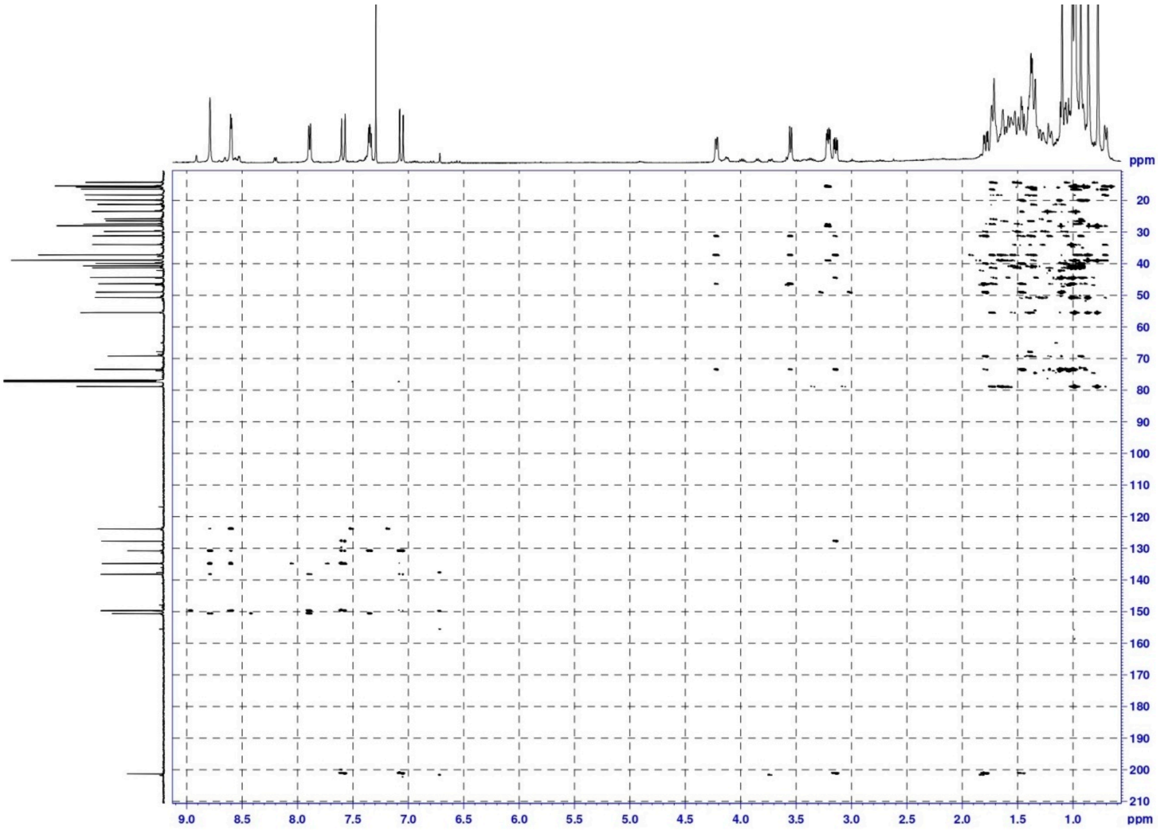

HMBC spectrum of compound **4**

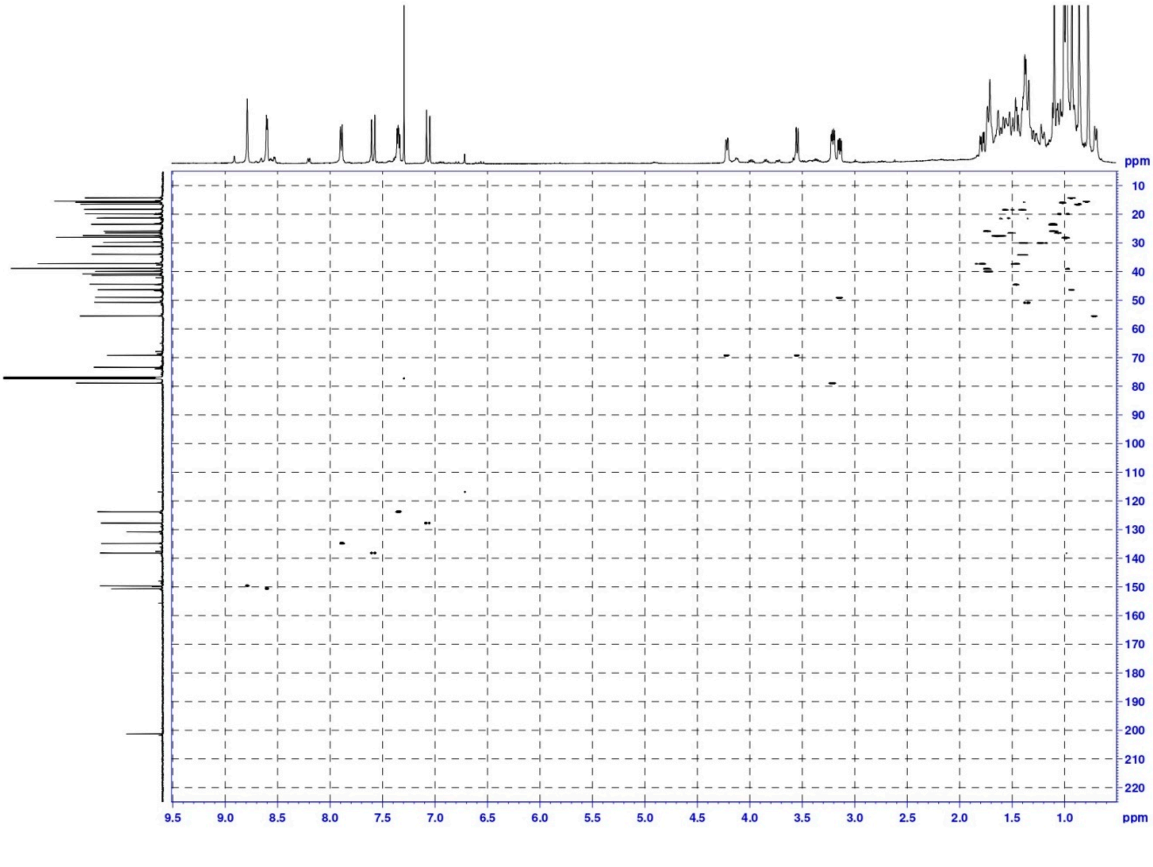

HSQC spectrum of compound **4**

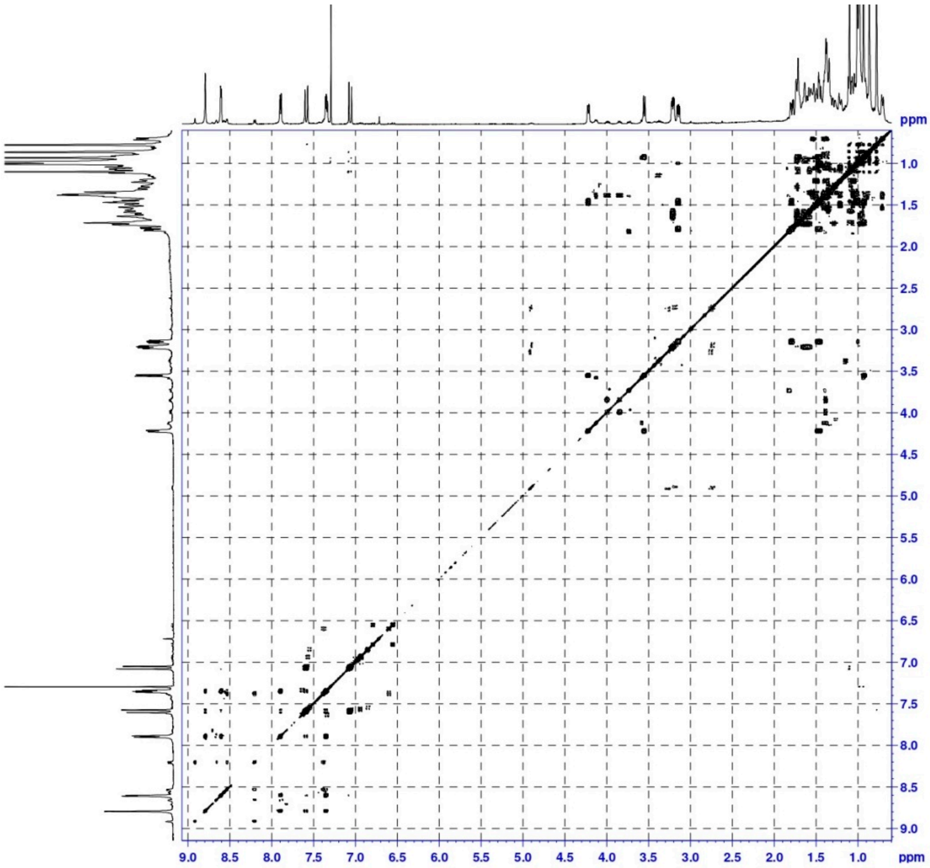

COSY spectrum of compound **4**

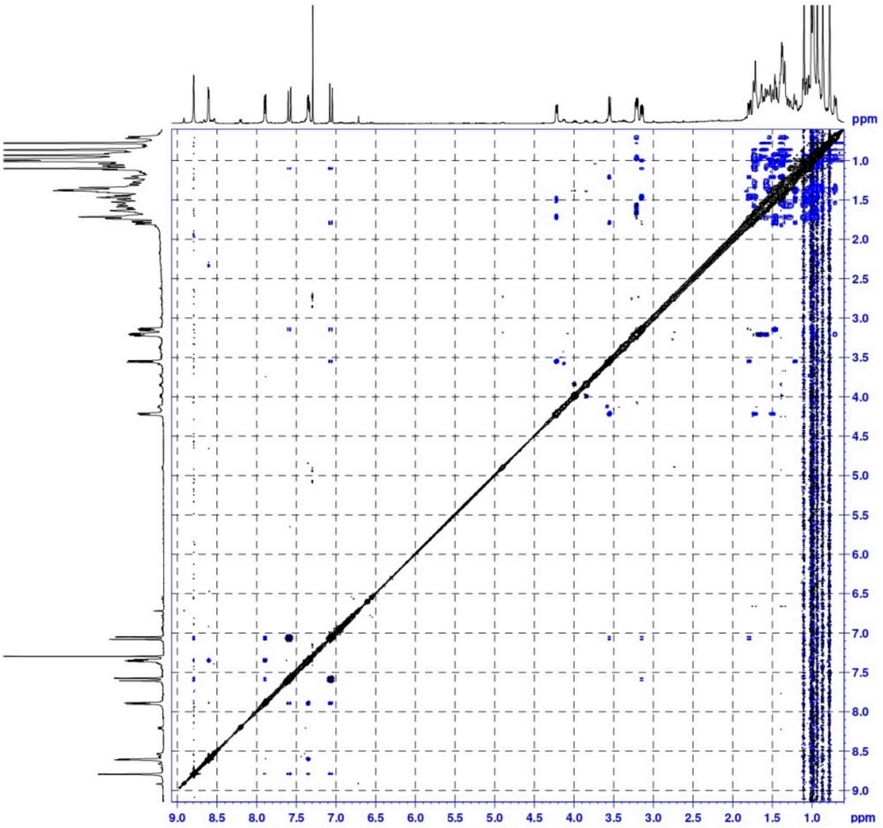

NOESY spectrum of compound **4**

Figure S2. NMR spectra of compound **5**

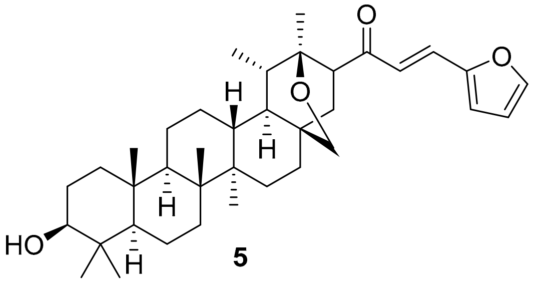

5

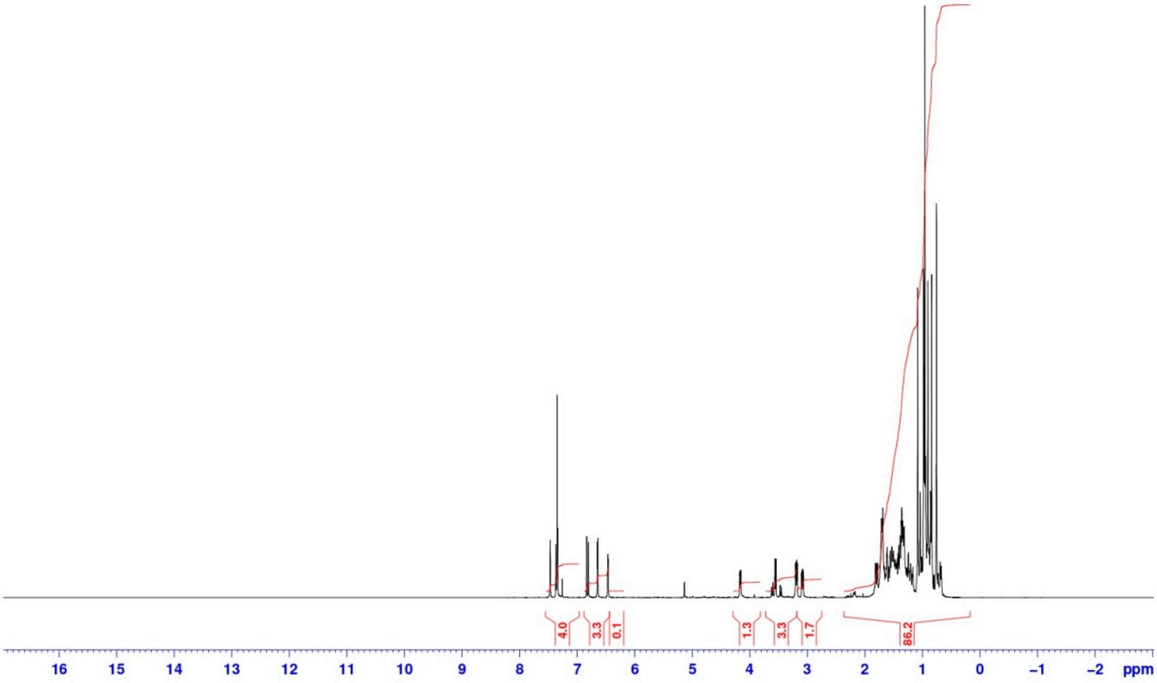

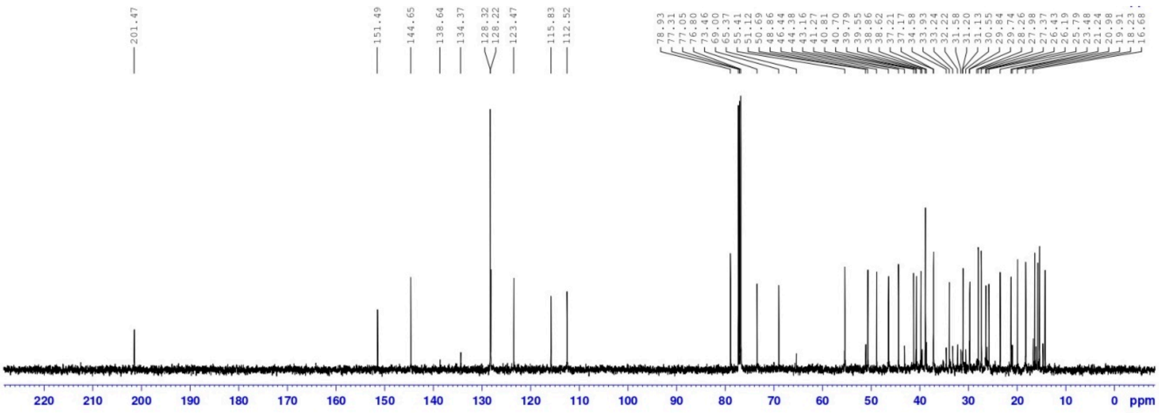

Figure S3. NMR spectra of compound **6**

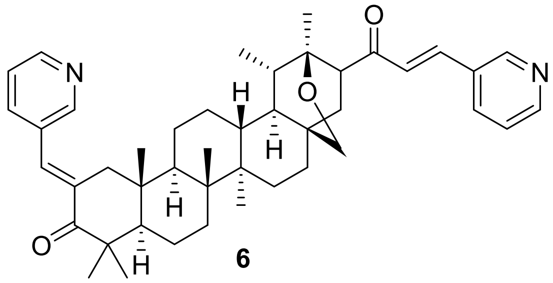

6

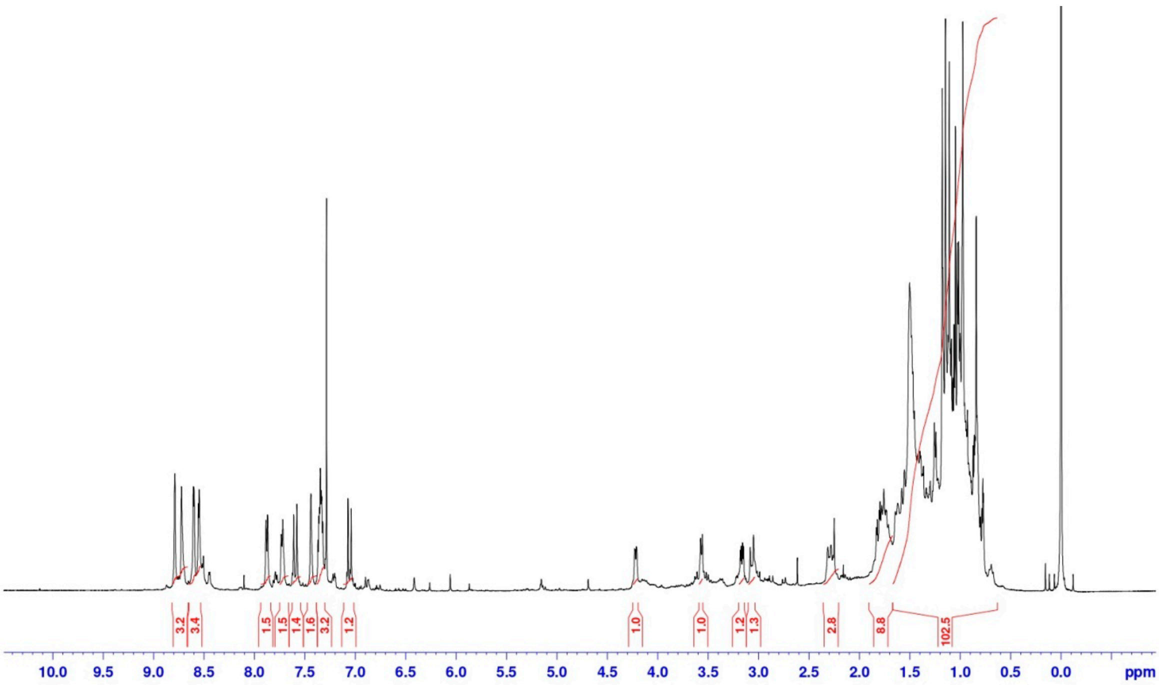

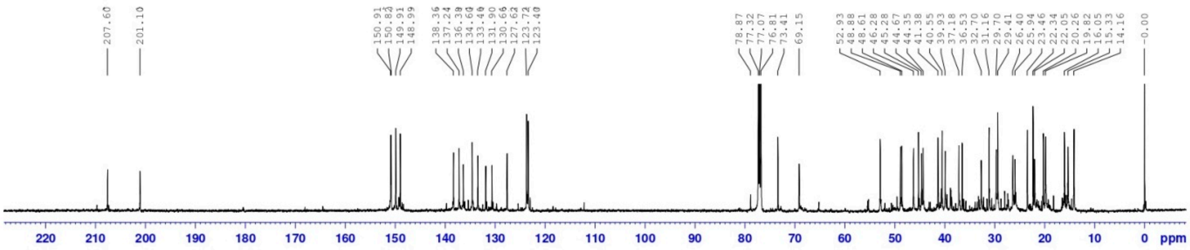

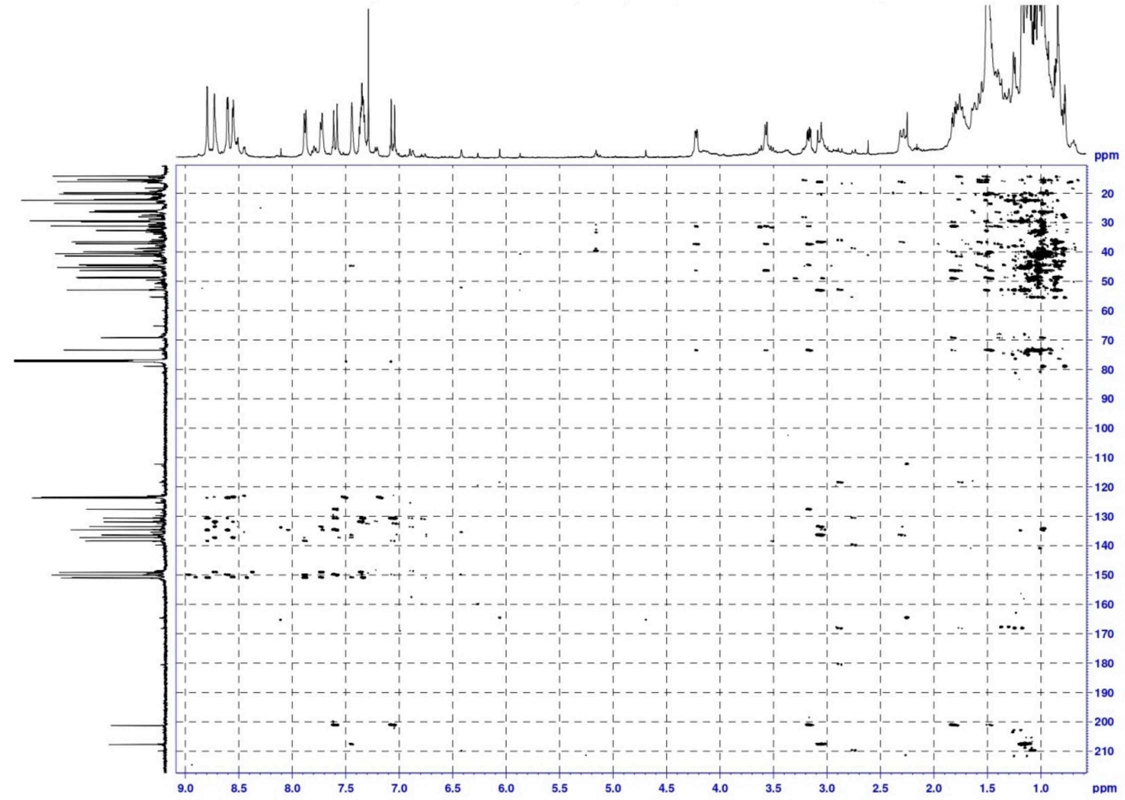

HMBC spectrum of compound **6**

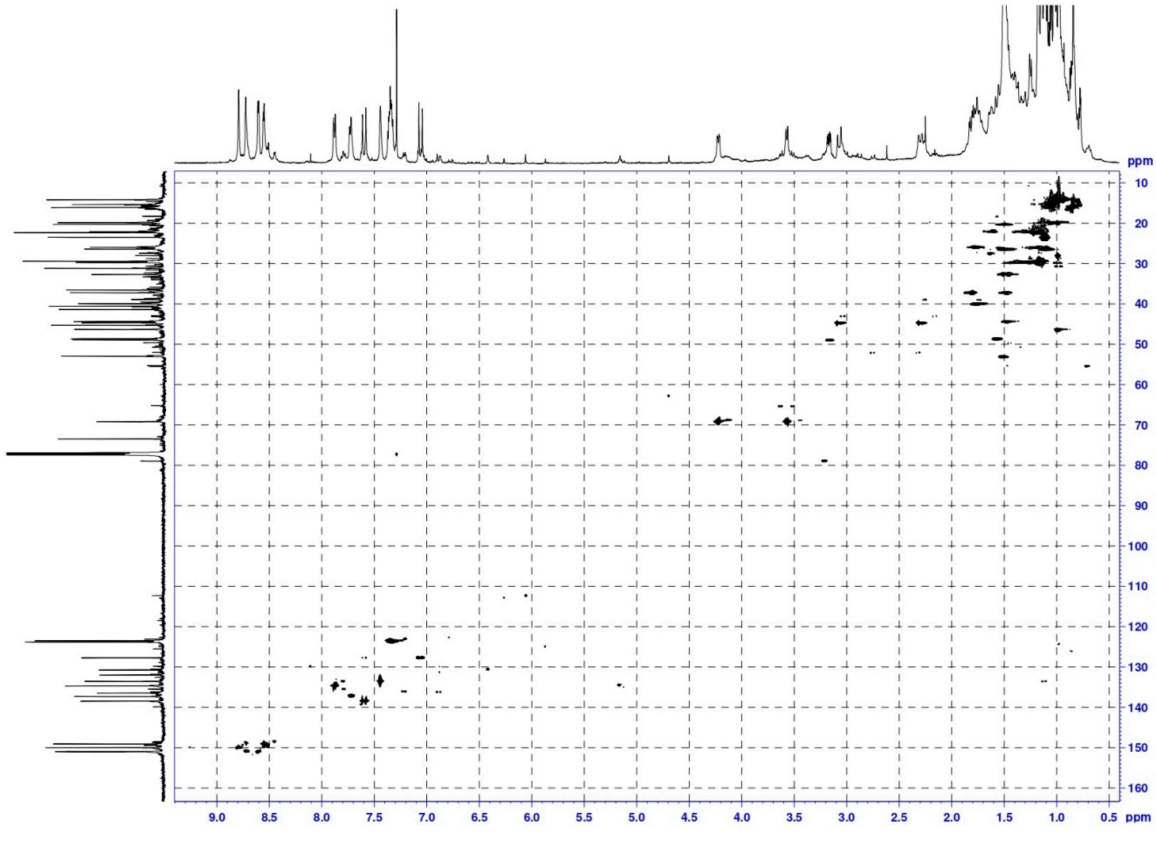

HSQC spectrum of compound **6**

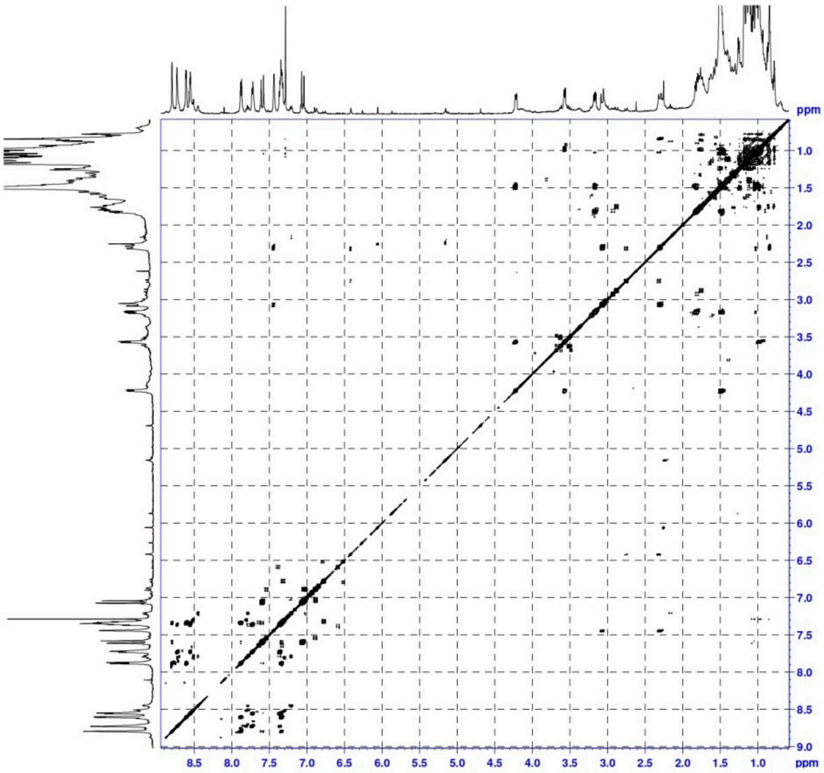

COSY spectrum of compound **6**

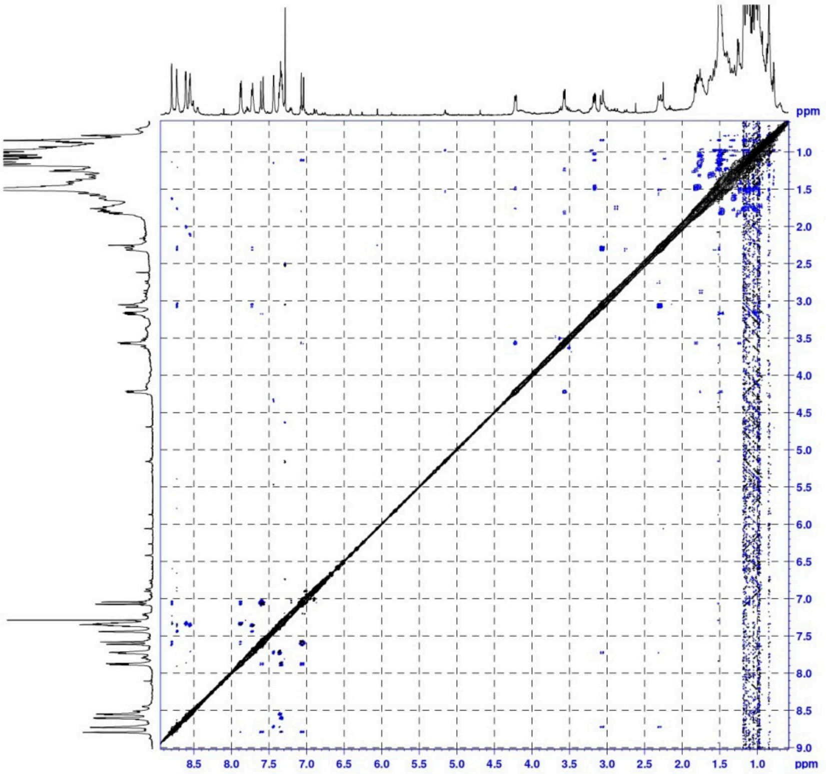

NOESY spectrum of compound **6**

Figure S4. NMR spectra of compound **7**

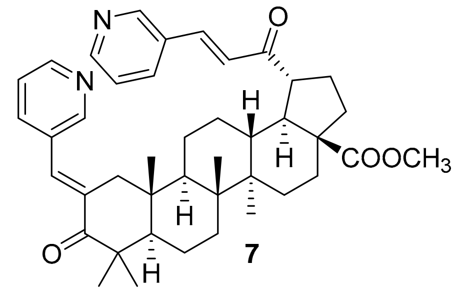

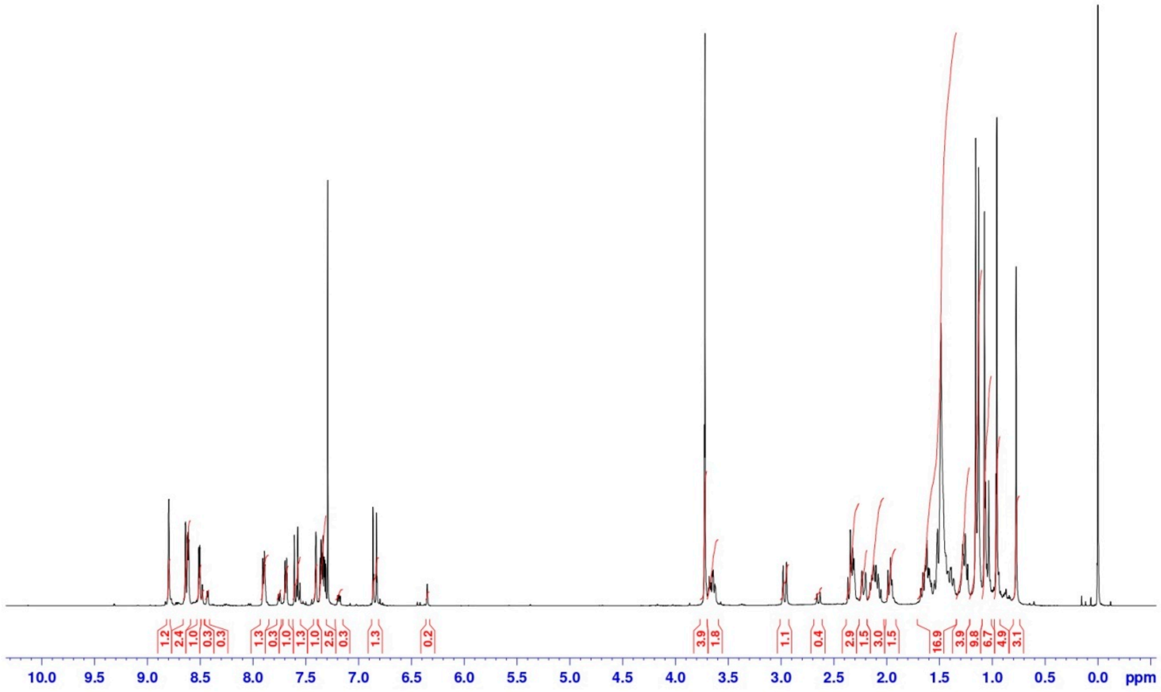

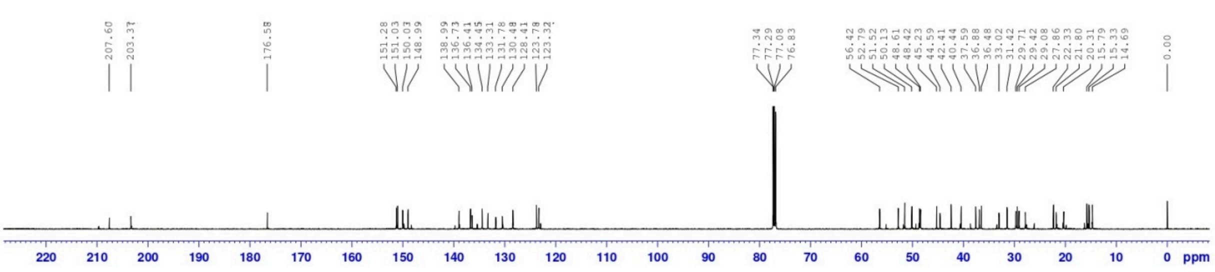

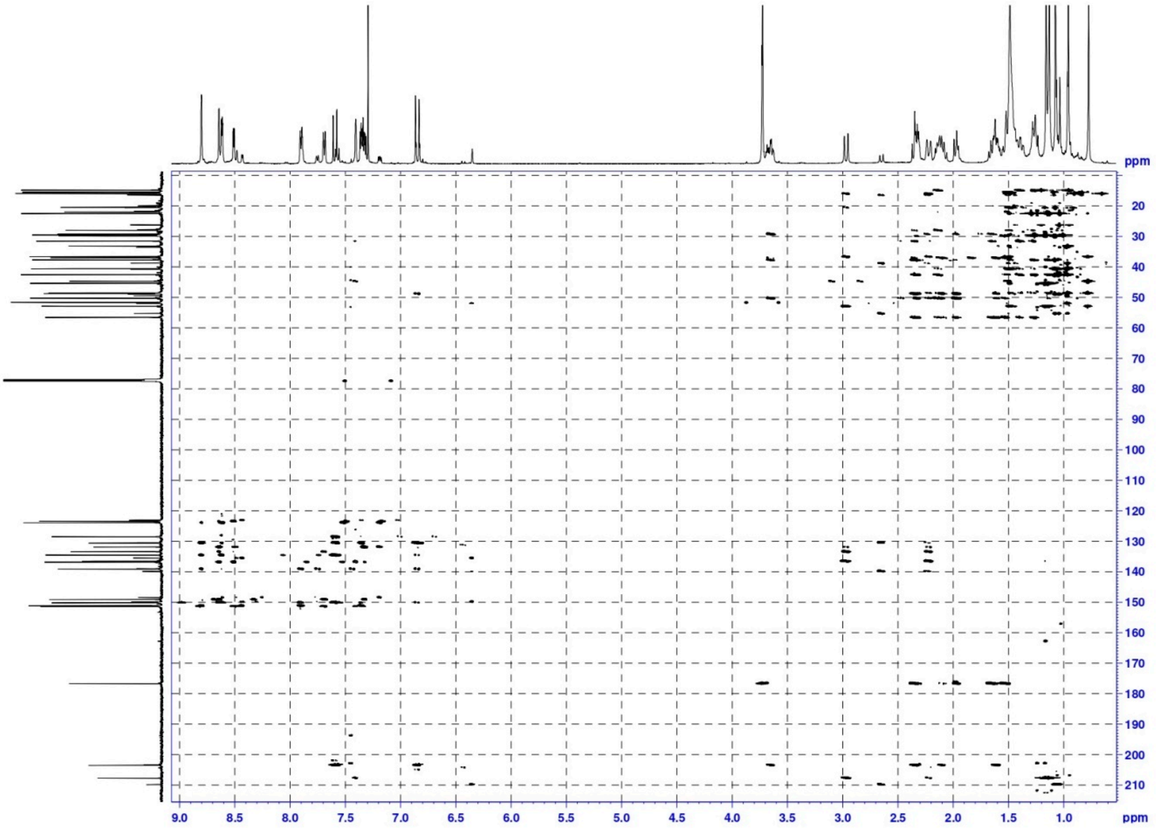

HMBC spectrum of compound **7**

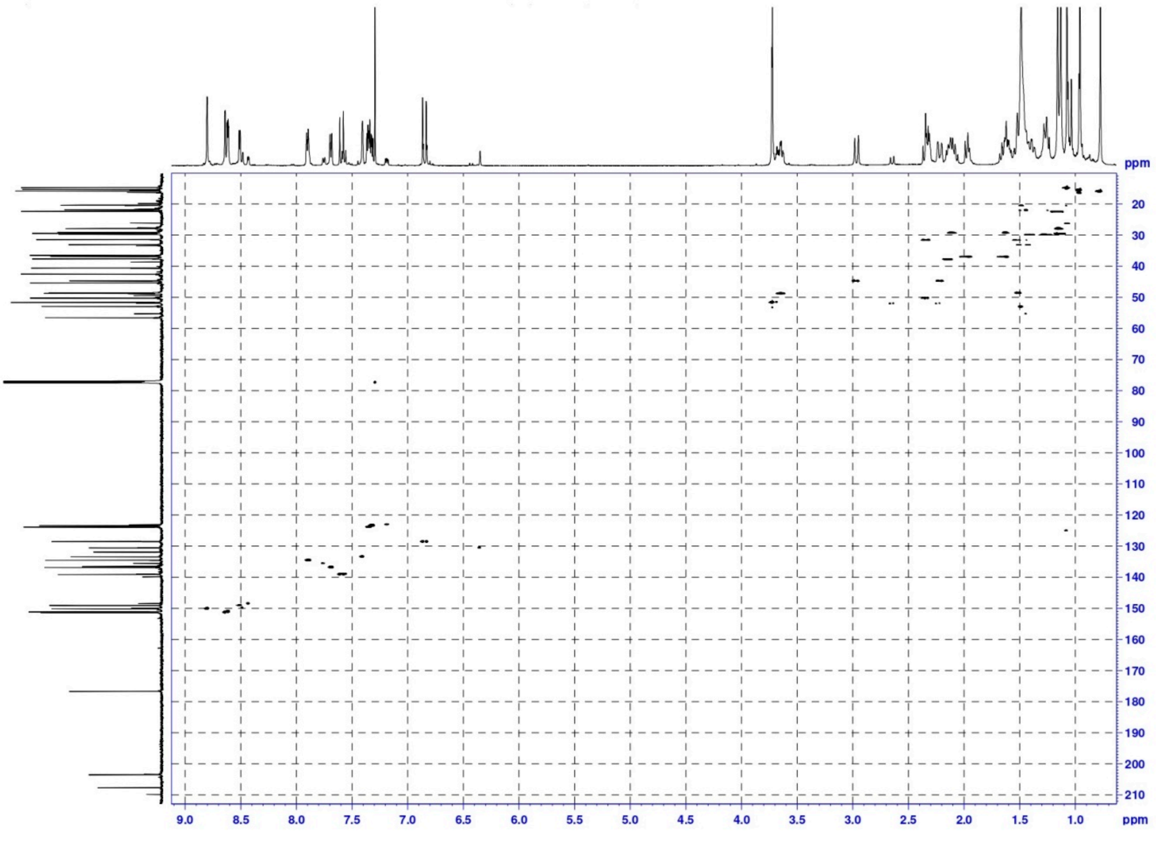

HSQC spectrum of compound **7**

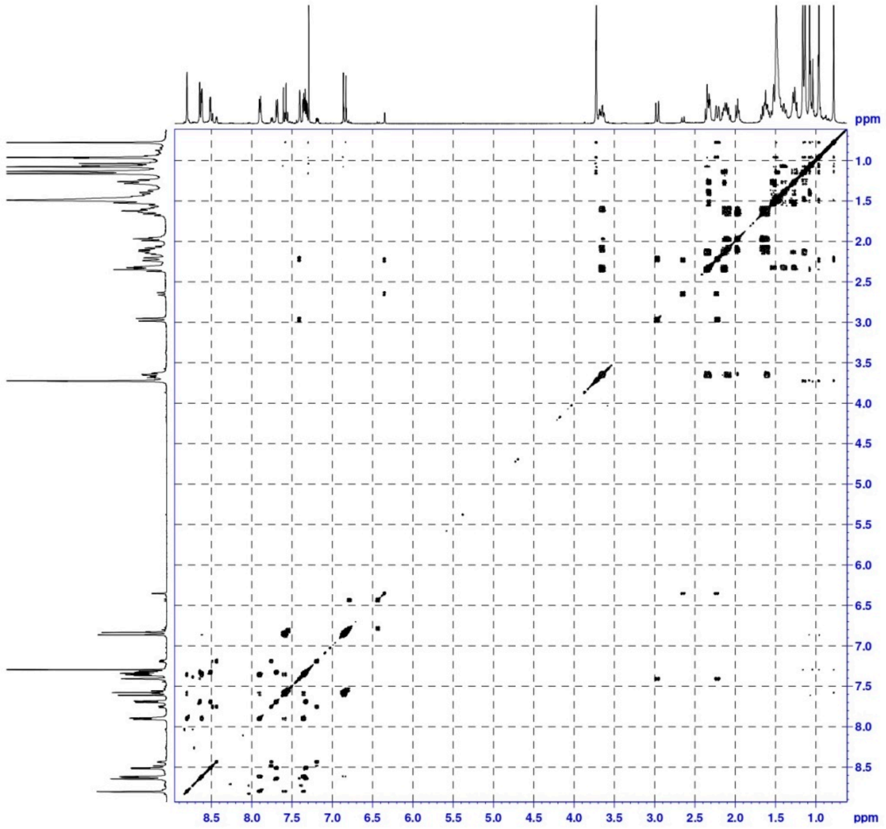

COSY spectrum of compound **7**

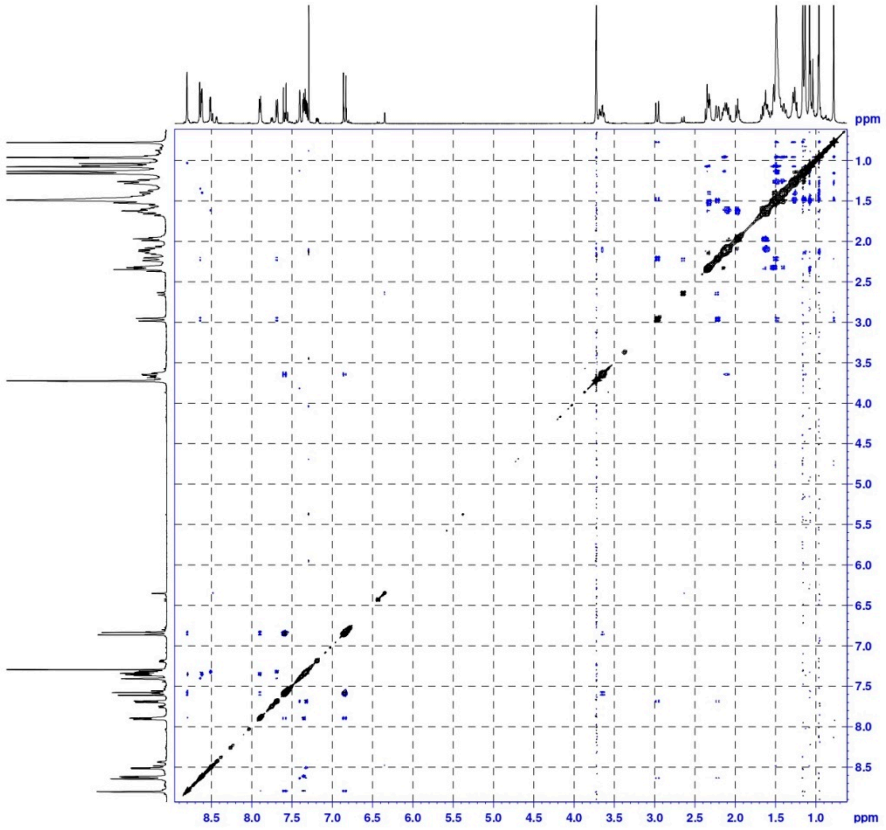

NOESY spectrum of compound **7**

Figure S5. NMR spectra of compound **8**

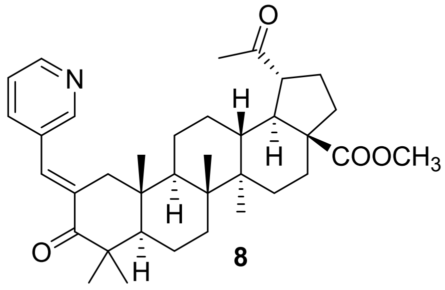

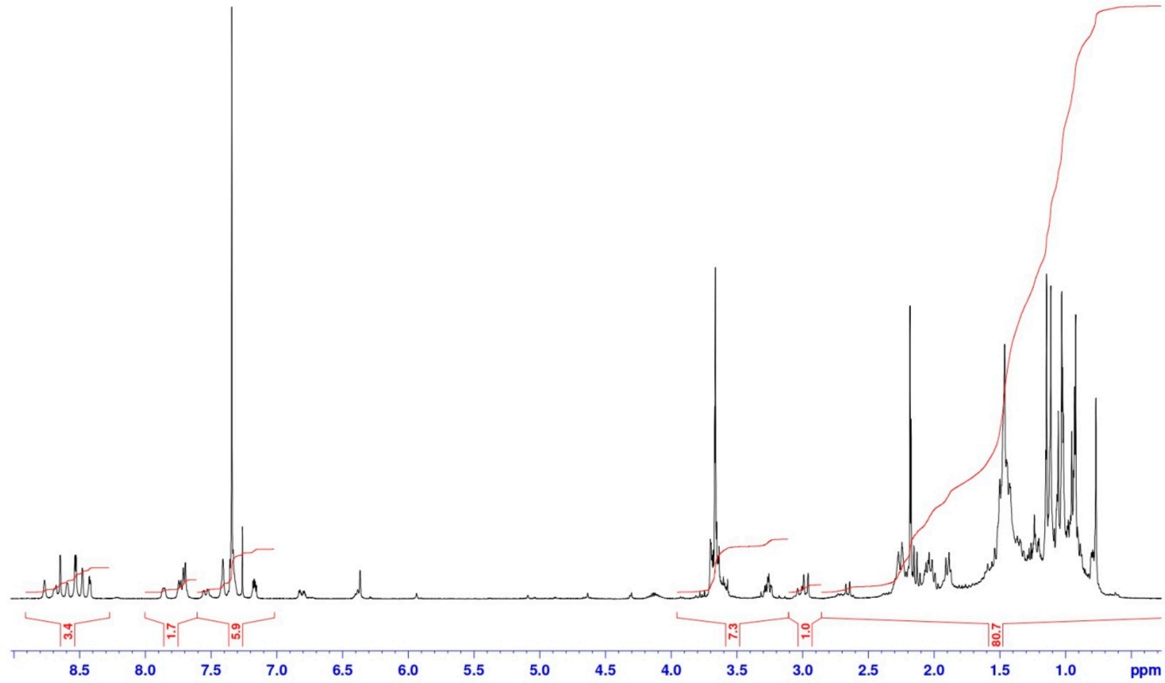

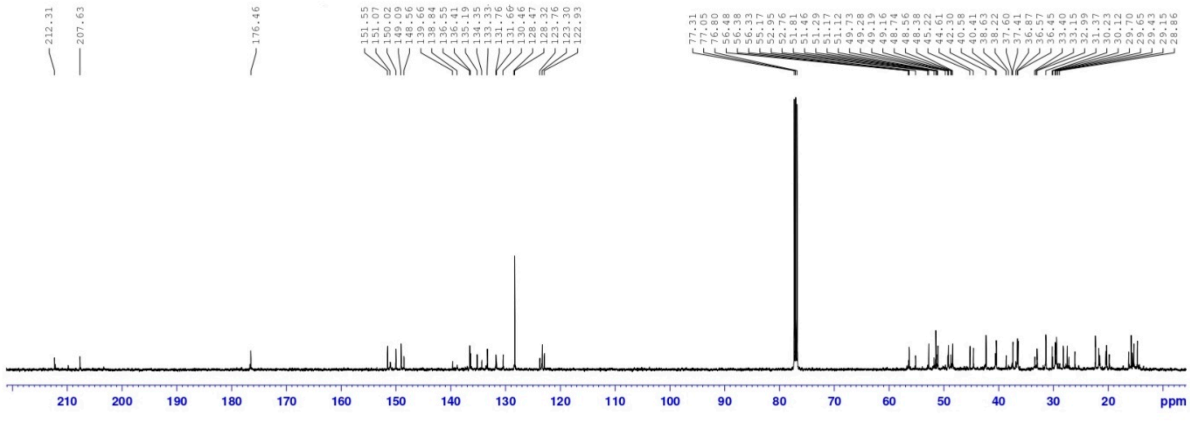

Figure S6. *In vitro* antitumor activity of compound **1** against human cancer cells of 60 lines at a concentration of 100 μM

Developmental Therapeutics Program	NSC: D-826775 / 1	Conc: 1.00E-5 Molar	Test Date: Nov 02, 2020
One Dose Mean Graph	Experiment ID: 2011OS61		Report Date: Nov 26, 2020

Panel/Cell Line	Growth Percent
**Leukemia**	
CCRF-CEM	64.97
HL-60(TB)	39.32
K-562	39.40
MOLT-4	42.52
RPMI-8226	47.53
SR	62.46
**Non-Small Cell Lung Cancer**	
A549/ATCC	88.63
EKVX	91.35
HOP-62	100.79
HOP-92	70.21
NCI-H226	91.65
NCI-H23	84.77
NCI-H322M	95.70
NCI-H460	84.90
NCI-H522	75.59
**Colon Cancer**	
COLO 205	71.47
HCC-2998	92.22
HCT-116	41.92
HCT-15	38.30
HT29	80.13
KM12	78.19
SW-620	68.02
**CNS Cancer**	
SF-268	97.23
SF-295	96.73
SF-539	82.82
SNB-19	88.73
SNB-75	106.15
U251	77.27
**Melanoma**	
LOX IMVI	58.17
MALME-3M	81.27
M14	74.76
MDA-MB-435	83.22
SK-MEL-2	92.21
SK-MEL-28	80.73
SK-MEL-5	47.27
UACC-257	84.73
UACC-62	49.11
**Ovarian Cancer**	
IGROV1	51.77
OVCAR-3	84.02
OVCAR-4	75.77
OVCAR-5	97.46
OVCAR-8	92.52
NCI/ADR-RES	96.25
SK-OV-3	98.97
**Renal Cancer**	
786-0	97.17
ACHN	86.11
CAKI-1	74.02
RXF 393	89.49
SN12C	88.79
TK-10	109.75
UO-31	95.19
**Prostate Cancer**	
PC-3	48.74
DU-145	99.41
**Breast Cancer**	
MCF7	56.74
MDA-MB-231/ATCC	88.35
HS 578T	98.58
BT-549	96.17
T-47D	74.66
MDA-MB-468	57.26
Mean	78.10
Delta	39.80
Range	71.45

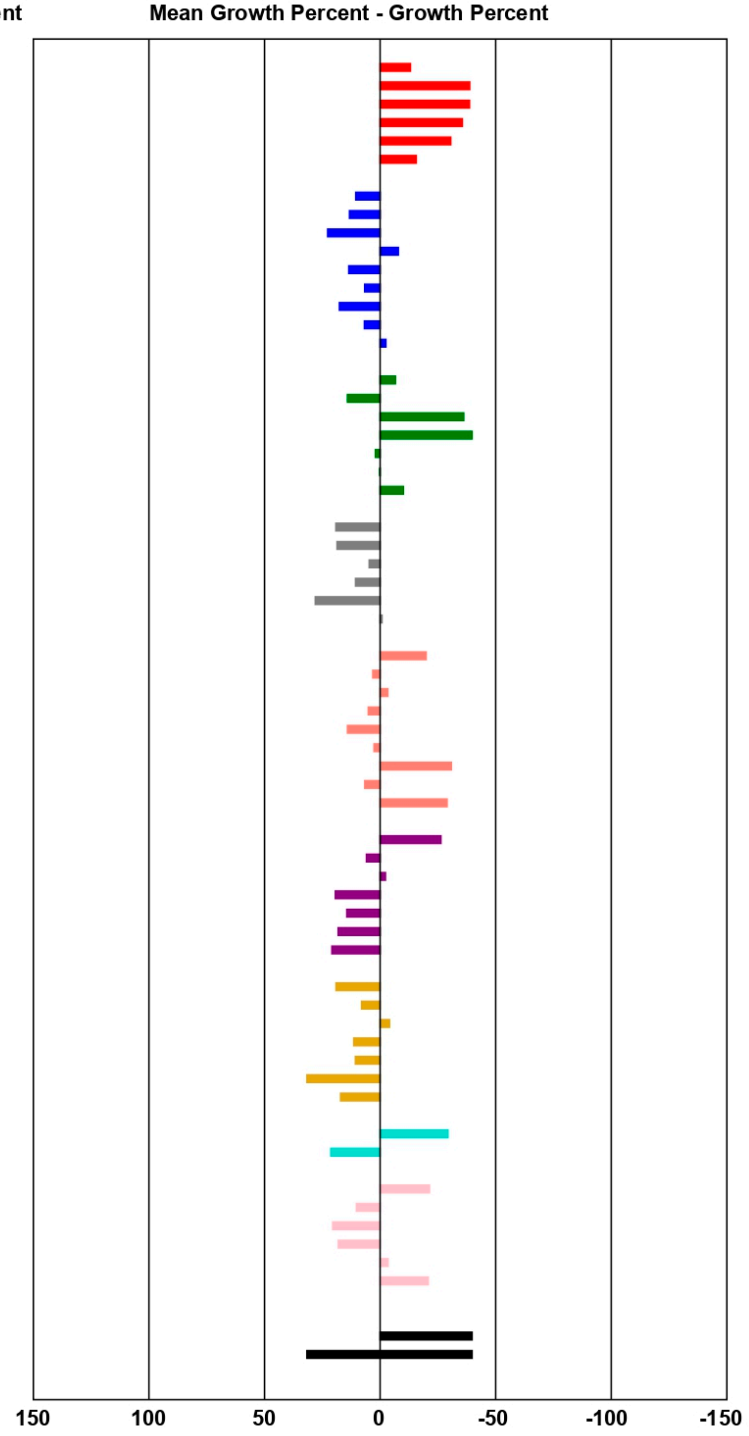

Figure S7. *In vitro* antitumor activity of compound **2** against human cancer cells of 60 lines at a concentration of 100 μM

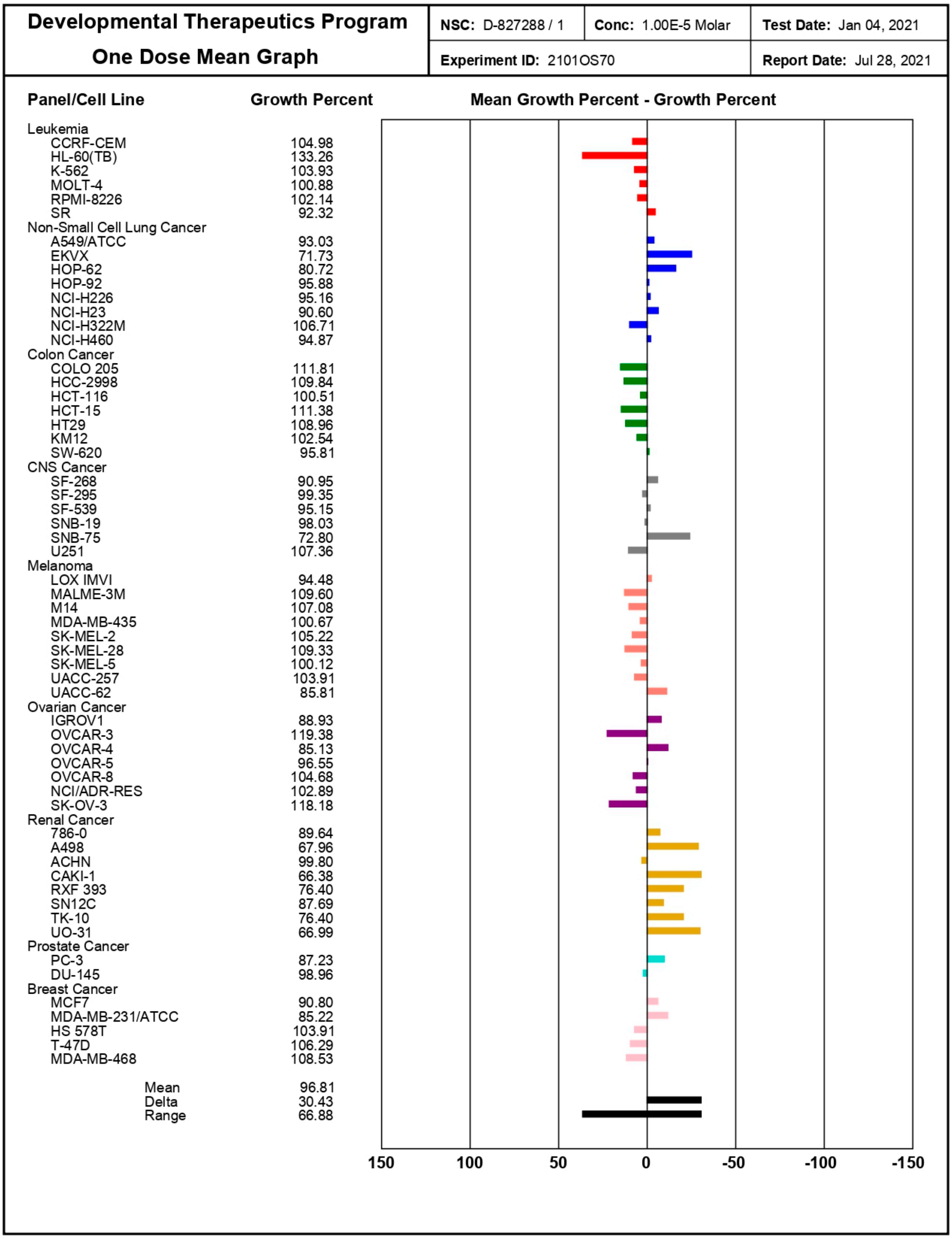

Figure S8. *In vitro* antitumor activity of compound **4** against human cancer cells of 60 lines at a concentration of 100 μM

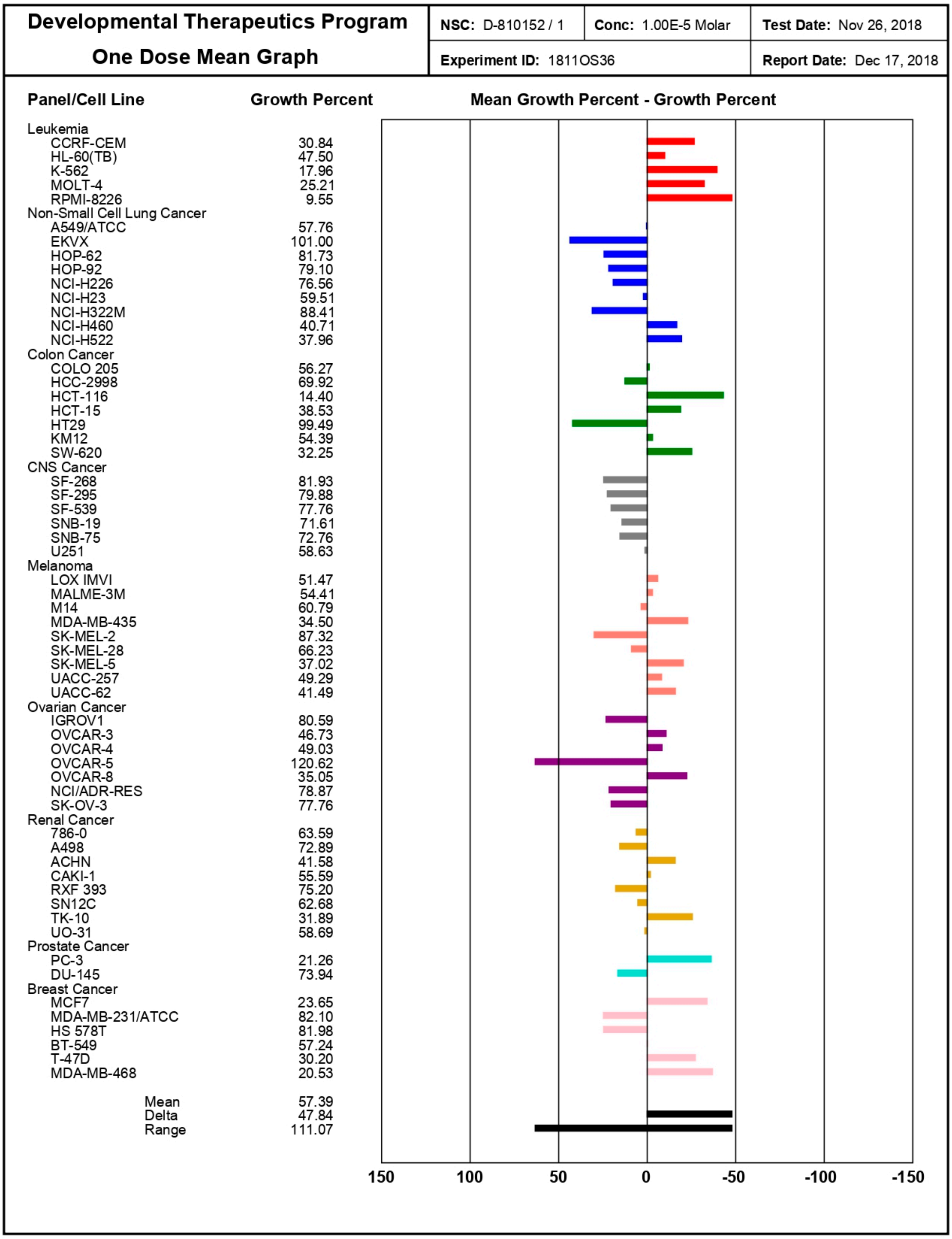

Figure S9. *In vitro* antitumor activity of compound **5** against human cancer cells of 60 lines at a concentration of 100 μM

Developmental Therapeutics Program	NSC: D-810151 / 1	Conc: 1.00E-5 Molar	Test Date: Nov 26, 2018
One Dose Mean Graph	Experiment ID: 1811OS36		Report Date: Dec 17, 2018

Panel/Cell Line	Growth Percent
Leukemia	
CCRF-CEM	100.63
RPMI-8226	105.93
Non-Small Cell Lung Cancer	
A549/ATCC	103.89
EKVX	104.96
HOP-62	104.25
HOP-92	65.36
NCI-H226	99.66
NCI-H23	98.55
NCI-H322M	88.29
NCI-H460	109.09
NCI-H522	87.83
Colon Cancer	
COLO 205	-2.30
HCC-2998	104.00
HCT-116	100.13
HCT-15	105.61
HT29	98.06
KM12	99.96
SW-620	97.06
CNS Cancer	
SF-268	96.31
SF-295	109.70
SF-539	99.66
SNB-19	75.34
SNB-75	78.39
U251	69.38
Melanoma	
LOX IMVI	89.02
MALME-3M	-11.31
M14	34.35
MDA-MB-435	17.83
SK-MEL-2	9.51
SK-MEL-28	36.66
SK-MEL-5	8.55
UACC-257	-8.91
UACC-62	7.16
Ovarian Cancer	
IGROV1	107.89
OVCAR-3	102.55
OVCAR-4	111.19
OVCAR-5	102.21
OVCAR-8	99.33
NCI/ADR-RES	95.25
SK-OV-3	102.58
Renal Cancer	
786-0	103.94
A498	96.19
ACHN	44.33
CAKI-1	55.81
RXF 393	109.55
SN12C	100.53
TK-10	97.40
UO-31	77.02
Prostate Cancer	
PC-3	92.69
DU-145	102.22
Breast Cancer	
MCF7	101.23
MDA-MB-231/ATCC	82.90
HS 578T	95.83
BT-549	104.50
T-47D	84.80
MDA-MB-468	19.35
Mean	79.86
Delta	91.17
Range	122.50

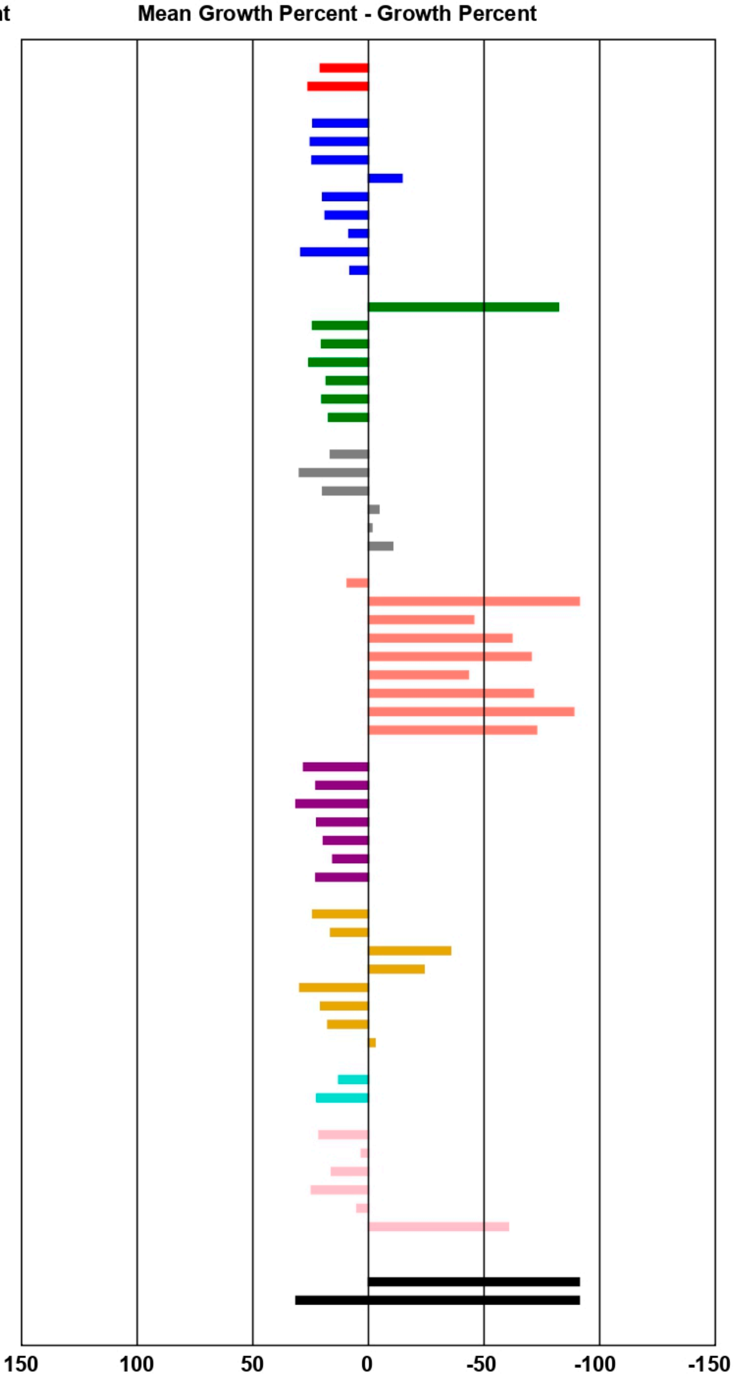

Figure S10. *In vitro* antitumor activity of compound **6** against human cancer cells of 60 lines at a concentration of 100 µM

Developmental Therapeutics Program	**NSC:** D-810153 / 1	**Conc:** 1.00E-5 Molar	**Test Date:** Nov 26, 2018
One Dose Mean Graph	**Experiment ID:** 1811OS36		**Report Date:** Apr 22, 2021

Panel/Cell Line	Growth Percent
Leukemia	
CCRF-CEM	17.92
HL-60(TB)	14.49
K-562	6.51
MOLT-4	16.72
RPMI-8226	5.06
Non-Small Cell Lung Cancer	
A549/ATCC	-24.64
EKVX	-1.58
HOP-62	-13.55
HOP-92	-19.97
NCI-H226	-13.51
NCI-H23	-48.71
NCI-H322M	46.39
NCI-H460	-45.33
NCI-H522	-34.35
Colon Cancer	
COLO 205	-56.84
HCC-2998	-67.18
HCT-116	-45.63
HCT-15	-15.30
HT29	26.36
KM12	-55.61
SW-620	-26.39
CNS Cancer	
SF-268	7.10
SF-295	6.07
SF-539	-32.36
SNB-19	2.48
SNB-75	32.69
U251	-68.02
Melanoma	
LOX IMVI	-85.19
MALME-3M	-33.54
M14	-20.11
MDA-MB-435	-7.30
SK-MEL-2	-25.53
SK-MEL-28	-17.77
SK-MEL-5	-82.25
UACC-257	13.16
UACC-62	-25.57
Ovarian Cancer	
IGROV1	1.25
OVCAR-3	-43.66
OVCAR-4	18.62
OVCAR-5	-4.00
OVCAR-8	-13.55
NCI/ADR-RES	2.97
SK-OV-3	58.93
Renal Cancer	
786-0	-38.97
A498	-59.07
ACHN	-22.16
CAKI-1	9.32
RXF 393	-91.75
SN12C	-17.67
TK-10	-14.49
UO-31	-97.34
Prostate Cancer	
PC-3	-3.56
DU-145	-28.80
Breast Cancer	
MCF7	9.88
MDA-MB-231/ATCC	-30.71
HS 578T	11.24
BT-549	-20.09
T-47D	24.61
MDA-MB-468	-5.83
Mean	-17.39
Delta	79.95
Range	156.27

Mean Growth Percent - Growth Percent

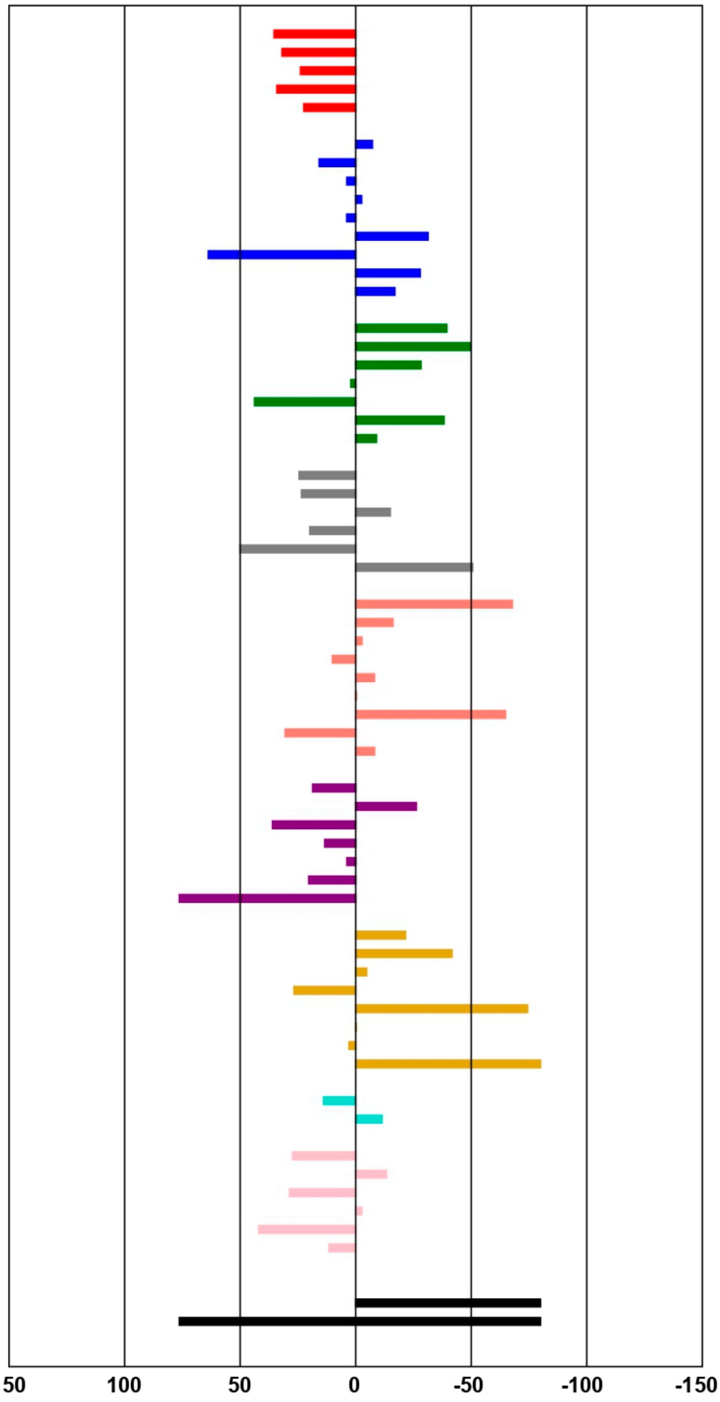

Figure S11. *In vitro* antitumor activity of compound **7** against human cancer cells of 60 lines at a concentration of 100 µM

**Developmental Therapeutics Program**	**NSC:** D-818142 / 1	**Conc:** 1.00E-5 Molar	**Test Date:** Aug 19, 2019
**One Dose Mean Graph**	**Experiment ID:** 1908OS58		**Report Date:** Sep 23, 2019

Panel/Cell Line	Growth Percent
Leukemia	
CCRF-CEM	0.95
HL-60(TB)	-30.17
K-562	-21.61
MOLT-4	-31.34
RPMI-8226	-28.73
SR	-14.07
Non-Small Cell Lung Cancer	
A549/ATCC	-9.34
EKVX	-76.32
HOP-62	-85.57
HOP-92	-79.86
NCI-H226	-44.44
NCI-H23	-48.31
NCI-H322M	-80.71
NCI-H460	-78.43
NCI-H522	-59.72
Colon Cancer	
COLO 205	-45.07
HCC-2998	-55.45
HCT-116	-87.45
HCT-15	-46.22
HT29	-45.47
KM12	-74.98
SW-620	-67.32
CNS Cancer	
SF-268	-80.80
SF-295	-98.52
SF-539	-96.48
SNB-19	-87.62
SNB-75	-98.68
U251	-87.90
Melanoma	
MALME-3M	-95.19
M14	-80.31
MDA-MB-435	-69.71
SK-MEL-2	-61.15
SK-MEL-28	-96.28
SK-MEL-5	-98.85
UACC-257	-58.90
UACC-62	-67.85
Ovarian Cancer	
IGROV1	-87.88
OVCAR-3	-93.24
OVCAR-4	-39.20
OVCAR-5	-62.74
OVCAR-8	-34.25
NCI/ADR-RES	-39.38
SK-OV-3	-23.10
Renal Cancer	
786-0	-92.26
A498	-91.17
ACHN	-100.00
CAKI-1	-78.75
RXF 393	-93.62
SN12C	-77.80
TK-10	-60.49
UO-31	-100.00
Prostate Cancer	
PC-3	-14.12
DU-145	-67.84
Breast Cancer	
MCF7	-54.19
MDA-MB-231/ATCC	-81.32
HS 578T	-49.05
BT-549	-92.12
T-47D	-43.57
MDA-MB-468	-76.12
Mean	-65.09
Delta	34.91
Range	100.95

Mean Growth Percent - Growth Percent

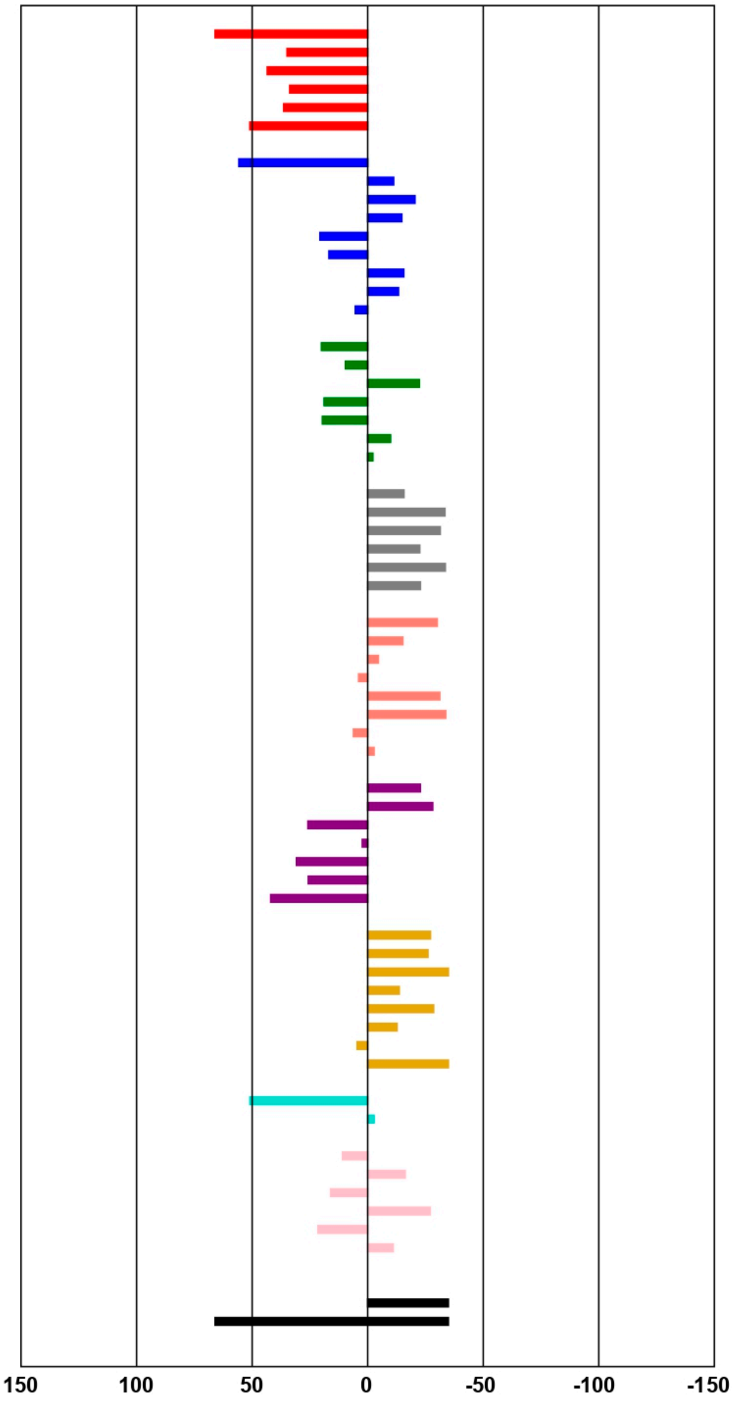

Figure S12. *In vitro* antitumor activity of compound **8** against human cancer cells of 60 lines at a concentration of 100 µM

**Developmental Therapeutics Program**	**NSC:** D-818143 / 1	**Conc:** 1.00E-5 Molar	**Test Date:** Aug 19, 2019
**One Dose Mean Graph**	**Experiment ID:** 1908OS58		**Report Date:** Sep 23, 2019

**Panel/Cell Line**	**Growth Percent**
Leukemia	
CCRF-CEM	7.57
HL-60(TB)	-2.67
K-562	18.29
MOLT-4	5.47
RPMI-8226	2.88
SR	12.35
Non-Small Cell Lung Cancer	
A549/ATCC	51.40
EKVX	85.77
HOP-62	102.96
HOP-92	66.73
NCI-H226	48.05
NCI-H23	53.20
NCI-H322M	93.90
NCI-H460	70.90
NCI-H522	47.50
Colon Cancer	
COLO 205	82.53
HCC-2998	75.76
HCT-116	3.92
HCT-15	23.22
HT29	16.53
KM12	60.52
SW-620	25.33
CNS Cancer	
SF-268	68.63
SF-295	75.30
SF-539	81.03
SNB-19	67.72
SNB-75	110.35
U251	56.61
Melanoma	
MALME-3M	29.95
M14	56.69
MDA-MB-435	72.63
SK-MEL-2	68.06
SK-MEL-28	31.54
SK-MEL-5	39.76
UACC-257	58.14
UACC-62	34.54
Ovarian Cancer	
IGROV1	85.80
OVCAR-3	49.04
OVCAR-4	26.68
OVCAR-5	114.28
OVCAR-8	38.71
NCI/ADR-RES	52.54
SK-OV-3	93.73
Renal Cancer	
786-0	84.87
A498	80.56
ACHN	32.49
CAKI-1	51.03
RXF 393	30.89
SN12C	57.75
TK-10	132.71
UO-31	56.64
Prostate Cancer	
PC-3	24.42
DU-145	68.80
Breast Cancer	
MCF7	19.18
MDA-MB-231/ATCC	76.56
HS 578T	74.77
BT-549	46.03
T-47D	24.42
MDA-MB-468	55.75
Mean	53.91
Delta	56.58
Range	135.38

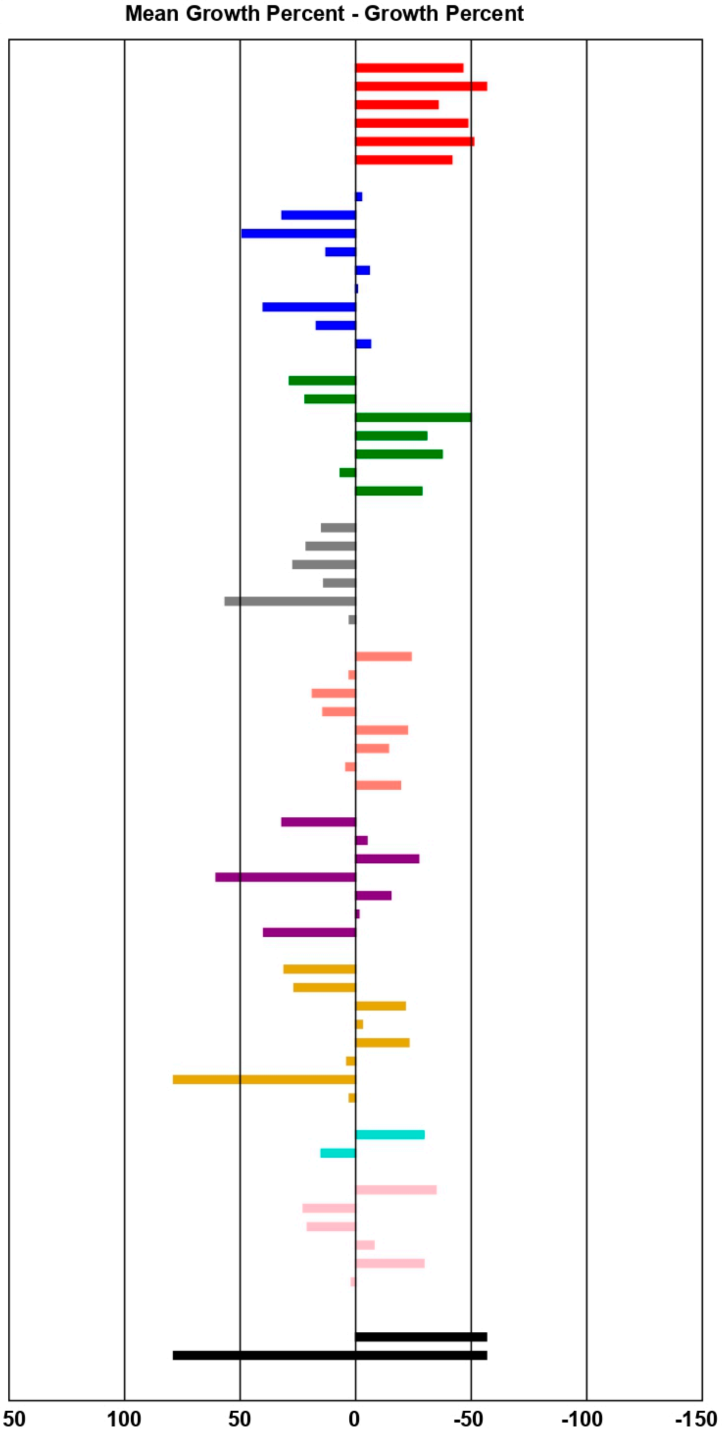

Figure S13. *In vitro* anticancer activity of compound **6** against 60 human cancer cell lines in concentrations 0.01 – 100 µM

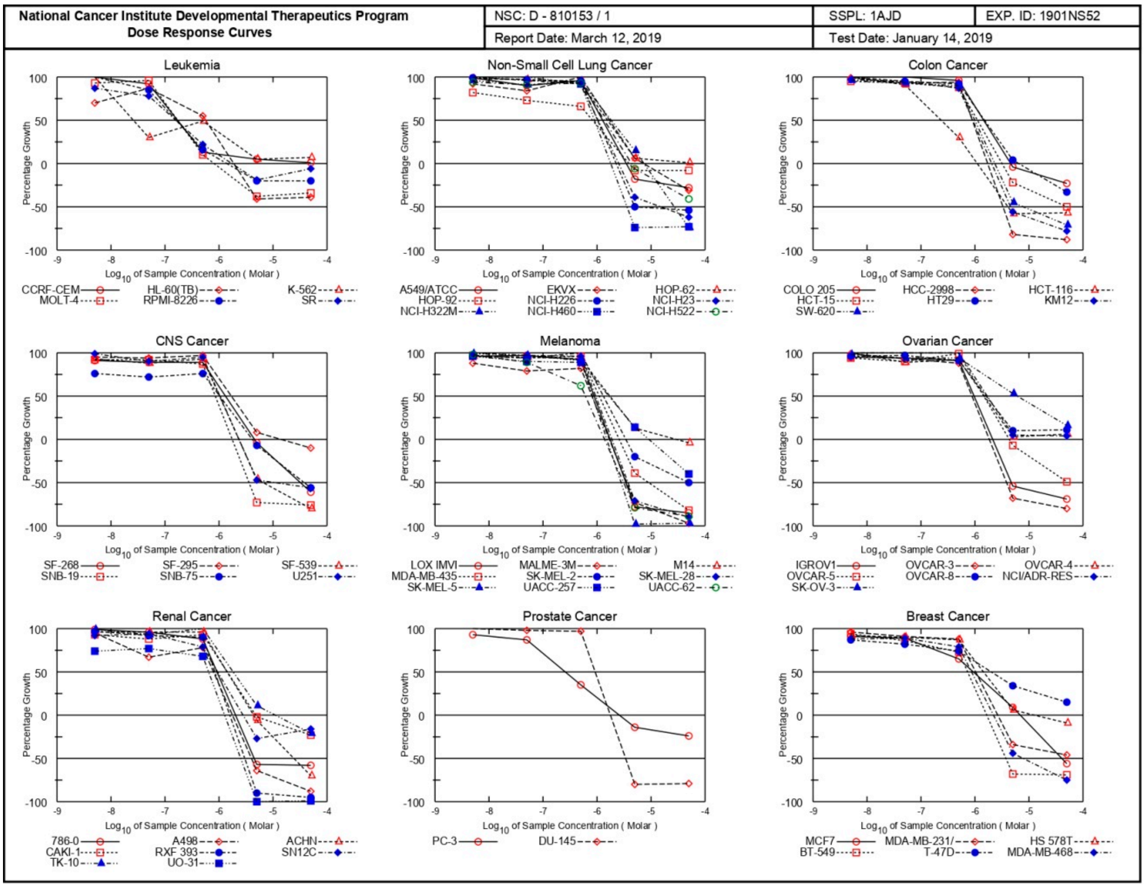

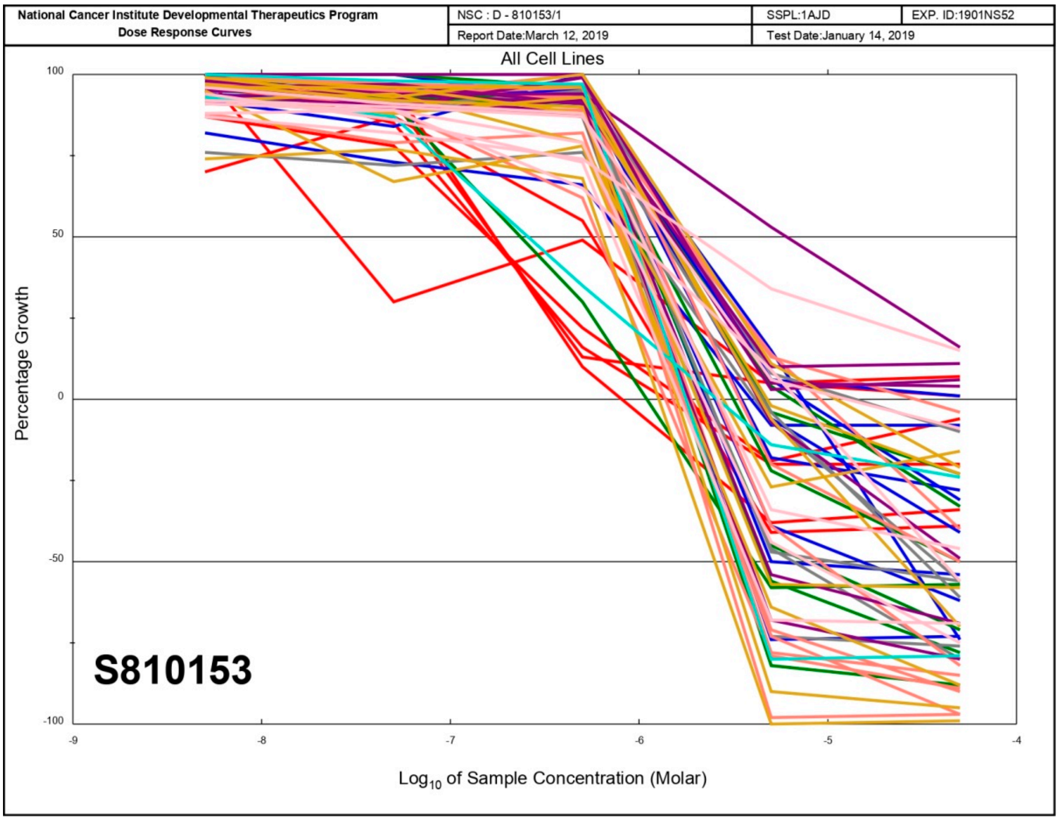

National Cancer Institute Developmental Therapeutics Program
In-Vitro Testing Results

NSC : D - 810153 / 1	Experiment ID : 1901NS52	Test Type : 08	Units : Molar
Report Date : March 12, 2019	Test Date : January 14, 2019	QNS :	MC :
COMI : 3-oxo-F-2sr-3NK	Stain Reagent : SRB Dual-Pass Related	SSPL : 1AJD	

			Log10 Concentration													
	Time		Mean Optical Densities					Percent Growth								
Panel/Cell Line	Zero	Ctrl	-8.3	-7.3	-6.3	-5.3	-4.3	-8.3	-7.3	-6.3	-5.3	-4.3	GI50	TGI	LC50
Leukemia															
CCRF-CEM	0.437	2.443	2.467	2.275	0.696	0.535	0.466	101	92	13	5	1	1.69E-7	> 5.00E-5	> 5.00E-5
HL-60(TB)	0.908	2.684	2.159	2.449	1.880	0.538	0.550	70	87	55	-41	-39	5.60E-7	1.87E-6	> 5.00E-5
K-562	0.163	1.581	1.618	0.585	0.855	0.228	0.258	103	30	49	5	7	2.64E-8	> 5.00E-5	> 5.00E-5
MOLT-4	0.709	2.732	2.595	2.652	0.917	0.443	0.471	93	96	10	-38	-34	1.72E-7	8.21E-7	> 5.00E-5
RPMI-8226	0.901	2.675	2.681	2.405	1.182	0.718	0.719	100	85	16	-20	-20	1.60E-7	1.37E-6	> 5.00E-5
SR	0.448	1.554	1.415	1.308	0.696	0.364	0.420	87	78	22	-19	-6	1.58E-7	1.75E-6	> 5.00E-5
Non-Small Cell Lung Cancer															
A549/ATCC	0.420	2.203	2.185	2.018	2.136	0.346	0.303	99	90	96	-18	-28	1.27E-6	3.50E-6	> 5.00E-5
EKVX	0.753	1.663	1.587	1.521	1.669	0.810	0.518	92	84	101	6	-31	1.72E-6	7.32E-6	> 5.00E-5
HOP-62	0.758	2.664	2.624	2.605	2.530	0.874	0.784	98	97	93	6	1	1.56E-6	> 5.00E-5	> 5.00E-5
HOP-92	1.219	2.045	1.895	1.820	1.764	1.125	1.127	82	73	66	-8	-8	8.23E-7	3.92E-6	> 5.00E-5
NCI-H226	1.223	2.808	2.798	2.756	2.722	0.607	0.557	99	97	95	-50	-54	1.02E-6	2.25E-6	4.97E-6
NCI-H23	0.599	2.141	2.144	2.079	2.015	0.363	0.230	100	96	92	-39	-62	1.04E-6	2.50E-6	1.49E-5
NCI-H322M	0.663	1.933	1.884	1.816	1.864	0.857	0.171	96	91	95	15	-74	1.82E-6	7.41E-6	2.68E-5
NCI-H460	0.429	2.805	2.848	2.825	2.613	0.113	0.115	102	101	92	-74	-73	8.95E-7	1.79E-6	3.59E-6
NCI-H522	0.888	2.628	2.522	2.469	2.503	0.839	0.520	94	91	93	-6	-41	1.36E-6	4.39E-6	> 5.00E-5
Colon Cancer															
COLO 205	0.484	2.048	2.098	2.068	1.983	0.463	0.371	103	101	96	-4	-23	1.43E-6	4.53E-6	> 5.00E-5
HCC-2998	0.731	2.671	2.654	2.537	2.560	0.133	0.089	99	93	94	-82	-88	8.92E-7	1.72E-6	3.30E-6
HCT-116	0.236	2.490	2.496	2.289	0.903	0.099	0.102	100	91	30	-58	-57	2.33E-7	1.09E-6	4.05E-6
HCT-15	0.249	1.679	1.611	1.578	1.508	0.195	0.126	95	93	88	-22	-50	1.11E-6	3.17E-6	> 5.00E-5
HT29	0.259	1.856	1.873	1.777	1.732	0.320	0.174	101	95	92	4	-33	1.50E-6	6.34E-6	> 5.00E-5
KM12	0.581	2.749	2.686	2.648	2.477	0.256	0.126	97	95	87	-56	-78	9.12E-7	2.03E-6	4.54E-6
SW-620	0.450	1.863	1.800	1.758	1.716	0.247	0.130	96	93	90	-45	-71	9.84E-7	2.31E-6	7.65E-6
CNS Cancer															
SF-268	0.949	2.458	2.327	2.294	2.287	0.911	0.372	91	89	89	-4	-61	1.31E-6	4.53E-6	3.23E-5
SF-295	0.933	3.000	2.907	2.872	2.947	1.089	0.842	95	94	97	8	-10	1.69E-6	1.37E-5	> 5.00E-5
SF-539	0.585	2.121	2.012	1.943	2.019	0.313	0.115	93	88	93	-46	-80	1.02E-6	2.33E-6	6.34E-6
SNB-19	0.780	2.517	2.370	2.374	2.295	0.208	0.185	92	92	87	-73	-76	8.53E-7	1.75E-6	3.58E-6
SNB-75	1.190	2.041	1.839	1.803	1.835	1.105	0.527	76	72	76	-7	-56	1.02E-6	4.10E-6	3.81E-5
U251	0.361	1.860	1.843	1.717	1.791	0.193	0.159	99	90	95	-47	-56	1.04E-6	2.35E-6	1.17E-5
Melanoma															
LOX IMVI	0.445	2.927	2.837	2.841	2.733	0.098	0.066	96	97	92	-78	-85	8.85E-7	1.74E-6	3.42E-6
MALME-3M	0.876	1.979	1.842	1.743	1.777	0.233	0.024	88	79	82	-73	-97	8.00E-7	1.68E-6	3.53E-6
M14	0.547	2.493	2.408	2.401	2.366	0.808	0.526	96	95	93	13	-4	1.74E-6	2.99E-5	> 5.00E-5
MDA-MB-435	0.766	2.919	2.848	2.795	2.825	0.467	0.137	97	94	96	-39	-82	1.09E-6	2.57E-6	8.98E-6
SK-MEL-2	1.055	2.781	2.719	2.675	2.785	0.841	0.527	96	94	100	-20	-50	1.30E-6	3.39E-6	4.98E-5
SK-MEL-28	0.812	2.498	2.505	2.450	2.429	0.240	0.085	100	97	96	-71	-90	9.44E-7	1.88E-6	3.76E-6
SK-MEL-5	0.982	3.242	3.221	3.181	3.036	0.024	0.029	99	97	91	-98	-97	8.24E-7	1.52E-6	2.80E-6
UACC-257	1.152	2.628	2.566	2.484	2.471	1.364	0.689	96	90	89	14	-40	1.67E-6	9.16E-6	> 5.00E-5
UACC-62	1.153	3.010	2.968	2.813	2.296	0.246	0.125	98	89	62	-79	-89	6.04E-7	1.37E-6	3.12E-6
Ovarian Cancer															
IGROV1	0.550	2.231	2.141	2.127	2.079	0.254	0.169	95	94	91	-54	-69	9.59E-7	2.12E-6	4.71E-6
OVCAR-3	0.634	2.100	2.080	1.991	1.929	0.203	0.127	99	93	88	-68	-80	8.79E-7	1.84E-6	3.84E-6
OVCAR-4	0.694	1.533	1.536	1.444	1.468	0.718	0.747	100	89	92	3	6	1.49E-6	> 5.00E-5	> 5.00E-5
OVCAR-5	0.617	1.777	1.711	1.658	1.769	0.576	0.316	94	90	99	-7	-49	1.46E-6	4.32E-6	> 5.00E-5
OVCAR-8	0.474	2.182	2.112	2.133	2.035	0.638	0.664	96	97	91	10	11	1.60E-6	> 5.00E-5	> 5.00E-5
NCI/ADR-RES	0.543	1.864	1.876	1.860	1.881	0.608	0.602	101	100	101	5	4	1.70E-6	> 5.00E-5	> 5.00E-5
SK-OV-3	0.914	2.247	2.217	2.171	2.166	1.616	1.126	98	94	94	53	16	5.90E-6	> 5.00E-5	> 5.00E-5
Renal Cancer															
786-0	0.836	2.935	2.923	2.848	2.682	0.362	0.351	99	96	88	-57	-58	9.15E-7	2.03E-6	4.49E-6
A498	2.033	2.648	2.619	2.447	2.511	0.726	0.240	95	67	78	-64	-88	7.83E-7	1.76E-6	3.97E-6
ACHN	0.450	1.967	1.913	1.908	1.907	0.424	0.136	96	96	96	-6	-70	1.41E-6	4.38E-6	2.44E-5
CAKI-1	0.880	2.478	2.368	2.287	2.369	0.863	0.676	93	88	93	-2	-23	1.42E-6	4.77E-6	> 5.00E-5
RXF 393	0.904	1.517	1.501	1.468	1.456	0.090	0.048	97	92	90	-90	-95	8.34E-7	1.58E-6	2.99E-6
SN12C	0.660	2.827	2.660	2.688	2.368	0.481	0.552	92	94	79	-27	-16	9.35E-7	2.77E-6	> 5.00E-5
TK-10	0.576	2.044	2.031	1.941	2.042	0.744	0.455	99	93	100	11	-21	1.83E-6	1.12E-5	> 5.00E-5
UO-31	1.055	2.370	2.026	2.065	1.952	.	0.008	74	77	68	-100	-99	6.41E-7	1.27E-6	2.52E-6
Prostate Cancer															
PC-3	0.610	2.557	2.411	2.300	1.286	0.528	0.462	93	87	35	-14	-24	2.55E-7	2.62E-6	> 5.00E-5
DU-145	0.500	2.020	2.099	1.994	1.980	0.101	0.106	105	98	97	-80	-79	9.25E-7	1.77E-6	3.39E-6
Breast Cancer															
MCF7	0.494	2.442	2.274	2.226	1.752	0.665	0.219	91	89	65	9	-56	9.12E-7	6.84E-6	4.08E-5
MDA-MB-231/ATCC	0.676	1.663	1.624	1.574	1.544	0.449	0.367	96	91	88	-34	-46	1.03E-6	2.64E-6	> 5.00E-5
HS 578T	1.244	2.193	2.115	2.099	2.072	1.302	1.129	92	90	87	6	-9	1.44E-6	1.24E-5	> 5.00E-5
BT-549	1.117	2.299	2.226	2.132	1.984	0.360	0.344	94	86	73	-68	-69	7.32E-7	1.65E-6	3.74E-6
T-47D	0.804	2.255	2.063	1.999	1.880	1.294	1.026	87	82	74	34	15	1.98E-6	> 5.00E-5	> 5.00E-5
MDA-MB-468	0.998	1.613	1.538	1.545	1.483	0.560	0.253	88	89	79	-44	-75	8.59E-7	2.19E-6	7.87E-6

National Cancer Institute Developmental Therapeutics Program Mean Graphs	NSC : D - 810153/1	Units :Molar	SSPL :1AJD	EXP. ID :1901NS52
	Report Date :March 12, 2019		Test Date :January 14, 2019	

Panel/Cell Line	Log_{10}GI50	Log_{10}TGI	Log_{10}LC50
**Leukemia**			
CCRF-CEM	-6.77	> -4.30	> -4.30
HL-60(TB)	-6.25	-5.73	> -4.30
K-562	-7.58	> -4.30	> -4.30
MOLT-4	-6.76	-6.09	> -4.30
RPMI-8226	-6.80	-5.86	> -4.30
SR	-6.80	-5.76	> -4.30
**Non-Small Cell Lung Cancer**			
A549/ATCC	-5.89	-5.46	> -4.30
EKVX	-5.76	-5.14	> -4.30
HOP-62	-5.81	> -4.30	> -4.30
HOP-92	-6.08	-5.41	> -4.30
NCI-H226	-5.99	-5.65	-5.30
NCI-H23	-5.98	-5.60	-4.83
NCI-H322M	-5.74	-5.13	-4.57
NCI-H460	-6.05	-5.75	-5.44
NCI-H522	-5.87	-5.36	> -4.30
**Colon Cancer**			
COLO 205	-5.84	-5.34	> -4.30
HCC-2998	-6.05	-5.77	-5.48
HCT-116	-6.63	-5.96	-5.39
HCT-15	-5.95	-5.50	> -4.30
HT29	-5.82	-5.20	> -4.30
KM12	-6.04	-5.69	-5.34
SW-620	-6.01	-5.64	-5.12
**CNS Cancer**			
SF-268	-5.88	-5.34	-4.49
SF-295	-5.77	-4.86	> -4.30
SF-539	-5.99	-5.63	-5.20
SNB-19	-6.07	-5.76	-5.45
SNB-75	-5.99	-5.39	-4.42
U251	-5.98	-5.63	-4.93
**Melanoma**			
LOX IMVI	-6.05	-5.76	-5.47
MALME-3M	-6.10	-5.77	-5.45
M14	-5.76	-4.52	> -4.30
MDA-MB-435	-5.96	-5.59	-5.05
SK-MEL-2	-5.88	-5.47	-4.30
SK-MEL-28	-6.03	-5.72	-5.42
SK-MEL-5	-6.08	-5.82	-5.55
UACC-257	-5.78	-5.04	> -4.30
UACC-62	-6.22	-5.86	-5.51
**Ovarian Cancer**			
IGROV1	-6.02	-5.67	-5.33
OVCAR-3	-6.06	-5.74	-5.42
OVCAR-4	-5.83	> -4.30	> -4.30
OVCAR-5	-5.84	-5.36	> -4.30
OVCAR-8	-5.80	> -4.30	> -4.30
NCI/ADR-RES	-5.77	> -4.30	> -4.30
SK-OV-3	-5.23	> -4.30	> -4.30
**Renal Cancer**			
786-0	-6.04	-5.69	-5.35
A498	-6.11	-5.75	-5.40
ACHN	-5.85	-5.36	-4.61
CAKI-1	-5.85	-5.32	> -4.30
RXF 393	-6.08	-5.80	-5.52
SN12C	-6.03	-5.56	> -4.30
TK-10	-5.74	-4.95	> -4.30
UO-31	-6.19	-5.90	-5.60
**Prostate Cancer**			
PC-3	-6.59	-5.58	> -4.30
DU-145	-6.03	-5.75	-5.47
**Breast Cancer**			
MCF7	-6.04	-5.17	-4.39
MDA-MB-231/ATCC	-5.99	-5.58	> -4.30
HS 578T	-5.84	-4.91	> -4.30
BT-549	-6.14	-5.78	-5.43
T-47D	-5.70	> -4.30	> -4.30
MDA-MB-468	-6.07	-5.66	-5.10
MID	-6.05	-5.37	-4.75
Delta	1.53	0.72	0.85
Range	2.35	1.79	1.3

Figure S14. *In vitro* anticancer activity of compound **7** against 60 human cancer cell lines in concentrations 0.01 – 100 µM

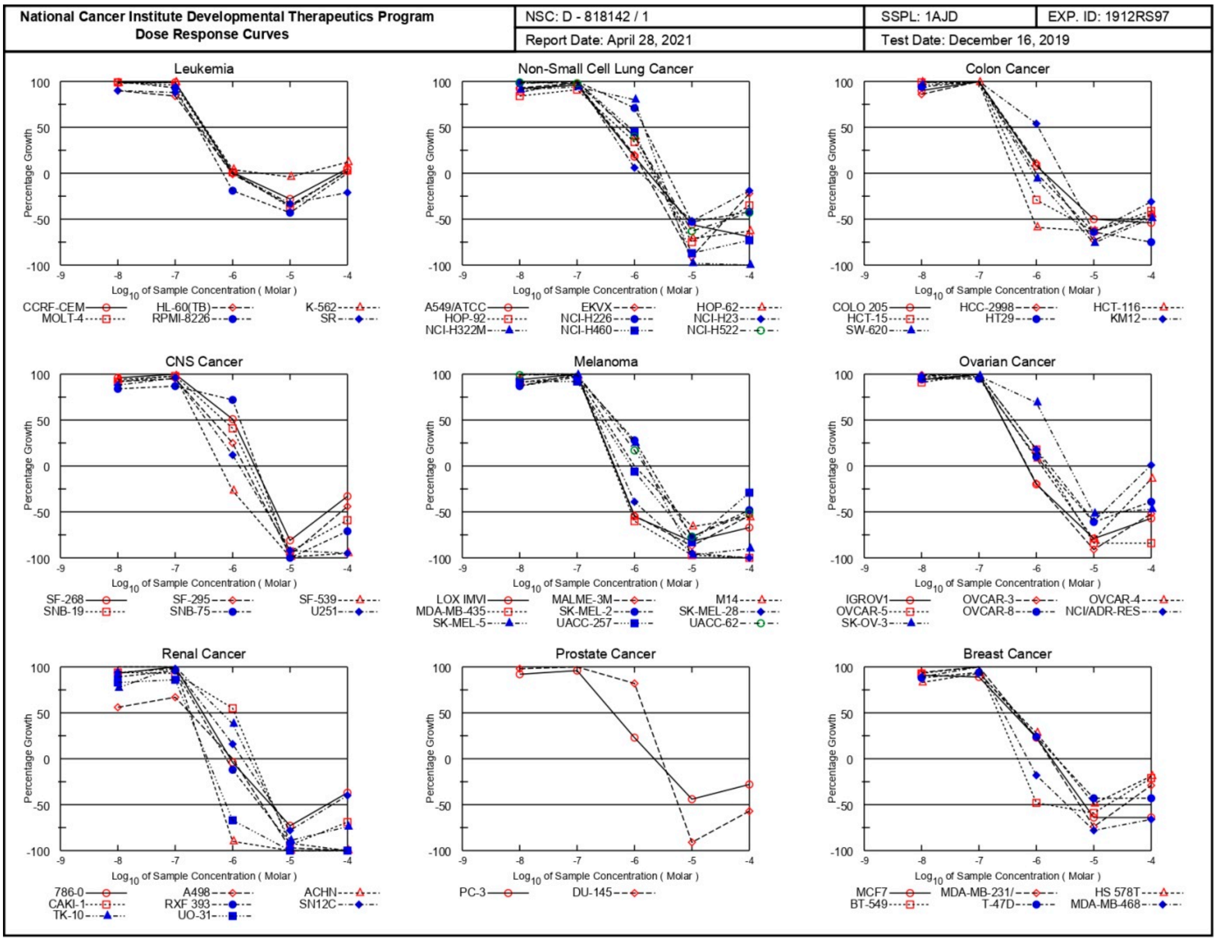

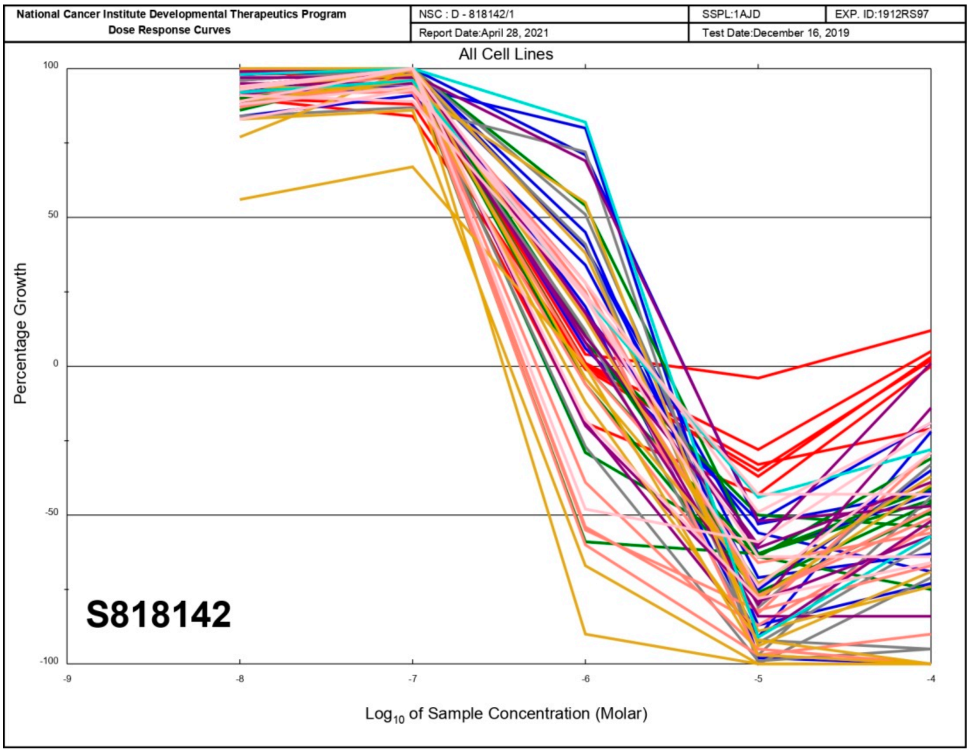

National Cancer Institute Developmental Therapeutics Program
In-Vitro Testing Results

NSC : D - 818142 / 1	Experiment ID : 1912RS97	Test Type : 08	Units : Molar
Report Date : April 28, 2021	Test Date : December 16, 2019	QNS :	MC :
COMI : OMeBk-2,30-3NK	Stain Reagent : SRB Dual-Pass Related	SSPL : 1AJD	

Log10 Concentration

	Time		Mean Optical Densities					Percent Growth							
Panel/Cell Line	Zero	Ctrl	-8.0	-7.0	-6.0	-5.0	-4.0	-8.0	-7.0	-6.0	-5.0	-4.0	GI50	TGI	LC50
Leukemia															
CCRF-CEM	0.487	3.148	3.131	3.111	0.506	0.351	0.627	99	99	1	-28	5	3.14E-7	.	> 1.00E-4
HL-60(TB)	0.406	2.016	1.850	1.752	0.401	0.262	0.432	90	84	-1	-35	2	2.48E-7	.	> 1.00E-4
K-562	0.188	2.259	2.216	2.245	0.264	0.180	0.434	98	99	4	-4	12	3.28E-7	.	> 1.00E-4
MOLT-4	0.547	2.729	2.710	2.647	0.558	0.346	0.619	99	96	1	-37	3	3.04E-7	.	> 1.00E-4
RPMI-8226	0.785	3.076	3.082	2.907	0.639	0.447	0.781	100	93	-19	-43	.	2.42E-7	6.80E-7	> 1.00E-4
SR	0.274	1.575	1.441	1.413	0.272	0.183	0.218	90	88	.	-33	-21	2.66E-7	9.81E-7	> 1.00E-4
Non-Small Cell Lung Cancer															
A549/ATCC	0.451	2.512	2.349	2.424	0.816	0.201	0.142	92	96	18	-56	-69	3.85E-7	1.74E-6	8.40E-6
EKVX	0.739	2.179	2.073	2.157	1.021	0.065	0.575	93	98	20	-91	-22	4.12E-7	1.50E-6	.
HOP-62	0.765	2.739	2.505	2.796	1.551	0.220	0.282	88	103	40	-71	-63	6.89E-7	2.28E-6	6.44E-6
HOP-92	1.240	2.019	1.892	1.946	1.507	0.307	0.801	84	91	34	-75	-35	5.26E-7	2.06E-6	.
NCI-H226	0.851	1.506	1.496	1.552	1.316	0.404	0.495	98	107	71	-53	-42	1.48E-6	3.76E-6	.
NCI-H23	0.613	2.131	2.105	2.156	0.700	0.297	0.499	98	102	6	-52	-19	3.46E-7	1.26E-6	.
NCI-H322M	0.736	2.174	2.051	2.095	1.888	0.017	-0.100	91	94	80	-98	-100	1.48E-6	2.82E-6	5.39E-6
NCI-H460	0.403	3.227	3.239	3.248	1.686	0.053	0.108	100	101	45	-87	-73	8.27E-7	2.20E-6	5.26E-6
NCI-H522	1.646	3.270	3.251	3.231	2.289	0.610	0.941	99	98	40	-63	-43	6.61E-7	2.43E-6	.
Colon Cancer															
COLO 205	0.438	2.051	1.886	2.088	0.561	0.219	0.202	90	102	8	-50	-54	3.57E-7	1.35E-6	9.95E-6
HCC-2998	0.812	2.701	2.427	2.713	1.023	0.218	0.441	86	101	11	-73	-46	3.68E-7	1.36E-6	.
HCT-116	0.279	2.470	2.439	2.447	0.114	0.104	0.154	99	99	-59	-63	-45	2.04E-7	4.23E-7	.
HCT-15	0.280	1.661	1.649	1.661	0.199	0.104	0.166	99	100	-29	-63	-41	2.44E-7	5.96E-7	.
HT29	0.402	2.535	2.417	2.543	0.411	0.143	0.102	94	100	.	-64	-75	3.19E-7	1.02E-6	5.99E-6
KM12	0.607	3.127	3.136	3.120	1.961	0.216	0.420	100	100	54	-64	-31	1.08E-6	2.85E-6	.
SW-620	0.319	2.556	2.469	2.588	0.299	0.076	0.164	96	101	-6	-76	-49	3.00E-7	8.72E-7	.
CNS Cancer															
SF-268	0.931	3.000	2.915	3.004	1.981	0.175	0.626	96	100	51	-81	-33	1.01E-6	2.42E-6	.
SF-295	0.443	2.244	2.108	2.159	0.887	0.012	0.246	92	95	25	-97	-44	4.37E-7	1.59E-6	.
SF-539	0.878	3.028	2.890	2.982	0.641	0.010	0.046	94	98	-27	-99	-95	2.42E-7	6.08E-7	2.09E-6
SNB-19	0.591	2.211	2.086	2.181	1.250	0.044	0.240	92	98	41	-93	-59	6.88E-7	2.02E-6	4.79E-6
SNB-75	2.041	3.206	3.016	3.059	2.883	-0.001	0.601	84	87	72	-100	-71	1.35E-6	2.63E-6	5.12E-6
U251	0.464	2.326	2.110	2.261	0.697	0.035	0.023	88	96	12	-92	-95	3.58E-7	1.32E-6	3.94E-6
Melanoma															
LOX IMVI	0.303	2.218	2.102	2.216	0.137	0.055	0.099	94	100	-55	-82	-67	2.10E-7	4.42E-7	9.31E-7
MALME-3M	0.604	1.508	1.388	1.512	0.277	0.079	0.277	87	100	-54	-87	-54	2.12E-7	4.46E-7	9.39E-7
M14	0.497	2.229	2.077	2.195	0.504	0.170	0.218	91	98	.	-66	-56	3.10E-7	1.01E-6	5.77E-6
MDA-MB-435	0.791	3.257	3.249	3.247	0.314	0.024	-0.179	100	100	-60	-97	-100	2.04E-7	4.20E-7	8.62E-7
SK-MEL-2	1.053	2.088	1.951	2.053	1.339	0.237	0.553	87	97	28	-77	-48	4.73E-7	1.83E-6	.
SK-MEL-28	0.642	2.067	2.112	2.141	0.389	0.033	-0.001	103	105	-39	-95	-100	2.41E-7	5.34E-7	1.55E-6
SK-MEL-5	0.996	3.318	3.307	3.297	1.576	0.029	0.103	100	99	25	-97	-90	4.60E-7	1.60E-6	4.11E-6
UACC-257	1.150	2.786	2.659	2.663	1.084	0.199	0.811	92	92	-6	-83	-29	2.71E-7	8.74E-7	.
UACC-62	0.905	3.029	3.010	3.036	1.268	0.206	0.443	99	100	17	-77	-51	4.02E-7	1.52E-6	5.14E-6
Ovarian Cancer															
IGROV1	0.436	2.117	2.014	2.145	0.351	0.090	0.187	94	102	-20	-79	-57	2.67E-7	6.89E-7	3.23E-6
OVCAR-3	0.529	1.949	1.929	2.001	0.428	0.045	0.256	99	104	-19	-91	-52	2.74E-7	6.99E-7	2.67E-6
OVCAR-4	0.815	1.905	1.877	1.868	0.902	0.162	0.701	97	97	8	-80	-14	3.35E-7	1.23E-6	.
OVCAR-5	0.564	1.416	1.342	1.417	0.716	0.091	0.089	91	100	18	-84	-84	4.07E-7	1.50E-6	4.65E-6
OVCAR-8	0.646	2.862	2.754	2.748	0.866	0.249	0.395	95	95	10	-61	-39	3.37E-7	1.38E-6	.
NCI/ADR-RES	0.454	1.655	1.632	1.720	0.675	0.181	0.465	98	105	18	-60	1	4.33E-7	.	.
SK-OV-3	0.661	1.905	1.832	1.883	1.516	0.318	0.349	94	98	69	-52	-47	1.43E-6	3.71E-6	.
Renal Cancer															
786-0	0.498	2.201	2.077	2.227	0.480	0.132	0.313	93	101	-4	-73	-37	3.09E-7	9.22E-7	.
A498	1.944	2.452	2.227	2.287	1.939	0.053	-0.127	56	67	.	-97	-100	1.81E-7	9.91E-7	3.26E-6
ACHN	0.301	1.219	1.219	1.264	0.031	-0.012	-0.098	100	105	-90	-100	-100	1.91E-7	3.46E-7	6.25E-7
CAKI-1	1.105	3.055	2.933	2.922	2.184	0.062	0.343	94	93	55	-94	-69	1.09E-6	2.34E-6	5.05E-6
RXF 393	1.595	2.466	2.372	2.429	1.402	0.123	-0.068	89	96	-12	-92	-100	2.65E-7	7.72E-7	2.97E-6
SN12C	0.525	2.341	2.225	2.311	0.821	0.114	0.315	94	98	16	-78	-40	3.88E-7	1.49E-6	.
TK-10	1.057	2.018	1.797	2.062	1.418	0.120	0.279	77	104	38	-89	-74	6.52E-7	1.98E-6	4.94E-6
UO-31	0.658	1.988	1.756	1.802	0.217	-0.005	-0.035	83	86	-67	-100	-100	1.72E-7	3.65E-7	7.74E-7
Prostate Cancer															
PC-3	0.525	2.327	2.176	2.251	0.935	0.295	0.377	92	96	23	-44	-28	4.23E-7	2.19E-6	> 1.00E-4
DU-145	0.888	3.077	3.024	3.114	2.684	0.083	0.383	98	102	82	-91	-57	1.53E-6	2.99E-6	5.82E-6
Breast Cancer															
MCF7	0.517	2.347	2.179	2.144	0.934	0.185	0.189	91	89	23	-64	-64	3.87E-7	1.83E-6	6.85E-6
MDA-MB-231/ATCC	0.686	1.726	1.662	1.775	0.929	0.181	0.487	94	105	23	-74	-29	4.71E-7	1.74E-6	.
HS 578T	1.444	2.521	2.338	2.451	1.745	0.735	1.172	83	93	28	-49	-19	4.60E-7	2.30E-6	> 1.00E-4
BT-549	0.928	2.093	2.015	2.090	0.484	0.380	0.734	93	100	-48	-59	-21	2.17E-7	4.74E-7	.
T-47D	0.994	2.307	2.153	2.230	1.313	0.563	0.571	88	94	24	-43	-43	4.29E-7	2.28E-6	> 1.00E-4
MDA-MB-468	0.779	1.485	1.410	1.500	0.641	0.170	0.264	89	102	-18	-78	-66	2.72E-7	7.12E-7	3.42E-6

National Cancer Institute Developmental Therapeutics Program Mean Graphs	NSC : D - 818142/1	Units :Molar	SSPL :1AJD	EXP. ID :1912RS97
	Report Date :April 28, 2021		Test Date :December 16, 2019	

Panel/Cell Line	Log_{10}GI50	Log_{10}TGI	Log_{10}LC50
**Leukemia**			
CCRF-CEM	-6.50		> -4.00
HL-60(TB)	-6.60		> -4.00
K-562	-6.48		> -4.00
MOLT-4	-6.52		> -4.00
RPMI-8226	-6.62	-6.17	> -4.00
SR	-6.57	-6.01	> -4.00
**Non-Small Cell Lung Cancer**			
A549/ATCC	-6.41	-5.76	-5.08
EKVX	-6.39	-5.82	
HOP-62	-6.16	-5.64	-5.19
HOP-92	-6.28	-5.69	
NCI-H226	-5.83	-5.43	
NCI-H23	-6.46	-5.90	
NCI-H322M	-5.83	-5.55	-5.27
NCI-H460	-6.08	-5.66	-5.28
NCI-H522	-6.18	-5.61	
**Colon Cancer**			
COLO 205	-6.45	-5.87	-5.00
HCC-2998	-6.43	-5.87	
HCT-116	-6.69	-6.37	
HCT-15	-6.61	-6.22	
HT29	-6.50	-5.99	-5.22
KM12	-5.97	-5.55	
SW-620	-6.52	-6.06	
**CNS Cancer**			
SF-268	-5.99	-5.62	
SF-295	-6.36	-5.80	
SF-539	-6.62	-6.22	-5.68
SNB-19	-6.16	-5.70	-5.32
SNB-75	-5.87	-5.58	-5.29
U251	-6.45	-5.88	-5.40
**Melanoma**			
LOX IMVI	-6.68	-6.35	-6.03
MALME-3M	-6.67	-6.35	-6.03
M14	-6.51	-5.99	-5.24
MDA-MB-435	-6.69	-6.38	-6.06
SK-MEL-2	-6.32	-5.74	
SK-MEL-28	-6.62	-6.27	-5.81
SK-MEL-5	-6.34	-5.80	-5.39
UACC-257	-6.57	-6.06	
UACC-62	-6.40	-5.82	-5.29
**Ovarian Cancer**			
IGROV1	-6.57	-6.16	-5.49
OVCAR-3	-6.56	-6.16	-5.57
OVCAR-4	-6.47	-5.91	
OVCAR-5	-6.39	-5.82	-5.33
OVCAR-8	-6.47	-5.86	
NCI/ADR-RES	-6.36		
SK-OV-3	-5.85	-5.43	
**Renal Cancer**			
786-0	-6.51	-6.04	
A498	-6.74	-6.00	-5.49
ACHN	-6.72	-6.46	-6.20
CAKI-1	-5.96	-5.63	-5.30
RXF 393	-6.58	-6.11	-5.53
SN12C	-6.41	-5.83	
TK-10	-6.19	-5.70	-5.31
UO-31	-6.76	-6.44	-6.11
**Prostate Cancer**			
PC-3	-6.37	-5.66	> -4.00
DU-145	-5.81	-5.52	-5.24
**Breast Cancer**			
MCF7	-6.41	-5.74	-5.16
MDA-MB-231/ATCC	-6.33	-5.76	
HS 578T	-6.34	-5.64	> -4.00
BT-549	-6.66	-6.32	
T-47D	-6.37	-5.64	> -4.00
MDA-MB-468	-6.56	-6.15	-5.47
MID	-6.4	-5.9	-5.13
Delta	0.36	0.56	1.07
Range	0.95	1.03	2.2
